# Guiding the folding of G-quadruplexes through loop residue interactions

**DOI:** 10.1093/nar/gkac549

**Published:** 2022-06-27

**Authors:** Jagannath Jana, Yoanes Maria Vianney, Nina Schröder, Klaus Weisz

**Affiliations:** Institute of Biochemistry, Universität Greifswald, D-17489 Greifswald, Germany; Institute of Biochemistry, Universität Greifswald, D-17489 Greifswald, Germany; Institute of Biochemistry, Universität Greifswald, D-17489 Greifswald, Germany; Institute of Biochemistry, Universität Greifswald, D-17489 Greifswald, Germany

## Abstract

A G-rich sequence was designed to allow folding into either a stable parallel or hybrid-type topology. With the parent sequence featuring coexisting species, various related sequences with single and double mutations and with a shortened central propeller loop affected the topological equilibrium. Two simple modifications, likewise introduced separately to all sequences, were employed to lock folds into one of the topologies without noticeable structural alterations. The unique combination of sequence mutations, high-resolution NMR structural information, and the thermodynamic stability for both topological competitors identified critical loop residue interactions. In contrast to first loop residues, which are mostly disordered and exposed to solvent in both propeller and lateral loops bridging a narrow groove, the last loop residue in a lateral three-nucleotide loop is engaged in stabilizing stacking interactions. The propensity of single-nucleotide loops to favor all-parallel topologies by enforcing a propeller-like conformation of an additional longer loop is shown to result from their preference in linking two outer tetrads of the same tetrad polarity. Taken together, the present studies contribute to a better structural and thermodynamic understanding of delicate loop interactions in genomic and artificially designed quadruplexes, e.g. when employed as therapeutics or in other biotechnological applications.

## Graphical Abstract

**Graphical Abstract:**
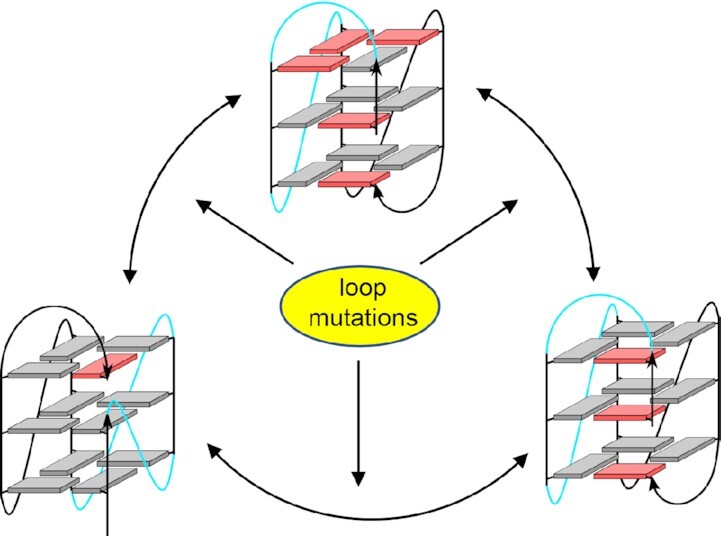
Specific loop residue interactions affect G-quadruplex structure and folding thermodynamics.

## INTRODUCTION

G-Quadruplexes (G4s) are four-stranded structures that are formed by G-rich DNA or RNA sequences. These non-canonical nucleic acids have attracted considerable attention during the last two decades due to their biological role and their potential to serve as targets for medicinal interventions (1–3). In addition, tailored G4s of a particular fold are increasingly engineered, e.g. as aptamers in G4-based therapeutic and diagnostic applications (4). Up to now, a significant number of single G4s formed by truncated G-rich sequences of biological relevance has been determined at high resolution, giving insight into specific structural features and intrinsic interactions (5–8). This also includes non-canonical architectures such as G4s with a V-shaped loop, initially characterized based on a genomic sequence (9). On the other hand, a very recent study employed an iterative integrated structural biology approach to also derive the structures of longer wild-type promoter sequences mostly intractable for high-resolution structure determinations. Notably, results suggested that more complex higher-order G4 assemblies rather than monomeric G4s may be more common and relevant within the cellular environment (10).

For reliably predicting G4 structures within the genome and for a better understanding of folding pathways in the design of specific G-rich sequences of a desirable G4 topology, an in-depth understanding of interdependent interactions that determine the overall thermodynamic stability of the system is needed. When trying to derive more general rules that determine G4 folding, studies largely rely on designed sequences that fold into a single G4. Unlike natural sequences of putative physiological relevance, the latter impose fewer restrictions when introducing more extensive mutations to systematically probe sequence dependent effects. Flanking and especially intervening sequences that form different types of loops are critical determinants of major folds and the relationship of their length and composition with favored folding pathways has been scrutinized in the past at different levels (11–16). Based solely on geometric considerations, a particular type of loop may already be excluded by the length of the intervening sequence bridging two G-core positions at varying distances in an intramolecular G4. Thus, whereas a single nucleotide between two G-tracts generally forms a stable one-nucleotide (1-nt) propeller loop upon folding into a three-layered G4, lateral and diagonal loops bridging two adjacent and distal corners of an outer G-tetrad are expected to mostly require a loop length ≥2 and ≥4 nucleotides (17,18).

In addition to simple geometric restraints set by the minimum length of loops, the collection of data for a large number of canonical G4s has also contributed to several empirical rules relating the length and distribution of loops to the most favored G4 fold (19–21). Notably, G4s with a short central loop seem to favor a parallel topology whereas G4s with a long central loop have a higher propensity of folding into an antiparallel or hybrid-type structure (22). Also, irrespective of the length of a third loop the presence of two 1-nt loops was mostly found to impose a parallel fold (23). Such a propensity of single-nucleotide loops to also enforce propeller-type conformations of additional longer loops seems to be a general phenomenon but its molecular origin remains vague. Apparently, the formation of loops upon G4 folding are interdependent and loop interactions seem to be critical determinants of the overall topology of intramolecular structures. However, relevant forces that drive a specific folding are only poorly understood and their assessment requires both a detailed structural as well as thermodynamic analysis. Lacking a better understanding of loop length and residue dependent G4 folding, a more reliable prediction of favored G4 topologies adopted by a G-rich sequence is severely hampered as is the engineering of a given G4 fold.

Intervening sequences composed of three nucleotides may likewise form propeller and lateral loops depending on the overall sequence context. Such a variability manifests itself for the human telomeric quadruplex comprising three TTA loops. Depending on flanking sequences but also on outer conditions like type of cation in the buffer solution, crystal packing, and molecular crowding effects, several folds with different loop arrangements have been reported (24–26). Thus, being of particular interest, the present study focuses on the propensity of 3-nt intervening sequences to either fold into a favored propeller or lateral loop but also on the still elusive tendency of a central 1-nt propeller loop to promote folding into an all-parallel topology. The strategy is based on the design of a G-rich sequence restricted to fold into two stable competitive G4 structures with a 3′-terminal snapback loop and on the ability to completely shift the topological equilibrium to either side by employing two different modifications. A combination of thermodynamic and NMR structural studies on various sequence mutants, also including the use of non-natural nucleoside analogs, was employed to unravel loop residue interactions that favor formation of a particular topology. Stabilization of a lateral loop through outer tetrad stacking of a loop 3′-purine but also the importance of steric effects associated with a 1-nt propeller loop bridging three tetrad layers was demonstrated. The present results provide a detailed structural and thermodynamic account for some empirical correlations reported in the recent past and enhances our understanding of G4 folding to support a better prediction but also a more rational design of G4 topologies.

## MATERIALS AND METHODS

### Materials and sample preparation

All DNA oligonucleotides were purchased from TIB MOLBIOL (Berlin, Germany) and further purified by ethanol precipitation. Concentrations were determined spectrophotometrically by measuring absorbances at 260 nm at 80°C in salt-free H_2_O with molar extinction coefficients as provided by the manufacturer based on a nearest-neighbour model (27,28). All DNA oligonucleotides were dissolved in a low-salt buffer of 10 mM potassium phosphate, pH 7. Samples were annealed with concentrations as used for the subsequent experiments by heating to 85°C for 5 min followed by slow cooling to room temperature and storage in a refrigerator overnight.

### Circular dichroism (CD)

CD spectra were acquired with a Jasco J810 spectropolarimeter. Quadruplex sequences (∼5 μM) were measured in 1-cm quartz cuvettes at 20°C. Spectra were obtained by the accumulation of five scans at a speed of 50 nm/min over a range of 220–330 nm, a bandwidth of 1 nm, and a response time of 4 s.

### UV melting and the determination of thermodynamic parameters

Melting experiments were performed in triplicate with a Jasco V-650 spectrophotometer equipped with a Peltier thermostat (Jasco, Tokyo, Japan) using quartz cuvettes of 10 mm path length. Absorbances of oligonucleotides (∼5 μM) were recorded at 295 nm from 10 to 90°C with a heating and cooling rate of 0.2°C/min. Employing a linear fit with extrapolation of upper and lower baselines, the absorbance vs. temperature curve was converted to a folded fraction α vs. temperature curve. Melting temperatures *T*_m_ with a folded fraction α = 0.5 are reported as an averaged value from heating curves of three independent measurements. With the equilibrium constant *K*(*T*) given by *K*(*T*) = (1-α(*T*))/α(*T*), thermodynamic parameters Δ*H*°, Δ*S*° and Δ*G*° were derived from a van’t Hoff analysis using the standard thermodynamic relationships ln*K*(*T*) = –Δ*H*°/R*T* + Δ*S*°/R and Δ*G*°(*T*) = Δ*H*° – *T*Δ*S*°. The analysis is based on a two-state equilibrium with the assumption of negligible heat capacity effects and temperature independent enthalpies and entropies.

### NMR spectroscopy

All NMR spectra were acquired on a Bruker Avance Neo 600 MHz spectrometer equipped with an inverse ^1^H/^13^C/^15^N/^19^F quadruple resonance cryo-probehead and z-field gradients. Unless stated otherwise, NMR spectra were acquired at 30°C in 10 mM potassium phosphate buffer with 10% D_2_O, pH 7.0. For spectral processing and analysis, Topspin 4.0.7 and CcpNmr Analysis 2.4.2 was used (29,30). Proton chemical shifts were indirectly referenced to sodium trimethlysilyl propionate (TSP) through the temperature-dependent water chemical shift at pH 7.0 and carbon chemical shifts were referenced to sodium trimethylsilylpropanesulfonate (DSS) through an indirect referencing method. An optimized WATERGATE with w5 element was employed for solvent suppression in 1D spectra and 2D nuclear Overhauser effect (NOESY) experiments. Two-dimensional NOESY spectra were recorded with mixing times of 300, 150 and 80 ms. ^1^H−^13^C heteronuclear single quantum correlation (HSQC) experiments were performed with a 3−9−19 water suppression scheme, 4K × 500 data points and a spectral width of 7500 Hz to accommodate ^13^C6/C8/C2 resonances in the indirect dimension. ^1^H−^13^C heteronuclear multiple bond correlation (HMBC) spectra were acquired with a jump-and-return water suppression, 2K × 136 data points, and processed with 50% Non-Uniform Sampling (NUS) in the indirect dimension. Double quantum filtered correlation (DQF-COSY) spectra were either recorded with water suppression through presaturation in 100% D_2_O or with a 3−9−19 water suppression in 90% H_2_O/10% D_2_O employing 2K × 512 data points.

### NMR structure calculation

Initially, 100 starting structures of lowest energy were selected from 400 structures calculated by a simulated annealing protocol in XPLOR-NIH 3.0.3 (31,32). Distance restraints were set according to crosspeak intensities in NOESY spectra. For non-exchangeable protons, intensities were categorized as strong (2.9 ± 1.1 Å), medium (4.0 ± 1.5 Å), weak (5.5 ± 1.5 Å) and very weak (6.0 ± 1.5 Å). For exchangeable protons, intensities were assigned as strong (4.0 ± 1.2 Å), medium (5.0 ± 1.2 Å) and weak (6.0 ± 1.2 Å). Glycosidic torsion angles were set to 170−310° for *anti* conformers and to 25−95° for *syn* conformers. The pseudorotation phase angle was restricted to 144–180° for *south*-type sugar puckers. G22 in *^6Br^Q* was restrained to a *north* sugar pucker with a pseudorotational angle of 0−90°. Additionally, planarity and hydrogen bond restraints were employed for bases in each G-tetrad.

Refinement was performed *in vacuo* using AMBER18 with the parmbsc force field and OL15 modifications for DNA (33). Partial atomic charges for the modified 8-bromoguanosine residues were calculated with the RED software using a DFT approach (34). The same restraints were used as before. Twenty converged lowest-energy structures were generated from 100 starting structures with a simulated annealing *invacuo*.

For a refinement in water, potassium ions were added to neutralize the system and two ions were placed in the inner channel of the G-quadruplex core between two adjacent tetrad layers. The system was hydrated with TIP3P water in a truncated octahedral box of 10 Å. The final simulation was performed at 1 atm and 300 K for 4 ns using only NMR-derived NOE distance and Hoogsteen hydrogen bond restraints. The trajectory was averaged for the last 500 ps and minimized *in vacuo* to obtain 10 lowest-energy structures. The VMD 1.9.2 software was used for the analysis of calculated structures (35). Three-dimensional structure representations were prepared with Pymol 1.8.4.

## RESULTS AND DISCUSSION

### Design of G-quadruplex forming sequences

Initially, a parent G-rich sequence 5′-GGCTAGGGTCAGGGTGGGTCAG-3′ with a truncated 5′-terminal GG-tract termed *Qref* was designed to restrict the available topological space and to better control the formation of various putative G4 species for thermodynamic and NMR spectral analyses (for all *Qref* derived oligonucleotides see Table 1). Sequences comprising a guanine-deficient GG-tract in addition to three GGG-tracts may fold into a three-layered quadruplex topology but with one tetrad bearing a vacant site. Interestingly, bioinformatics studies have shown that such sequences are highly abundant in the human genome and may potentially support an environment-responsive regulation in cellular processes (36,37). Thus, the vacant site can be filled with a guanine base from guanine-containing metabolites but also stabilized intramolecularly through snapback loop formation to form an intact G-core (38–40). Accordingly, the 3′-terminal TCAG-sequence of *Qref* was expected to form a snapback loop for filling a putative vacant position as a result of its truncated 5′-GG-tract, allowing a G4 architecture with three intact G-tetrads.

**Table 1.** Parent sequence *Qref* and *Qref***-**derived oligonucleotides with terminal 1-nt additions/deletions as well as single and double mutations^a^

Oligonucleotide	Sequence	Major topology^b^
*Qref*	5′-GG-CTA-GGG-TCA-GGG-T-GGG-TCA-G-3′	+(lpp)
*^3^* ′*^del^Q*	5′-GG-CTA-GGG-TCA-GGG-T-GGG-TCA-3′	—*^c^*
*^2Br^Q*	5′-G**BrG**-CTA-GGG-TCA-GGG-T-GGG-TCA-G-3′	+(lpp)
*^6Br^Q*	5′-GG-CTA-**BrG**GG-TCA-GGG-T-GGG-TCA-G-3′	+(lpp)
*^7Br^Q*	5′-GG-CTA-G**BrG**G-TCA-GGG-T-GGG-TCA-G-3′	—*^c^*
*^8Br^Q*	5′-GG-CTA-GG**BrG**-TCA-GGG-T-GGG-TCA-G-3′	—*^c^*
*^16Br^Q*	5′-GG-CTA-GGG-TCA-GGG-T-**BrG**GG-TCA-G-3′	+(lpp)
*^5^* ′*^T^Q*	5′-**T**-GG-CTA-GGG-TCA-GGG-T-GGG-TCA-G-3′	-(ppp)
*Q-5T*	5′-GG-CT**T**-GGG-TCA-GGG-T-GGG-TCA-G-3′	-(ppp)
*Q-5I*	5′-GG-CT**I**-GGG-TCA-GGG-T-GGG-TCA-G-3′	+(lpp)
*Q-5X^d^*	5′-GG-CT**X**-GGG-TCA-GGG-T-GGG-TCA-G-3′	-(ppp)
*Q-11T*	5′-GG-CTA-GGG-TC**T**-GGG-T-GGG-TCA-G-3′	+(lpp)
*Q-11I*	5′-GG-CTA-GGG-TC**I**-GGG-T-GGG-TCA-G-3′	+(lpp)
*Q-11X^d^*	5′-GG-CTA-GGG-TC**X**-GGG-T-GGG-TCA-G-3′	+(lpp)
*Q-5I-11I*	5′-GG-CT**I**-GGG-TC**I**-GGG-T-GGG-TCA-G-3′	+(lpp)
*Q-3T-10T*	5′-GG-**T**TA-GGG-T**T**A-GGG-T-GGG-TCA-G-3′	+(lpp)
*Q-3A*	5′-GG-**A**TA-GGG-TCA-GGG-T-GGG-TCA-G-3′	+(lpp)
*Q-3X^d^*	5′-GG-**X**TA-GGG-TCA-GGG-T-GGG-TCA-G-3′	+(lpp)

^a^Mutations are indicated by underlined bold letters.

^b^Based on imino NMR signal intensities at *T*= 30°C. ^c^Significant structural heterogeneity. ^d^X = abasic 1′,2′-dideoxyribose residue.

**Figure 1.**
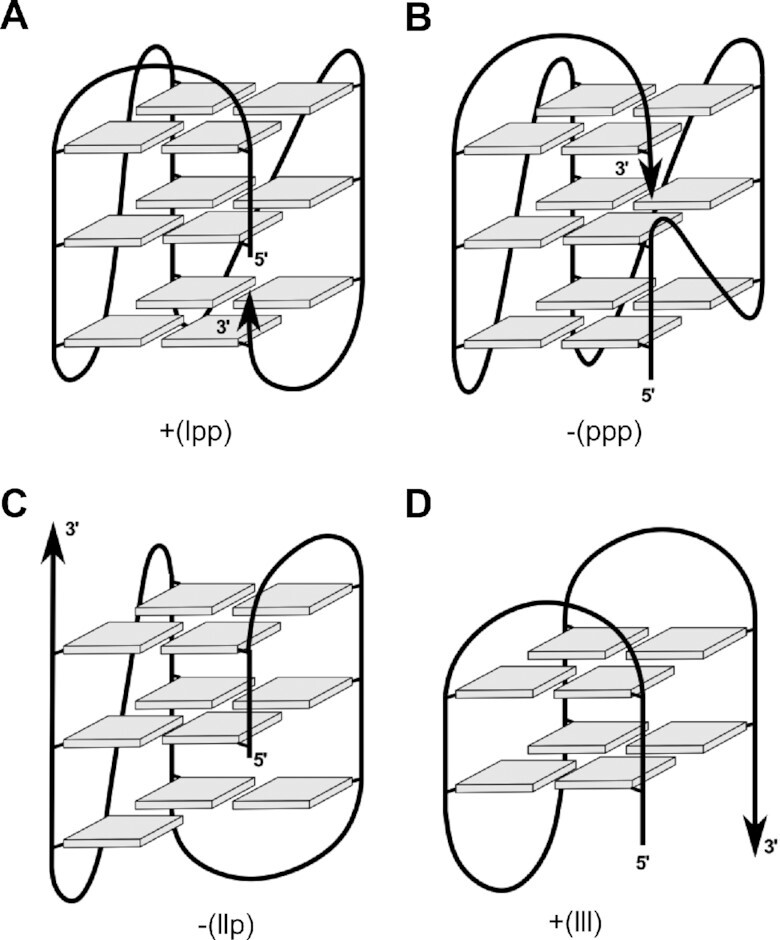
Putative G4 topologies of designed sequences given a third 1-nt propeller loop and the absence of diagonal loops with loop lengths ≤3 residues. (**A**) Three-tetrad hybrid-type +(lpp) and (**B**) parallel –(ppp) topology with snapback loop filling a vacant G-tetrad position; (**C**) hybrid-2 –(llp) G4 with a G vacancy in its lower tetrad and (**D**) two-tetrad antiparallel +(lll) G4. Stabilities of the latter two G4s are expected to be compromised by the formation of only two intact G-tetrads. Topologies are characterized by simple loop descriptors according to Webba da Silva (43,44).

Given a most stable 1-nt propeller loop linking the third and fourth G-column and the absence of any diagonal loop with available loop lengths of ≤3 nucleotides, the first two 3-nt intervening segments may either arrange in a lateral or a propeller loop, the latter required to follow the right-handed G4 helicity with a counter-clockwise progression if the 5′-terminal G-column runs towards the viewer (18,41). Also, excluding a highly unlikely propeller-type snapback loop only observed in a rather unique G4 formed by a *c-kit* promoter sequence (42), two stable topologies for a three-layered snapback loop architecture with a 3–3–1 loop length arrangement can be envisaged (Figure 1A, B). For convenience, a systematic G4 notation according to Webba da Silva was employed, classifying different topologies with a simple descriptor that only includes the type (p, l and d for propeller, lateral, and diagonal) and relative direction of loops linking G-tracts (+ and – for clockwise and counter-clockwise progression, respectively) (43,44). Thus, the two three-layered topologies are expected to either comprise a +(lpp) hybrid-type structure with one 3-nt lateral loop followed by a 3-nt and a 1-nt propeller loop (Figure 1A) or a –(ppp) parallel fold with two 3-nt and a single 1-nt propeller loop (Figure 1B). The 3′-terminal snapback loop filling the vacant position of the first G_2_-tract was omitted in the descriptors.

Other putative folds suffer from the loss of a third intact G-tetrad. A hybrid-2 topology –(llp) with two lateral loops is expected to be considerably disfavored by leaving an empty outer G-core position with the 3′-terminal snapback G located at the opposite G4 face (Figure 1C). Likewise, the stability of a putative two-tetrad antiparallel G4 +(lll) with three lateral loops is compromised by the presence of only two stacked G-tetrad layers (Figure 1D). Clearly, formation of a three-layered chair-type topology is effectively eliminated by a 1-nt intervening sequence between G_3_-tracts which is largely restrained to form a stable propeller-type loop.

It should be mentioned that there are no flanking sequences in the basic snapback loop design of *Qref*. Whereas loop interactions with overhang nucleotides have been shown to influence G4 folding as demonstrated by human telomeric sequences with different flanking residues, eliminating the impact of overhang residues reduces additional complexity to focus on loop interactions inherent to the G4 structure. Also, putative rearrangements of flanking nucleotides, e.g. in genomic DNA upon formation of quadruplex-duplex junctions may easily alter overhang interactions in unpredictable ways.

### Topology of G-quadruplex forming sequences

**Figure 2.**
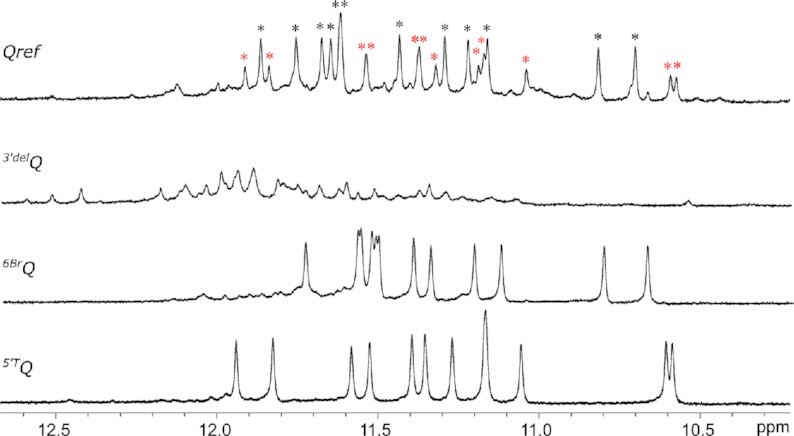
Imino proton spectral region of *Qref*, *^3^*′*^del^Q*, *^6Br^Q* and *^5^*′*^T^Q*. Black and red asterisks for *Qref* indicate imino resonances of a major and minor fold, respectively. NMR spectra were acquired in 10 mM potassium phosphate buffer, pH 7.0, at 30°C.

**Figure 3.**
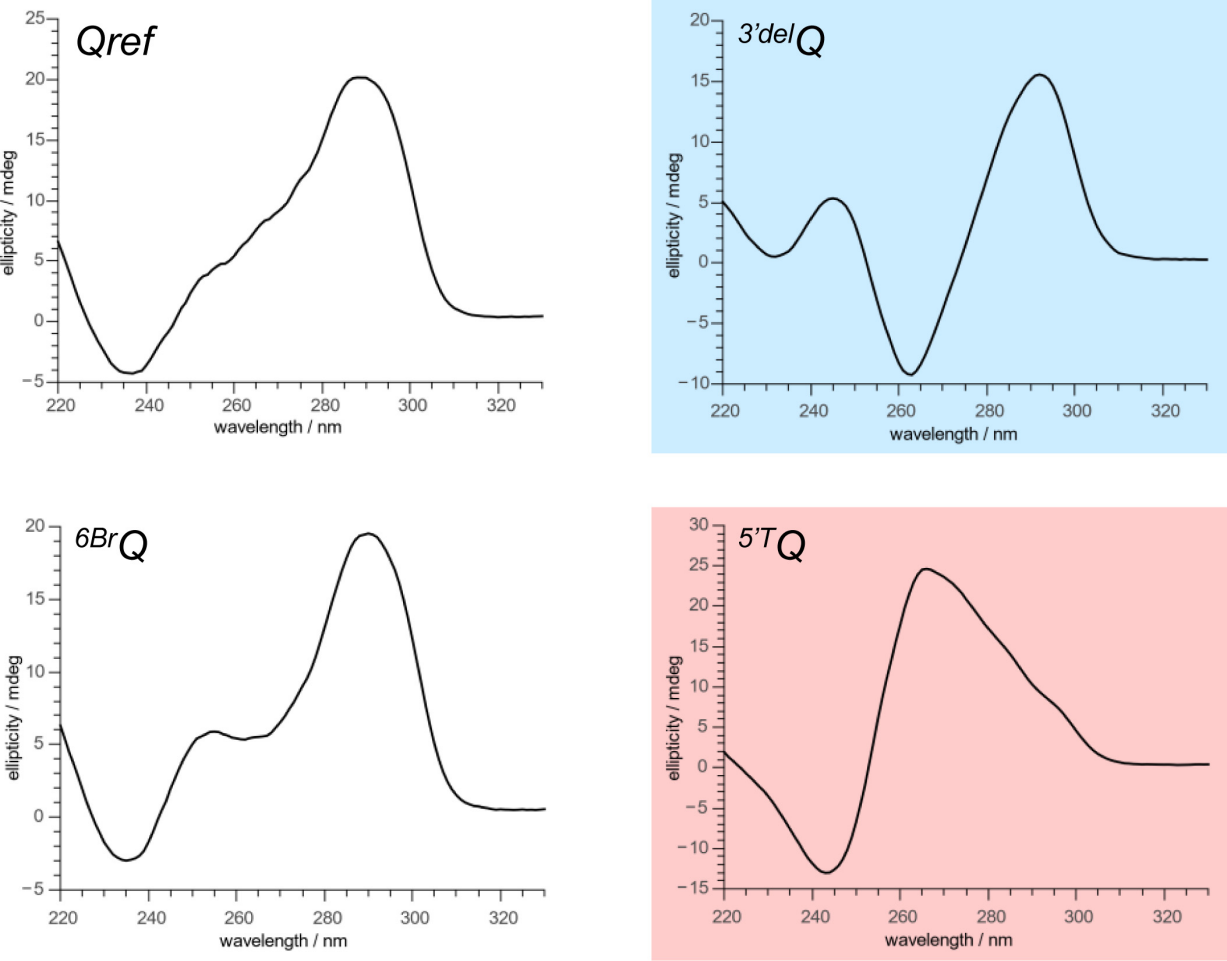
CD spectra of *Qref*, *^3^*′*^del^Q*, *^6Br^Q* and *^5^*′*^T^Q*. Whereas spectra of *Qref* and *^6Br^Q* are in line with a mixture of parallel, antiparallel, and/or hybrid-type topologies, spectra of *^3^*′*^del^Q* (blue background) and *^5^*′*^T^Q* (red background) feature a signature typical for an antiparallel and a parallel topology, respectively (20). CD spectra were acquired in 10 mM potassium phosphate buffer, pH 7.0, at 20°C.

The imino proton spectral region of parent *Qref* suggests formation of a high-populated major and a lower populated minor three-layered G-quadruplex in a molar ratio of about 1:0.4 with additional very low-intensity signals of a third species (Figure 2). Deleting the 3′-terminal G to give the truncated *^3^*′*^del^Q* sequence, imino resonances of both major and minor quadruplexes vanish and a complete re(un)folding is evidenced by downfield-shifted and significantly broadened imino signals. Although structures formed by truncated *^3^*′*^del^Q* are not amenable to a more detailed structural analysis, it can be hypothesized based on their antiparallel CD signature with minima and maxima of the amplitude at about 265 and 290 nm (Figure 3) that they include G-register isomers of two-tiered antiparallel folds such as the one shown in Figure 1D or similar to a basket-type telomeric G-quadruplex with only two G-tetrad layers called Form 3 (45). However, formation of multistranded species is also conceivable. Requiring a 3′-terminal G for folding, the *Qref* sequence is expected to form a snapback loop G4 with an interrupted first G-column and with the 3′-G residue participating in a G-tetrad.

To get more insight into the major *Qref* topology, either position 6, 7 or 8 of the second GGG-tract was selectively substituted with a *syn*-favoring 8-bromo-2′-deoxyguanosine residue ^Br^G. Whereas substitutions at positions 7 and 8 proved deleterious for the original fold with considerable shifts and broadening of imino resonances (not shown), the *^6Br^Q* sequence with a bromo-modification at position 6 featured a mostly clean imino proton spectral region composed of twelve well resolved resonances (Figure 2).

**Figure 4.**
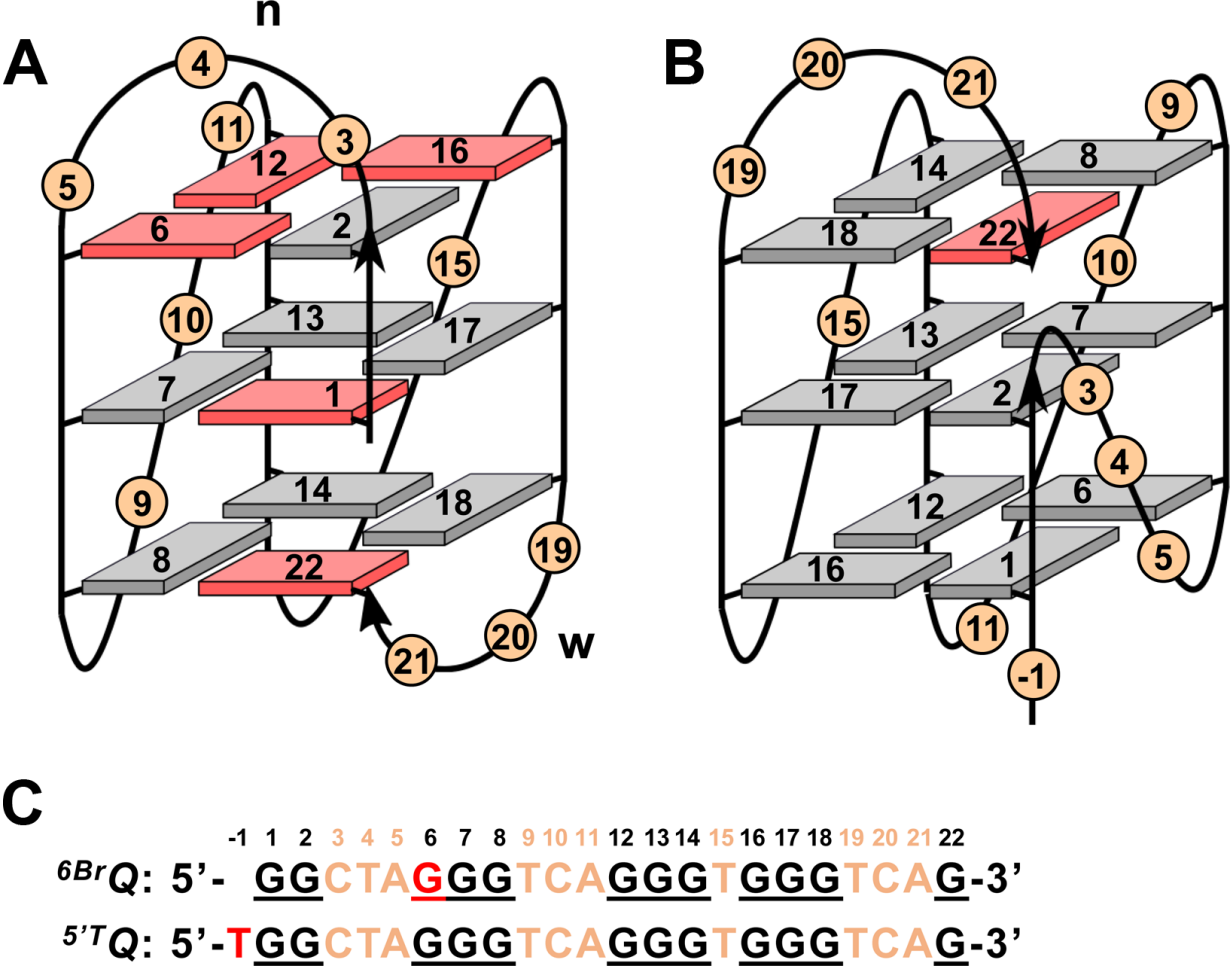
(**A**) Topologies of *^6Br^Q* and (**B**) *^5^*′*^T^Q* quadruplexes; *syn*- and *anti*-G residues are colored red and grey, n and w denote narrow and wide groove, respectively. (**C**) Sequence of *^6Br^Q* and *^5^*′*^T^Q* with residue numbering; added 5′-T and incorporated ^Br^G residue in red, intervening sequences in orange, and Gs participating in tetrad formation underlined.

A detailed NMR spectral analysis of *^6Br^Q* was based on NOESY, DQF-COSY, ^1^H–^13^C HSQC and ^1^H–^13^C HMBC experiments (for spectral assignment strategies, spectra, and chemical shifts see Supplementary Figures S1–S5 and Supplementary Table S1 in the Supporting Information). Unambiguous resonance assignments together with H/D isotope exchange experiments demonstrated folding into a +(lpp) hybrid-type topology. It comprises a single first lateral followed by two propeller loops as well as one broken *syn*−*syn*−*anti* column (G22–G1–G2) and three *syn*−*anti*−*anti* columns (G6–G7–G8, G12–G13–G14 and G16–G17–G18) (Figure 4A).

Comparison of imino proton chemical shifts and in particular observation of closely similar crosspeak patterns in 2D NMR spectra identified the topology of the ^Br^G-modified sequence to also represent the major fold of *Qref* (Supplementary Figures S6 and S7). In trying to unambiguously identify the lower populated *Qref* G4 species, a single 5′-flanking T was added to the *Qref* sequence to give *^5^*′*^T^Q*. Such an approach follows the expectation that a 5′-T overhang should not impact or rather stabilize a parallel –(ppp) fold with G1 located in the 5′-outer tetrad while preventing a hybrid-type +(lpp) topology with G1 located in the central tetrad due to steric clashes with the fill-in 3′-terminal G (see Figure 1A, B). Indeed, modified sequence *^5^*′*^T^Q* again features a clean and well resolved imino proton NMR spectral region indicative of a single G4 fold and a CD signature characteristic of a parallel topology (Figures 2 and 3). A detailed NMR spectral analysis using standard strategies demonstrated its parallel topology with three propeller loops and a terminal snapback *syn*-G residue filling the vacant position at the 3′-outer tetrad (Figure 4B; for assignment strategies, spectra, and a compilation of chemical shifts see Supplementary Figure S8-S11 and Supplementary Table S2). Again, a close inspection of *^5^*′*^T^Q* and *Qref* spectra with highly similar signal chemical shifts and crosspeak patterns identified the parallel fold to be the higher populated minor species in the *Qref* mixture (see Figure 2).

### Three-dimensional structure of *^6Br^Q* and *^5^*′*^T^Q*

Following resonance assignments, molecular dynamics simulations were performed for *^6Br^Q* and *^5^*′*^T^Q* in explicit water using NMR-derived distance and dihedral angle restraints. With rmsd values of 0.7 and 0.9 Å for *^6Br^Q* and *^5^*′*^T^Q*, respectively, the three-layered G-core is well defined for both G4s (for structural statistics and a superposition of ten lowest-energy structures see Supplementary Table S3 and Supplementary Figure S12). In general, higher rmsd values of 2.2 and 2.5 Å as calculated for all residues can be attributed to significant fluctuations of those loop residues that are mostly exposed to solvent but also partially directed towards a G4 groove. However, all residues of the snapback loop, second and third residues in the (+lpp) lateral loop bridging a narrow groove and the last 3′-residue of the 3-nt propeller loops are well defined.

**Figure 5.**
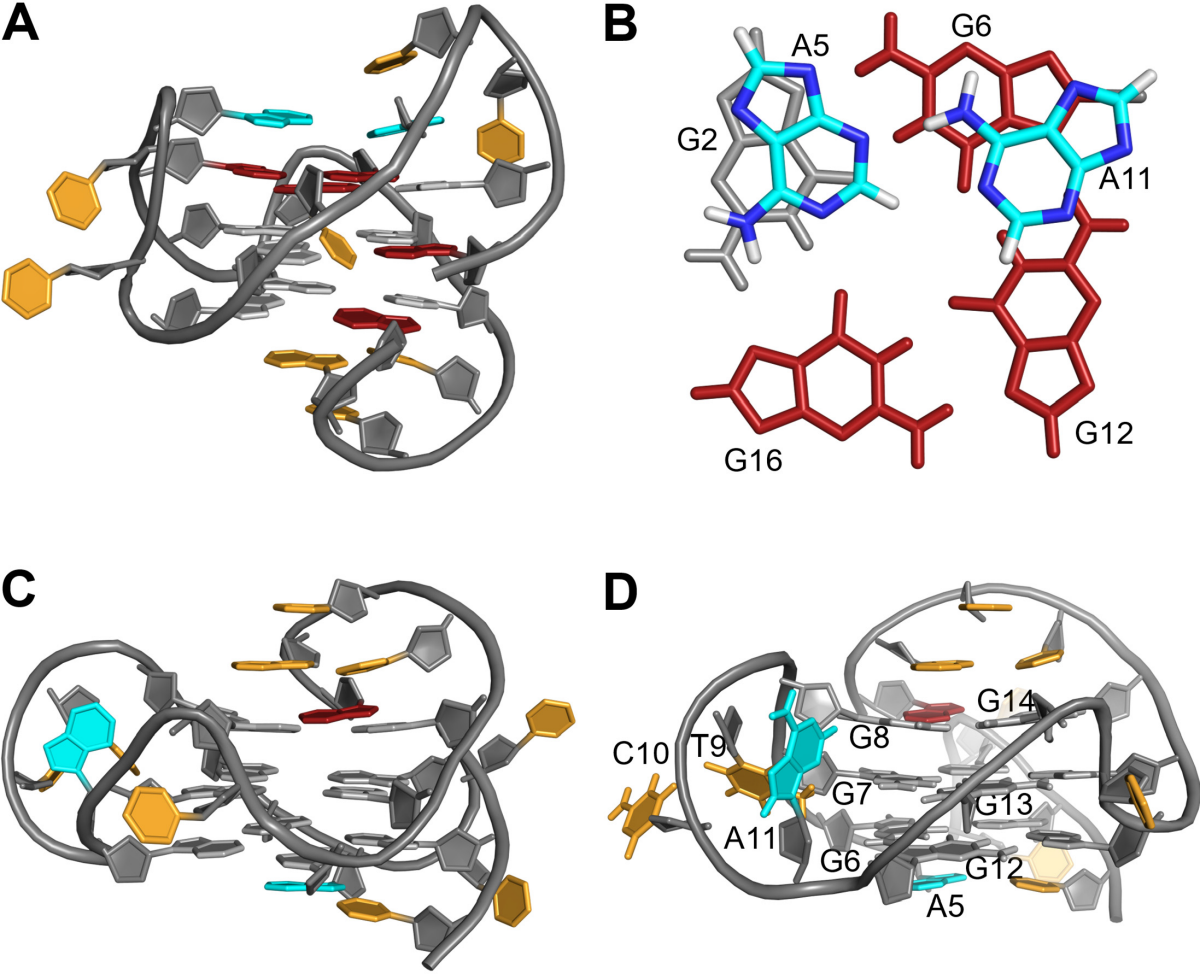
(**A**) Representative NMR structure of *^6Br^Q* and (**B**) view onto coplanar A5 and A11 residues stacked onto the upper tetrad with a putative A11 amino – A5 N3 hydrogen bond interaction. (**C**) Representative NMR structure of *^5^*′*^T^Q* and (**D**) side view into a G4 groove with inserted A11. *Anti-* and *syn*-guanosines are colored in grey and red, respectively, A5 and A11 are colored in cyan and other residues are colored in orange.

A representative structure of the *^6Br^Q* G4 is shown in Figure 5A. The 5′-terminal G1 is positioned in the central tetrad of a *syn*-*syn*-*anti* G-tract. A 3-nt lateral loop C3-T4-A5 is followed by a 3-nt propeller loop T9–C10–A11, a 1-nt propeller loop T15, and a 3-nt snapback loop with 3′-terminal G22 filling the vacant position of the first G-column. Residues of the lateral snapback loop T19–C20–A21 bridging a wide groove adopt a well-defined orientation in the structural ensemble. Thus, a T19·A21 Hoogsteen base pair caps the lower G-tetrad with additional outer stacking of C20 onto the base pair (Supplementary Figure S13A). Looking at the first 3-nt lateral and following 3-nt propeller loops, a formed capping structure onto one side of the upper G-tetrad is noticeable and corroborated by several experimental NOE restraints (Figures 5B and Supplementary Figure S14A, B). Thus, 3′-terminal loop residues A5 and A11 stack onto the tetrad, exhibiting a putative hydrogen bond interaction between the A11 amino proton and A5 N3 in most of the structures. T4 stacks over A5 that is sandwiched between the preceding T4 and G2 of the outer G-tetrad. In contrast, A11 only shows poor stacking over *syn*-G6.

A highly similar arrangement with a 3′-adenine of a 3-nt propeller loop recruited through putative base-base interactions by a 3′-adenine of a preceding lateral loop to stack onto the top G-tetrad has also been observed by a human telomeric sequence 5′-(TTAGGG)_4_TTA-3′ with a single ^Br^G modification in Na^+^ solution (46). In the formed antiparallel +(lpl) G4, the 3′-A of the lateral loop is additionally base-paired to a thymine of the 3′-flanking sequence (Supplementary Figure S14C). Likewise, stacking of a 3′-adenine base of a propeller loop onto an outer tetrad has been found in longer propeller loops if recruited by an adjacent coplanar base in a lateral loop or overhang sequence (47,48).

Calculated structures of *^5^*′*^T^Q* feature a parallel three-layered G4 with a T(–1) 5′-overhang and G1 in an outer tetrad position (Figure 5C). The G1-G2 run of the first G-column is again complemented with 3′-terminal *syn*-G22 through a lateral snapback loop. The latter adopts the same capping structure as found for *^6Br^Q* with A21 Hoogsteen hydrogen-bonded to T19 and the T·A base pair sandwiched between the lower G-tetrad and C20 (Supplementary Figure S13B). Whereas extensive disorder is observed for the first two residues of both the first and second 3-nt propeller loop, the last loop residues are well defined in their orientation as also demonstrated by various NOE contacts. Thus, A5 stacks onto the bottom tetrad below G6. However, such an arrangement may derive from the first propeller loop bridging only two tetrad layers in this snapback loop architecture. In contrast, bridging three tetrad layers in analogy to the +(lpp) topology of *^6Br^Q*, the second propeller loop features a 3′-adenine A11 that is found to insert into the G4 groove in all calculated structures while fixed by 10 NOE-based distance restraints (Figure 5D). Consequently, A11 at the 3′-position of the second 3-nt propeller loop has quite distinct orientations/interactions depending on its location within a –(ppp) or a +(lpp) topology.

### Impact of mutations on favored topologies

**Figure 6.**
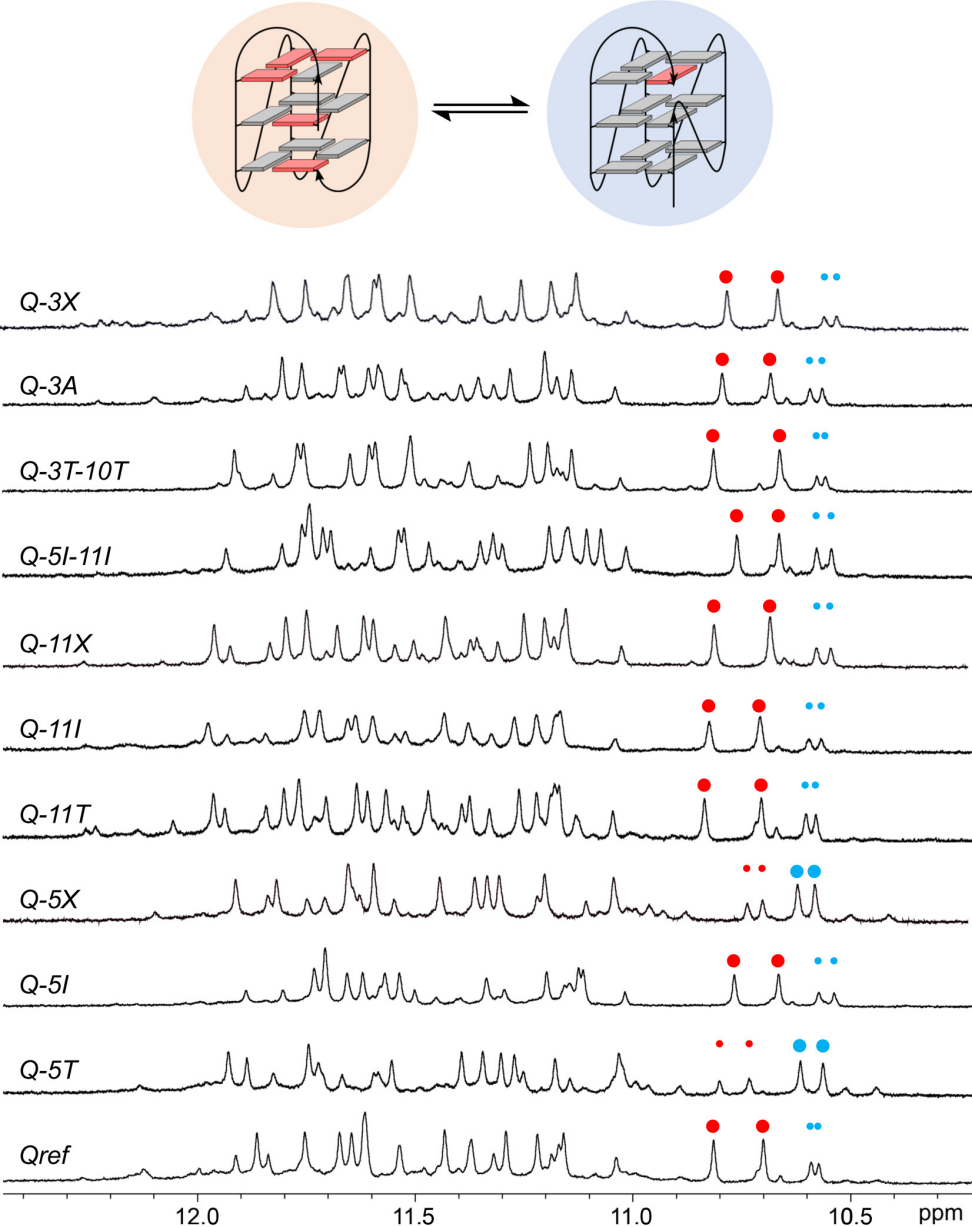
Imino proton spectral region of *Qref* mutants. NMR spectra were acquired in 10 mM potassium phosphate buffer, pH 7.0, at 30°C. Red and blue dots of larger and smaller size mark major and minor high-field shifted resonances of G8 and G22 residues in +(lpp) and –(ppp) topologies shown on top.

To further assess the participation of loop bases on (de)stabilizing interactions, single and dual base mutations were introduced into intervening sequences and their impact on the favored loop formation and G4 topological equilibria was studied (Table 1). Mutations also involved inosine (I) as a purine nucleoside substitute and 1′,2′-dideoxyribose (X) lacking a nucleobase. As shown by the imino proton NMR spectral regions, all mutant sequences generally fold into a major and minor G4 with an additional very low-populated species in most cases (Figure 6). Generally, the low signal intensity of the latter did not allow its detailed assignment without corresponding reference spectra (but see below). On the other hand, the two predominant species, formed in different molar ratios ranging between 0.3:1 up to 1:0.3, were unambiguously identified for each mutant as competing +(lpp) and -(ppp) topologies as formed by *^6Br^Q* and *^5^*′*^T^Q*, respectively. It should be mentioned that such topological assignments are based on detailed NMR spectral analyses for all mutants through 2D NOESY and ^1^H–^13^C HSQC spectra and were additionally supported by a comparison of crosspeak patterns with *^6Br^Q* and *^5^*′*^T^Q* reference spectra (see Supplementary Figure S15–S18 for exemplary spectral assignments of *Q-5T* and *Q-11T* and Supplementary Figure S19 for CD spectra of all sequences).

The topological ratio was mostly conserved in *Q-3A* and *Q-3X* with an adenosine and abasic site at position 3, associated with a 3C→3A and 3C→3X mutation. Also, replacing cytidine residues C3 and C10 in the 5′-terminal and central position of the first and second loop by T to yield the *Q-3T-10T* mutant had nearly no effect on the topological equilibrium. Notably, however, the major +(lpp) topology was even maintained when substituting A11 within the second loop for either thymidine, inosine, or an abasic residue. More dramatic effects were induced by mutations at position 5 within the first loop. Whereas a 5A→5I purine-to-purine substitution in *Q-5I* did not noticeably change populations of major and minor fold, populations were inverted in *Q-5T* and *Q-5X* to form a major -(ppp) G4. This indicates a critical role of purine bases in the 3-nt lateral loop end position for favorable stacking interactions due to their larger ring system. Although associated with a slight shift in favor of a parallel G4, corresponding dual substitutions with inosine at loop terminal positions 5 and 11 do not indicate considerable synergistic effects in *Q-5I-11I*, again conserving the major hybrid topology as observed for the parent sequence.

The high-resolution structure suggests a stabilizing impact of an A5·A11 base pair capping the outer tetrad in the +(lpp) G4 (Figure 5A, B). However, the present mutational studies emphasize the critical role of A5 but not of A11 as contributor for a major +(lpp) fold. Whereas A5 may be replaced by the purine base hypoxanthine without compromising the favored formation of a hybrid against a parallel topology, its substitution by a T5 pyrimidine and in particular by an abasic nucleotide indicates a loss of critical stacking interactions for this lateral loop position, resulting in a significant shift towards a major parallel fold.

Unexpectedly, substituting A11 for inosine, thymidine, and even for an abasic residue did hardly shift equilibria in favor of a parallel structure although A11 was shown to form a capping structure with A5 in the parent G4 (Figure 5B). Apparently, additional A11 stacking and putative hydrogen bond interactions with A5 do not provide for a selective and noticeable stabilization of the hybrid-type species.

### Impact of mutations on the thermodynamic profile for G-quadruplex formation

Signal intensities in the NMR spectra yield valuable information on the population of major and minor species formed in solution. However, a substitution-induced shift in molar ratios may result from different combinations of stabilizing and destabilizing effects on the competing topologies. It would therefore be instructive to determine thermodynamic stabilities for all mutants in each of the two coexisting topologies, i.e. parallel or hybrid-type. Employing a *syn*-affine ^Br^G6 analog or a 5′T-flanking residue successfully shifted equilibria to exclusively form a +(lpp) or -(ppp) *Qref* quadruplex. Using the same approach, all mutants were likewise modified with the expectation to yield two sets of structures comprising either hybrid or parallel topologies irrespective of the specific mutation.

Initially, the two additional sets of corresponding bromo- and 5′T-modified mutant sequences were subjected to a CD and NMR spectral analysis. Indeed, all 5′T sequences result in CD spectra typical of a parallel fold whereas ^Br^G-modified sequences exhibit CD signatures consistent with a hybrid-type topology (Supplementary Figure S20 and S21). Accordingly, imino proton spectral regions of the former display clean spectra of a parallel species (Supplementary Figure S22). NMR spectra indicating a single hybrid-type G4 are also observed for most of the ^Br^G6-modified sequences and additional low-populated species as detected for *^6Br^Q-5T* and *^6Br^Q-11T* were not expected to significantly compromise the thermodynamic characterization of the major hybrid G4 (Supplementary Figure S23).

Each ^Br^G- and 5′T-modified mutant was subjected to UV melting experiments. No hysteresis effects were observed between heating and cooling curves, implying a thermodynamically controlled transition. In the following, the melting profiles were evaluated by a van’t Hoff analysis in terms of enthalpy Δ*H*°, entropy ΔS°, and Gibb's free energy Δ*G*° for G4 formation at 30°C (data are summarized in Supplementary Tables S4 and S5). Favorable or unfavorable free energy contributions for specific mutations in both a hybrid-type fold (*^6Br^Q*-modified sequences) and a parallel fold (*^5^*′*^T^Q*-modified sequences) are given by changes in Δ*G*° relative to the free energy of the modified parent sequences *^6Br^Q* or *^5^*′*^T^Q*. Notably, except for the *Q-5I* and *Q-5I-11I* double mutant (see below), a good correlation of topology-dependent (de)stabilizing substitution effects in ^Br^G- and 5′T-modified sequences with NMR-derived relative populations for unmodified mutants was found. Consequently, the modified analogs are not only good structural mimics of unmodified quadruplexes but also good surrogates for the analysis of mutation-dependent energy profiles.

**Figure 7.**
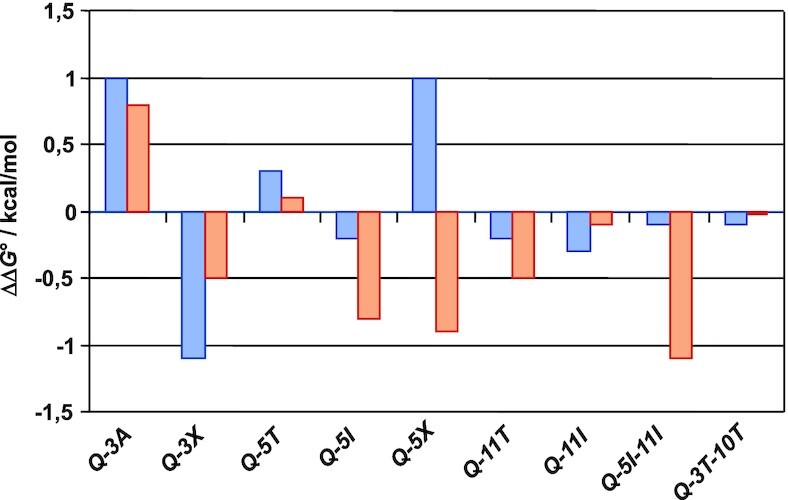
Free energy contribution ΔΔ*G*° of specific mutations upon the formation of a hybrid (blue bars) and a parallel topology (red bars) at 30°C. Uncertainties in the determination of Gibbs free energies averaged over three independent measurements were estimated to be between ±0.1 to ±0.2 kcal/mol (for exemplary van’t Hoff plots see Supplementary Figure S24). No data were extracted for the *Q-11X* mutant due to a more significant structural heterogeneity.

Free energy contributions of the various mutant sequences when either folded into a hybrid or parallel quadruplex are plotted in Figure 7. Apparently, loop substitutions exert moderate but often noticeable effects by up to 1 kcal/mol on the quadruplex stabilities. Position dependent changes can be summarized as follows:

- Replacing C3 by adenosine has a destabilizing effect for both hybrid and parallel G4s but a 3C→3X substitution stabilizes the G4 and in particular the +(lpp) topology. Destabilizing effects for C/T→A substitutions at the first position of a loop have been reported before (14,49). Because this position seems most disordered in both the lateral and the propeller loop of the hybrid and parallel G4, destabilization can be attributed to more unfavorable hydrophobic effects of a solvent-exposed purine compared to a pyrimidine base. By the same reasoning, an abasic residue should exert a stabilizing effect as observed. Corresponding destabilizing effects for a T→A substitution in 1-nt propeller loops have also been well documented in the past (11,50).
- A 5A→5T and especially a 5A→5X substitution destabilizes the hybrid topology but stabilizes the parallel topology in case of *Q-5X*. Here, destabilization only found for the hybrid fold again confirms critical purine stacking on the outer tetrad for the 3-nt lateral loop end position associated with a shift towards a parallel topology upon replacing A by a pyrimidine or abasic nucleotide.
- Substitutions at position 11 but also C→T mutations as in *Q-3T-10T* have only a small impact on stabilities as already suggested by conserved populations of hybrid and parallel species. Apparently, interactions of A11 as indicated in the hybrid fold provide only minor contributions to the G4 stability.
- There is a conspicuous stabilizing effect on the parallel topology upon a 5A→5I substitution in *Q-5I* and also on double mutant *Q-5I-11I*. Such a stabilization with a ΔΔ*G*° of about –1 kcal/mol should give an estimated fivefold increase of the population ratio in favor of the parallel species at 30°C. However, NMR signal intensities point to a conserved major hybrid structure. Apparently, inosine here seems to favorably interact with the adjacent 5′-T overhang of the parallel G4, concealing true effects on the non-modified mutant in this particular case.

Interestingly, there is a pronounced enthalpy-entropy compensation on the substitution-induced free energy effects (see Supplementary Tables S4 and S5). However, corresponding changes in G4 stabilities are mostly governed by enthalpic when compared to smaller entropic contributions in line with previous reports on other loop-dependent changes in stability (11,12).

### Impact of a central 1-nt loop on the global fold

**Figure 8.**
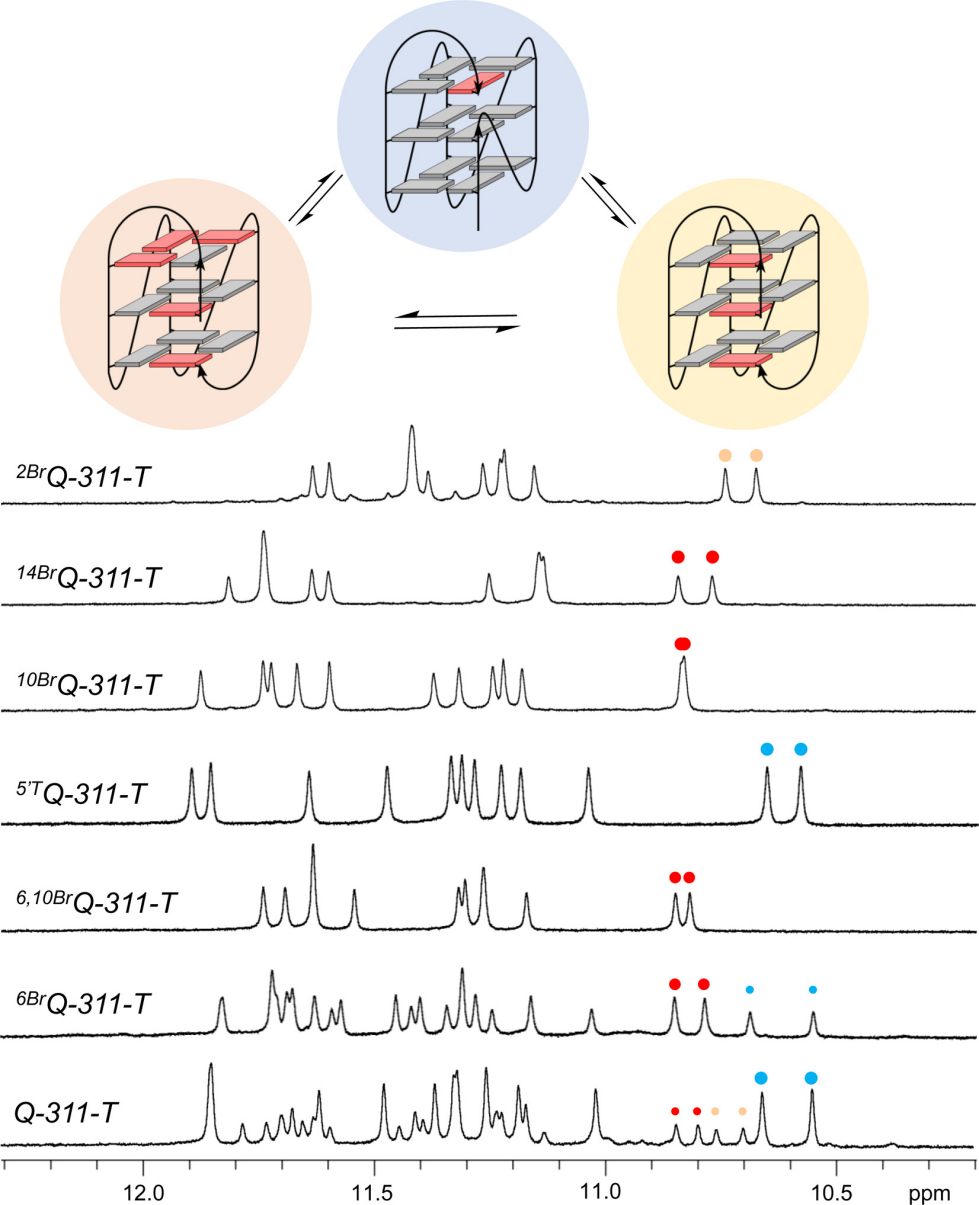
Imino proton spectral region of *Qref* derived quadruplexes with a central 1-nt T-loop. NMR spectra were acquired in 10 mM potassium phosphate buffer, pH 7.0, at 30°C. Blue, red, and yellow dots of varying size mark major and minor high-field shifted resonances of G8 and G20 residues in a parallel topology, a +(lpp) topology with a first *syn*-*syn*-*anti* column, and a +(lpp) topology with a first *syn*–*syn*–*syn* column as shown on top.

Two 1-nt loops or even a single but central 1-nt loop are known from empirical studies to strongly promote all-parallel topologies irrespective of the length and composition of other intervening sequences present (22,23). Apparently, formation of a propeller loop affects formation of neighboring loops in a way not yet understood. The snapback loop G4 architecture allowing for either a stable hybrid or parallel species offers the opportunity to study such an effect in more detail. Consequently, exchanging the second 3-nt loop in the *Qref* sequence by a 1-nt T-loop gives a truncated mutant 5′-GGCTAGGGTGGGTGGGTCAG-3′ termed *Q-311-T* with a 3–1-1 loop length arrangement. Of note, a central 1-nt propeller loop seems identical for both topologies in spanning three tetrad layers across a medium groove. Nevertheless, coexisting with two minor species of similar intensity (see below), a predominant G4 populated by about 60% and identified as a parallel -(ppp) conformer was observed for *Q-311-T* in line with expectations (Figure 8). A major parallel fold is also clearly apparent from its CD signature (Supplementary Figure S25).

There have been reports of forming very stable multimeric quadruplexes with sequences comprising short loops even at low concentration (51). To confirm exclusive formation of monomolecular structures and rule out potential misinterpretations due to aggregation effects, non-denaturing polyacrylamide gel electrophoresis was performed for all G4-forming sequences (Supplementary Figure S26). There was no indication of any noticeable association of the G4s including those with a shortened loop. Apparently, the snapback loop motif inherent to all structures may effectively prevent multimer formation under the present conditions.

A significant population of a parallel species even for the ^Br^G-modified *^6Br^Q-311-T* analog again demonstrates a strong propensity of the sequence with a second central 1-nt loop to adopt an all-parallel topology. A single hybrid-type species was only enforced through introduction of a second ^Br^G substitution at *syn*-position 10 to give *^6,10Br^Q-311-T*, further shifting topological equilibria (Figure 8). In line with a shift towards the parallel species, there is a strongly stabilizing effect for the parallel G4 upon shortening the central 3-nt propeller loop into a 1-nt T-loop. A van’t Hoff analysis of UV melting transitions gives a change in Δ*G*°_30_ of -4.5 kcal/mol when going from parent *^5^*′*^T^Q* to *^5^*′*^T^Q-311-T*, both exclusively folded into a parallel G4 (Table 2). Such a dramatic stabilization is remarkable and results from a significantly more favorable enthalpy overcompensating for a less favorable entropic change.

**Table 2.** Thermodynamic parameters of G4 formation for sequences with 3–3–1 and 3–1–1 loop length arrangements at 30°C^a^

Sequence	*T*_m_ (°C)	Δ*H*° (kcal/mol)^b^	–*T*Δ*S*° (kcal/mol)^c^	Δ*G*°_30_ (kcal/mol)^d^	ΔΔ*G*°_30_ (kcal/mol)^e^
*^5^* ′*^T^Q*^f^	44.1 ± 0.3	–53.7 ± 1.9	51.3 ± 1.7	–2.4 ± 0.1	0*^f^*
*^5^* ′*^T^Q-311-T*^f^	61.5 ± 0.7	–72.9 ± 2.8	65.9 ± 2.7	–6.9 ± 0.2	–4.5 ± 0.3^f^
*^16Br^Q* ^g^	49.3 ± 0.2	–53.2 ± 1.3	50.0 ± 1.2	–3.2 ± 0.1	0^g^
*^14Br^Q-311-T* ^g^	59.2 ± 0.2	–72.3 ± 0.6	65.9 ± 0.6	–6.4 ± 0.1	–3.2 ± 0.2^g^
*^2Br^Q* ^h^	47.7 ± 0.5	–53.9 ± 0.7	50.9 ± 0.7	–3.0 ± 0.1	0^h^
*^2Br^Q-311-T* ^h^	65.5 ± 0.3	–71.4 ± 0.7	63.8 ± 0.6	–7.5 ± 0.1	–4.5 ± 0.2^h^

^a^Average values with standard deviations derived from the analysis of three independent UV melting experiments. ^b^Determined from a van’t Hoff plot.

^c^Δ*S*° = Δ*H*°/*T*_m_.

^d^Δ*G*° = Δ*H*° – *T*Δ*S*°.

^e^Difference in Gibb's free energy Δ*G*°_30_ to the corresponding 3–3–1 quadruplex of the same topology and conformation.

^f^-(ppp) topology.

^g^+(lpp) topology with one *syn*-*syn-anti* and three *syn*-*anti*-*anti* columns.

^h^+(lpp) topology with one all-*syn* and three all-*anti* columns.

Evaluating corresponding effects for a loop shortening in case of a heteropolar stacked hybrid as performed successfully for single residue substitutions through the ^Br^G modification is hampered by a significant amount of an additional parallel fold found for *^6Br^Q-311-T* (see Figure 8). Although imparting high stability, 1-nt propeller loops also impose steric restraints and higher rigidity. Interestingly, a complete tetrad flip with *anti* → *syn* transitions in an all-*anti* parallel G4 was most effectively induced by the incorporation of a *syn*-affine ^Br^G at a position following a 1-nt propeller loop. This was attributed to direct steric clashes of the 8-bromine substituent with the 5′-phosphate oxygen atoms in *anti*-^Br^G but not in *syn*-^Br^G (52). Consequently, a ^Br^G substitution at position 10 or 14 following the 1-nt loops in *Q-311-T* was expected to increasingly shift equilibria towards the hybrid fold. In fact, *^10Br^Q-311-T* but also *^14Br^Q-311-T* features a clean one-component spectrum without any coexisting additional species as observed for mono-substituted *^6Br^Q-311-T* with a bromination site following the 3-nt loop (Figure 8).

To exclude a putative direct impact of the introduced bromo-substituted G on the central loop, the *^14Br^Q-311-T* sequence was selected for a comparative thermodynamic analysis with the corresponding reference sequence *^16Br^Q* featuring a 3-nt central loop. Placed at position 14 or 16, the ^Br^G analog is far removed from the loop of interest and allows for a straightforward comparison of loop length dependent effects. Initially, partial NMR assignments combined with closely matching crosspeak patterns observed for the already characterized *^6Br^Q* hybrid quadruplex unambiguously identified a +(lpp) hybrid to also constitute the favored conformer for the *^14Br^Q-311-T* and *^16Br^Q* sequence (Supplementary Figure S27). Additional minor resonances observed in the *^16Br^Q* imino proton NMR spectral region were shown by DSC measurements to result from some high-melting species and hardly impact thermodynamic data obtained from a van’t Hoff analysis of the lower-melting quadruplex-to-single strand transition (Supplementary Figure S28). With a ΔΔ*G*°_30_ of –3.2 kcal/mol there is also a significant stabilization of the hybrid topology upon shortening the central loop (Table 2). However, in line with empirical observations on the effect of 1-nt loops on G4 folding, the gain in stability of the parallel topology is noticeably higher by more than 1 kcal/mol when compared to the hybrid structure with its heteropolar stacking.

While the thermodynamic data above constitute an energetics-based explanation for the propensity of 1-nt loops to fold into a parallel topology, the structural basis of enforcing other loops to also adopt a propeller-type conformation remains elusive. As a matter of fact, a propeller loop will always link the two outer tetrads across a medium groove irrespective of the global topology. Also, there should be no direct interactions of the nucleobase within the central 1-nt loop with other residues of the G-core. This was demonstrated by substituting the 1-nt T-loop residue in *^10Br^Q-311-T* with an abasic residue X to give *^10Br^Q-311-X*. In addition to mostly identical thermodynamic profiles obtained from UV melting transitions of the two sequences, a clean hybrid-type spectrum with an imino proton spectral region closely matching imino proton chemical shifts of *^10Br^Q-311-T* was observed for *^10Br^Q-311-X*, corroborating the lack of any noticeable interactions emanating from the 1-nt loop (Supplementary Figure S29 and Supplementary Table S6).

Recently, hybrid-type +(lpp) and –(ppl) topologies with two propeller loops termed hybrid-1R and hybrid-2R have been reported for the first time (Supplementary Figure S30). Formation of the corresponding canonical quadruplexes with four non-interrupted G_3_-columns was enforced by additional ^Br^G modifications and further supported by Watson-Crick base pairing of overhang and loop residues to yield coaxially stacked duplex extensions (53,54). Given their design comprising two 1-nt propeller loops, their resistance against folding into the target hybrid G4 with a single lateral loop can be attributed to the combined effect of two 1-nt loops strongly favoring a parallel topology irrespective of the length of the third loop. It is conspicuous, however, that in both cases a rather unusual (3 + 1) hybrid conformer featuring tetrads of the same polarity with an antiparallel *syn–syn–syn* column and three parallel all-*anti* columns was the major species. A coexisting minor conformer for the +(lpp) G4, termed hybrid-1R’, was demonstrated to comprise a *syn*–*syn*–*anti* and three *syn*–*anti*–*anti* columns (54). On the other hand, it is a corresponding hybrid-1R’ G4 with the same pattern of G-core glycosidic conformations that predominates for the present +(lpp) hybrid-type mutants with their central 3-nt propeller loop. In fact, based on unfavorable stacking interactions of *syn*-*syn* steps, the latter should be preferred over the hybrid-1R conformer with its all-*syn* column (55,56). Notably, –(pll) and –(llp) topologies (hybrid-1 and hybrid-2) formed by telomeric sequences in a K^+^ buffer likewise feature three *syn*-*anti*-*anti* and a single *syn*-*syn*-*anti* column with a heteropolar G-tetrad stacking (Supplementary Figure S30).

**Figure 9.**
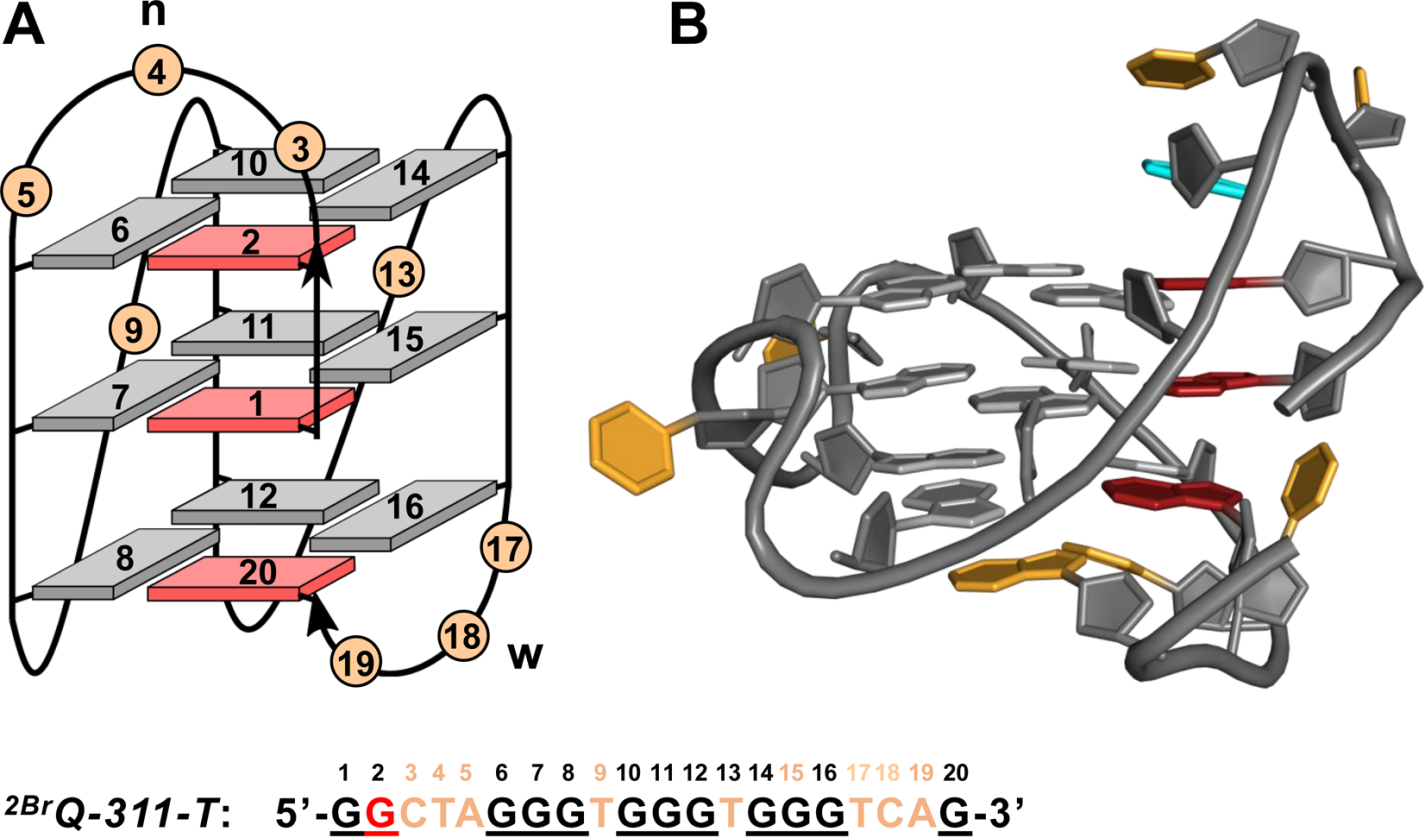
(**A**) Topology of the *^2Br^Q-311-T* quadruplex with a 3–1–1 loop arrangement; *syn*- and *anti*-G residues are colored red and grey, n and w denote narrow and wide groove, respectively. The sequence of *^2Br^Q-311-T* with residue numbering is shown at the bottom; ^Br^G residue in red, intervening sequences in orange, and Gs participating in tetrad formation underlined. (**B**) Three-dimensional structure of *^2Br^Q-311-T*; color scheme as in (A) but with A5 colored in cyan.

To also enforce a hybrid-1R conformer, the ^Br^G analog was introduced at position 2 of the *Q-311-T* sequence to match a *syn*-position in the snapback loop G4 in case of an all-*syn* first G-column. The *^2Br^Q-311-T* imino proton spectral region shows a single three-layered G4 species with only very minor additional resonances (Figure 8). In the following, a detailed NMR spectral analysis with complete resonance assignments was performed on the *^2Br^Q-311-T* G4 (for spectral assignment strategies, spectra, and chemical shifts see Supplementary Figure S31–S33 and Supplementary Table S7 in the Supporting Information). In fact, based on the NMR data, formation of a hybrid-1R conformer with a lateral loop followed by two propeller and a snapback loop and with a G-core consisting of a first interrupted all-*syn* tract (G20–G1–G2) and three all-*anti* G-columns (G6–G7–G8, G10–G11–G12 and G14–G15–G16) was unambiguously confirmed (Figure 9A). For a three-dimensional structure determination, restrained molecular dynamics calculations were performed for *^2Br^Q-311-T* in explicit water. With a total rmsd of 1.5 Å and a G-core rmsd of 0.7 Å, the calculated structures are well defined (Supplementary Table S8 and Supplementary Figure S34A). Comprising an all-*syn* column, the G4 exclusively features homopolar tetrad stackings as indicated by its CD signature (Figures 9 and Supplementary Figure S25). The six-membered ring of the A5 purine within the first lateral loop stacks onto the six-membered pyrimidine ring of G2 (Supplementary Figure S34B). This small displacement when compared to the *^6Br^Q* quadruplex can be attributed to the upper tetrad polarity reversal and possibly also to the absence of another interaction with the loop residue from the second 1-nt propeller loop. Both 1-nt propeller loops are either located in the G4 groove or exposed to solvent as also indicated by the lack of inter-residual contacts.

With the availability of corresponding reference spectra, the two minor species for *Q-311-T* were unambiguously identified as +(lpp) hybrid-1R’ and hybrid-1R G4s with a first *syn*–*syn*–*anti* column and a first all-*syn* column through a match of typical crosspeak patterns (Supplementary Figure S35). Likewise, although only poorly populated, a minor third species observable in the parent *Qref* G4 spectra could finally also traced to the all-*syn* hybrid-1R topology with three tetrads of the same polarity (Supplementary Figure S36) and this assignment should also apply to additional very low-populated species noticeable in various mutant spectra (see Figure 6).

With the completed assignment of three coexisting G4 conformers for the *Qref* and *Q-311-T* sequences, molar ratios were determined based on intensities of non-exchangeable T methyl resonances (Supplementary Figures S35 and S36). For *Qref*, molar ratios were determined to be 27% for the parallel topology, 11% for the hybrid-1R, and 62% for the major hybrid-1R’ conformer. For the *Q-311-T* sequence with a second 1-nt loop, populations of parallel and hybrid-1R topologies increased by a factor of about two at the expense of the hybrid-1R’ conformer, yielding populations of a major parallel, hybrid-1R, and hybrid-1R’ species of 54%, 19% and 27%, respectively. Apparently, in addition to supporting a parallel G4, shortening the 3-nt central loop to a 1-nt loop strongly promotes the all-*syn* conformer with respect to the *syn*-*syn*-*anti* conformer.

For a more detailed thermodynamic analysis of the impact of loop shortening on the hybrid-1R formation, UV melting transitions were again analyzed in detail for the pair of loop length related G4s with a hybrid-1R fold, namely*^2Br^Q-311-T* and *^2Br^Q*. A corresponding hybrid-1R fold for *^2Br^Q* was again demonstrated through partial NMR assignments and the observation of closely similar crosspeak patterns to *^2Br^Q-311T* (Supplementary Figure S37). Differences in free energies of G4 formation amount to a significant ΔΔ*G*°_30_ of –4.5 kcal/mol in favor of *^2Br^Q-311-T* with a single-nucleotide central loop (Table 2). Interestingly, such a stabilization is identical to the corresponding loop length mediated stabilization for a parallel topology and exceeds the stabilization for a hybrid-1R’ topology by more than –1 kcal/mol.

Taken together, these results suggest that the propensity of a 1-nt propeller loop in also promoting a propeller-type conformation of other loops seems a direct consequence of its preference in linking two outer G-tetrads with the same tetrad polarity, i.e. with two anchoring G residues of the same glycosidic conformation. Such a propensity to drive folding into a parallel structure does not depend on specific interactions between G4 residues but is expected to be based on geometric effects. Thus, accompanied by conformational changes in the sugar-phosphate backbone, guanine bases must rearrange when trying to optimize hydrogen bond interactions within the tetrad upon *anti*→*syn* transitions. Interestingly, explicit solvent molecular dynamics simulations on single-loop G4s also reported a preference for the 1-nt propeller loop traversing three tetrad layers when linking two *anti*-Gs over linking an *anti*-G with a *syn*-G residue (18). In the same study, a significant preference of a three-nucleotide loop to form a lateral over a propeller loop was reported. However, each 1-nt propeller loop with its propensity to drive folding into a parallel structure will oppose formation of a 3-nt lateral loop, with two 1-nt loops being more efficient than a single 1-nt loop but also with putative loop position dependent effects (21).

Following the considerations above, the parallel and hybrid-1R topology are expected to be equally promoted by 1-nt loops. However, because an all-*syn* column is considerably disfavored due to poor stacking interactions along *syn*-*syn* steps, an all-*anti* parallel topology will usually predominate unless appropriate modifications are introduced to enforce the hybrid-type G4. Likewise, an energetic penalty associated with an all-*syn* column may favor a hybrid-1R’ over a hybrid-1R conformer to still result in a slightly higher population of the former in the *Q-311-T* G4.

It seems counter-intuitive that a *syn*-affine ^Br^G analog will be most effective in adopting a *syn* conformation associated with a complete tetrad flip when incorporated at a tetrad position directly following a 1-nt propeller loop. However, such a conformational transition results from specific bromine steric clashes in case of ^Br^G in an *anti*-conformation, outweighing a more favorable homopolarity of bridged outer tetrads. These effects emphasize the often subtle and interrelated loop interactions within a G-quadruplex structure and serve as a reminder that it still remains a challenge to disentangle these rather delicate energetic contributions and to predict their overall impact on putative G4 folds.

### Implications for the folding and stability of G-rich sequences

By using a rigorous approach in combining detailed sequence-dependent structural and thermodynamic information, the present studies not only extend some empirical rules on the impact of loops in G4 folding, but also provide for an in-depth molecular understanding of findings put forward in the past. Although results have been derived from a particular G4 architecture lacking overhang sequences to focus on inherent G4 interactions, important generalizations emerge. Thus, whereas first loop residues tend to be rather flexible and mostly exposed to solvent with no long-lived specific interactions, 3′-terminal loop residues are often engaged in stacking interactions with an outer G-tetrad. These can provide for a significant stabilization of lateral loops bridging a narrow groove but may also form in longer propeller loops with ≥3 residues. For the latter, stacking of the last residue onto an external tetrad is often promoted by additional base-base interactions with a loop or overhang residue to form a capping structure. This seems to be a more general structural arrangement and can be found in quadruplexes of promoter sequences such as c*-myc* or *PARP1* (48,57). In general, stacking interactions will benefit from larger ring systems, favoring purine over pyrimidine bases. In contrast, if exposed to solvent, a smaller and less extended hydrophobic surface area of a pyrimidine base is expected to be less destabilizing based on hydrophobic effects when located at the loop 5′-end. These effects are reflected in the well-known empirical findings that adenine is destabilizing at first loop positions or in 1-nt propeller loops but mostly stabilizing when positioned at the last loop position. Such contributions may also be biologically relevant in selecting for G4 loop isomers from oncogene promoter sequences with short adenine-deficient propeller loops. Also, favorable 5′-TTA-3′ intervening tracts in the human telomeric sequence may result from natural selection. On the other hand, such a discrimination between first and last loop residue does not necessarily apply to lateral loops bridging a wide groove. Progressing along the right-handed G-tract helicity (43), the smooth transition to the loop domain in this case allows the first residue to be easily stacked on a tetrad, often forming a capping base pair with the last base of the loop as observed for the lateral snapback loop in the *^6Br^Q* and *^5’T^Q* G4s (Supplementary Figure S13).

From a structural point of view, a prominent A·A cap on the upper tetrad formed by end residues of the 3-nt lateral loop bridging the narrow groove and a following 3-nt propeller loop seems an obvious stabilizing element in the present hybrid-type structure. However, point mutations and a more detailed thermodynamic analysis indicates that it is only the stacked 3′-terminal adenine of the lateral loop that promotes a hybrid over a parallel fold whereas the stacked 3′-terminal adenine of the propeller loop hardly exerts any impact on topological equilibria and thermodynamic stabilities. Obviously, structural information on a single competing species can be misleading when it comes to stability effects and only when complemented by thermodynamic profiles for all competing structures allows for the evaluation of critical interactions and ultimately the prediction of most favored folding pathways.

Based on purely geometric restraints it comes as no surprise that a 1-nt intervening sequence will form a propeller-type loop linking two adjacent parallel G-tracts. On the other hand, the observed propensity of 1-nt propeller loops to also enforce a propeller-type conformation on an additional longer loop and to therefore promote an all-parallel topology has been elusive. In the absence of any observable interactions of the single-nucleotide loop residue, kinetic effects may be operative. However, the present studies do not support a kinetic control of folding pathways. Whereas there are no significant hysteresis effects upon melting for each of the topologies examined, changing a second 3-nt to a 1-nt propeller loop does in fact result in a much higher, mostly enthalpy-driven, thermodynamic stabilization of three-layered G4s with exclusively homopolar G-tetrads when compared to a typical hybrid fold with outer G-tetrads of opposite polarity. To put it another way, a 1-nt propeller loop will preferably link two *anti*-G residues in opposite outer tetrads and such a preference will strongly promote formation of all-*anti* parallel quadruplexes in most cases. However, it is reasonable to assume that 1-nt propeller loops will also favor two anchoring *syn*-Gs in outer tetrads of the same polarity, yet corresponding conformers have never been observed in a three-layered G4 until now.

An observed resistance of sequences to folding into +(lpp) and –(ppl) topologies with two 1-nt propeller loops is a direct consequence of the added propensity for those loops to link outer tetrads of the same polarity. The same applies to folding into a –pd+p topology expected to be perfectly feasible but not experimentally verified to date. Thus, in the presence of single-nucleotide loops a longer third intervening sequence will likewise be forced into a propeller loop to conserve homopolarity as found for several natural promoter sequences like *VEGF*, *HIF-1α*, *c-kit* or *BCL-2* (58–61). On the other hand, elongating 1-nt to 2-nt propeller loops will result in a delicate balance between a release of the constraints enforcing an all-parallel topology and an enhanced propensity of the propeller loop to rearrange into an easily accessible 2-nt lateral loop.

These loop-dependent equilibria also direct attention to the formation of an alternative homopolar hybrid-type quadruplex with an all-*syn* G-tract, likewise supported by 1-nt propeller loops but unnoticed due to the lack of corresponding high-resolution data until now. Clearly, comprising two unfavorable *syn*-*syn* steps, such a G4 conformer will be disadvantaged compared to a parallel structure with an all-*anti* G-core. However, a (3 + 1) hybrid fold may be supported by Watson-Crick base pairing of complementary bases in its lateral loop (see also Supplementary Figure S30). In view of reports on the frequent occurrence of duplex stem-loop containing quadruplex sequences within the human genome (62), quadruplex-duplex hybrids with coaxially stacked duplex extensions and a single antiparallel all-*syn* column are conceivable to not only result from a rational sequence design for specific applications, e.g. as aptamers, but to also form more frequently within genomic sequences.

## CONCLUSIONS

The artificial design of a non-canonical G-quadruplex with a snapback loop at its 3′-terminus offers the possibility to narrow putative folding pathways for spectral simplifications. It also enables better control on a topological selection while hampering G4 association through the stacking of outer tetrads due to steric hindrance by its snapback loop. With a combination of sequence mutations, high-resolution structural analyses, and thermodynamic profiling, loop residue interactions and their impact on the G4 fold can be characterized in detail to reveal relationships between loop length and loop composition with stabilities and conformational preferences of G4 structures. It also provides for a better structure-based understanding of many empirical findings for a more effective G4 design and sequence-based topological prediction.

The ability of rather weak interactions to drive folding into a particular topology is based on often small differences in stability among competing G4 structures. Even with a growing understanding of loop residue interactions, a reliable prediction of sequence dependent G4 topologies within a biological context where molecular crowding conditions and additional interactions with proteins may easily redirect folding pathways seems challenging. However, the engineering of defined G4 structures under controlled conditions for various medicinal and technological applications is expected to strongly benefit from a detailed understanding of intrinsic forces that stabilize quadruplexes. The availability of an increasing number of high-resolution G4 structures and their detailed thermodynamic profiling is expected to allow for an ever-growing understanding of sequence-specific intrinsic interactions guiding G4 folding.

## DATA AVAILABILITY

The atomic coordinates and chemical shifts for *^6Br^Q* (PDB 7ZEK; BMRB 34721), *^5^*′*^T^Q* (PDB 7ZEM; BMRB 34722) and *^2Br^Q-311-T* (PDB 7ZEO; BMRB 34723) have been deposited in the Protein Data Bank.

## Supplementary Material

gkac549_Supplemental_File
