# Supplementary material for: Guiding the folding of G-quadruplexes through loop residue interactions
Source: Nucleic Acids Res. 2022 Jun 27;50(12):7161–75. doi: 10.1093/nar/gkac549 (PMC9262619; doi:10.1093/nar/gkac549)
Supplement: gkac549_Supplemental_File [file gkac549_supplemental_file.pdf]

Supplementary Information
for

Guiding the Folding of G-Quadruplexes Through Loop Residue Interactions

Jagannath Jana, Yoanes Maria Vianney, Nina Schröder and Klaus Weisz*
Institute of Biochemistry, Universität Greifswald, 17489 Greifswald, Germany

Contents

NMR spectral analysis and resonance assignments for ^{6Br}*Q* S2

NMR spectral analysis of *Qref* S8

NMR spectral analysis and resonance assignments for $^{5'T}$*Q* S10

Structural statistics and NMR-derived structures of ^{6Br}*Q* and $^{5'T}$*Q* S15

NMR spectral analysis for *Q-5T* and *Q-11T* S18

CD spectra of all sequences with a 331 loop length arrangement S22

Imino proton NMR spectral region of all modified sequences with a 331 loop length arrangement S25

Thermodynamic analysis for modified sequences with a 331 loop length arrangement S27

CD spectra of sequences with a 311 loop length arrangement S29

Non-denaturing polyacrylamide gel electrophoresis of all quadruplexes S30

Comparative ^{1}H-^{13}C HSQC spectra of ^{6Br}*Q*, ^{16Br}*Q*, and ^{14Br}*Q-311-T* S31

Melting analysis of major and minor ^{16Br}*Q* quadruplex S32

Thermodynamics of ^{10Br}*Q-311-T* and ^{10Br}*Q-311-X* quadruplex formation S33

Schematic representation of (3+1) hybrid topologies S34

NMR spectral analysis and resonance assignments for ^{2Br}*Q-311-T* S35

Structural statistics and NMR-derived structure of ^{2Br}*Q-311-T* S40

Determination of coexisting G4 species with relative populations for *Q-311-T* and *Qref* S42

Comparative ^{1}H-^{13}C HSQC spectra of ^{2Br}*Q* and ^{2Br}*Q-311-T* S44

NMR spectral analysis and resonance assignments for *^{6Br}Q*

The *^{6Br}Q* sequence exhibits 12 well-resolved imino proton resonances, indicative of a single G-quadruplex conformer with three G-tetrad layers. Guanine imino and H8 protons were unambiguously assigned without specific isotope labeling through inter-residual NOE contacts as well as through ^{1}H–^{13}C HSQC and ^{1}H–^{13}C HMBC experiments at natural abundance. Four *syn*-guanosines were assigned based on their strong H8−H1′ cross-peak intensities in 2D NOESY spectra (increasingly pronounced at shorter mixing times) and corroborated by their *syn*-typical chemical shift in a ^{1}H-^{13}C HSQC spectrum (Figures S1B and S2**)**. Another yet non-identified *syn*-G must come from the *syn*-favoring 8-bromoguanosine analog ^{Br}G at position 6, yielding a total of five *syn*-G residues at positions G1, G6, G12, G16, and G22 as well as seven G-core residues with an *anti*-conformation in the G-quadruplex structure. Likewise, H6/H8−H1' and H6/H8-H3' spectral regions of 2D NOESY spectra acquired with longer mixing times (300 ms) showed a rectangular pattern of intra-nucleotide and sequential crosspeaks for G1–G2, G12–G13, and G16– G17, typical for *syn-anti* steps (Figure S1B). Continuous base-sugar NOE walks only interrupted by the 3-nt and 1-nt propeller loop allowed for additional sequential assignments also including lateral and snapback loop residues. Thus, spectral analysis of non-exchangeable protons suggests formation of a three-layered G-quadruplex composed of three *syn*−*anti*−*anti* columns (G6-G7-G8, G12-G13-G14, and G16-G17-G18, as well as one broken *syn*−*syn*−*anti* column G22-G1-G2.

Following assignments of non-labile protons, guanine imino protons were unambiguously assigned based on their intra-residue correlations to H8 in ^{1}H-^{13}C HMBC spectra (Figure S3). A strong NOE crosspeak observed between G1 H1 and G22 H1 indicates the proximity of 5'- and 3'-terminal G residues within the first broken G-column (Figure S1C). The determination of tetrad polarities was enabled by characteristic H8-H1 intra-tetrad NOE contacts (Figure S1D). Hoogsteen hydrogen bonds within the three G-quartets run along G2-G16-G12-G6, G1-G7-G13-G17, and G22-G8-G14-G18 with one homopolar and one heteropolar tetrad stacking in line with the CD spectral signature typical of a (3+1) hybrid structure (see Figure 3 of the main manuscript). Finally, additional confirmation of the G-quadruplex fold comes from H_2O-D_2O exchange experiments, demonstrating increased exchange rates for imino protons located within the 5'- and 3'-outer tetrads when compared to central tetrad iminos (Figure S4).

Stereospecific assignments for H2'/H2" sugar protons were accomplished by analyzing NOESY experiments at short mixing times to allow discrimination of the different H1'-H2' and H1'-H2" NOE crosspeak intensities. Sugar puckers were evaluated through a comparison of H1'-H2' and H1'-H2" DQF-COSY crosspeaks, making use of cancellation effects of DQF-COSY anti-phase crosspeak components in case of smaller coupling constants (Figure S5).

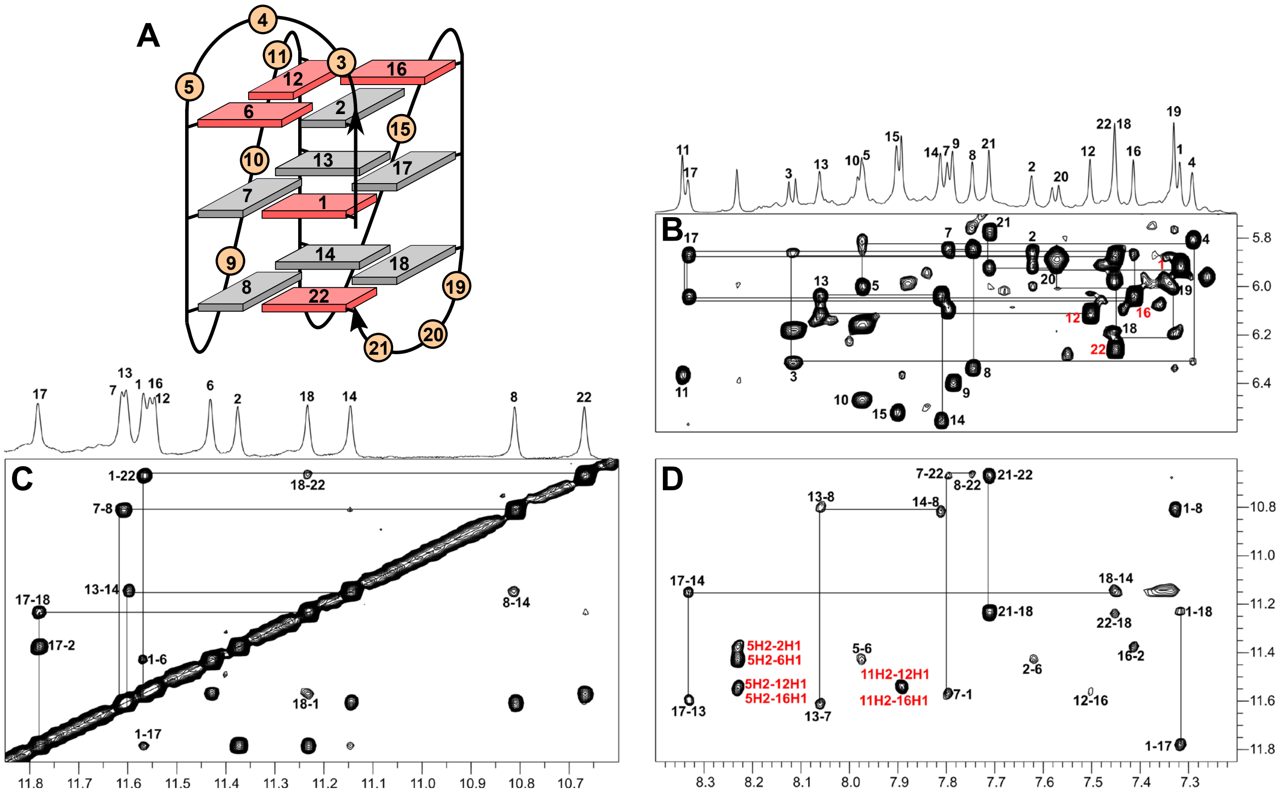

Figure S1. Topology and 2D NOESY spectral regions of *6BrQ* in 10 mM K+ buffer, pH 7.0 (30 °C, mixing time 300 ms). (A) Schematic representation with numbered residues of a (3+1) hybrid-type G-quadruplex with a (+lpp) topology and snapback loop adopted by *6BrQ*; *anti*- and *syn*-guanosines of the G-core are colored grey and red, respectively. (B) H6/H8(ω2)-H1'(ω1) 2D NOE spectral region tracing continuous intra-nucleotide and sequential connectivities; intra-nucleotide crosspeaks of *syn*-guanosines are labelled in red. (C) H1(ω2)-H1(ω1) crosspeaks with sequential contacts traced along the G tracts. (D) H8/H2(ω2)-H1(ω1) NOE contacts with typical intra-tetrad GH8-GH1 connectivities; connectivities of A5H2 and A11H2 with G imino protons of the upper tetrad (labelled in red) indicate stacking of 3'-terminal loop residues A5 and A11 onto the tetrad.

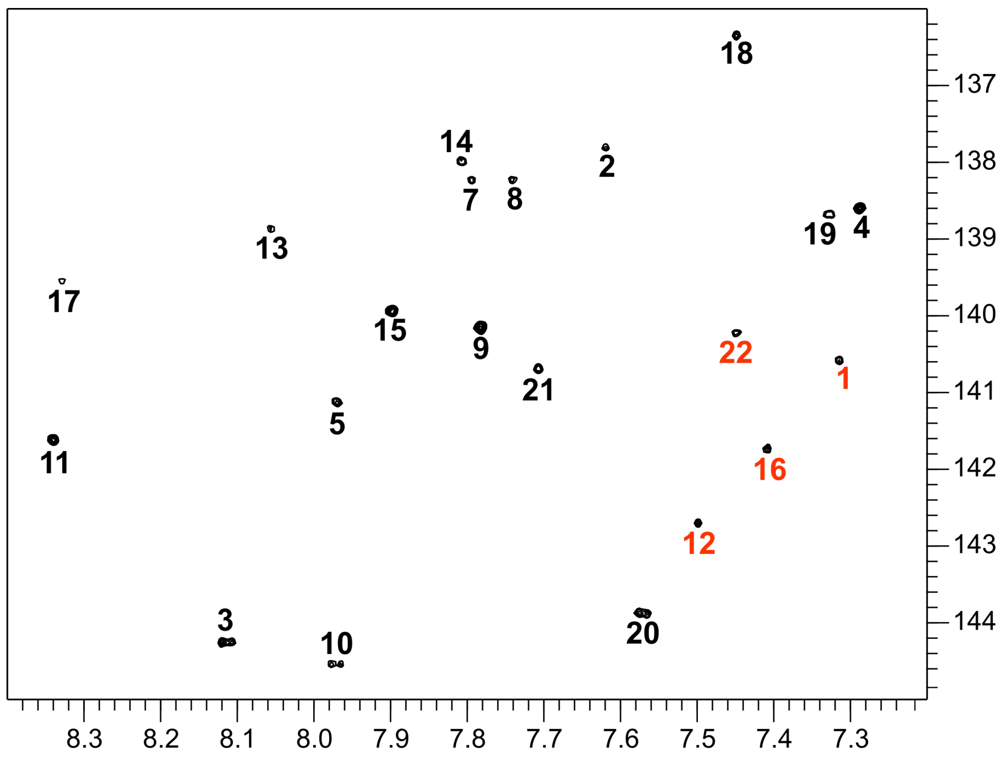

Figure S2. ^{1}H-^{13}C HSQC spectrum of ^{6Br}Q acquired at 30 °C in 10 mM K^{+} buffer, pH 7.0, showing H8/H6–C8/C6 correlations. Crosspeaks of *syn*-guanosines are labelled in red.

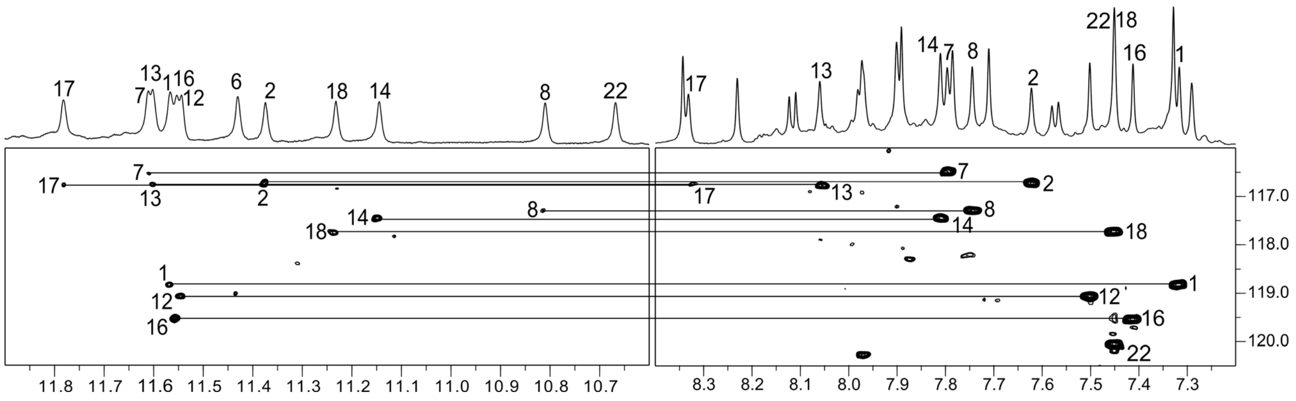

Figure S3. ^{1}H-^{13}C HMBC spectrum of *^{6Br}Q* at 30 °C in 10 mM K^+ buffer, pH 7.0, showing through-bond correlations of guanine H1(ω_2) and H8(ω_2) protons via long-range couplings to $^{13}C5$ (ω_1) at natural abundance.

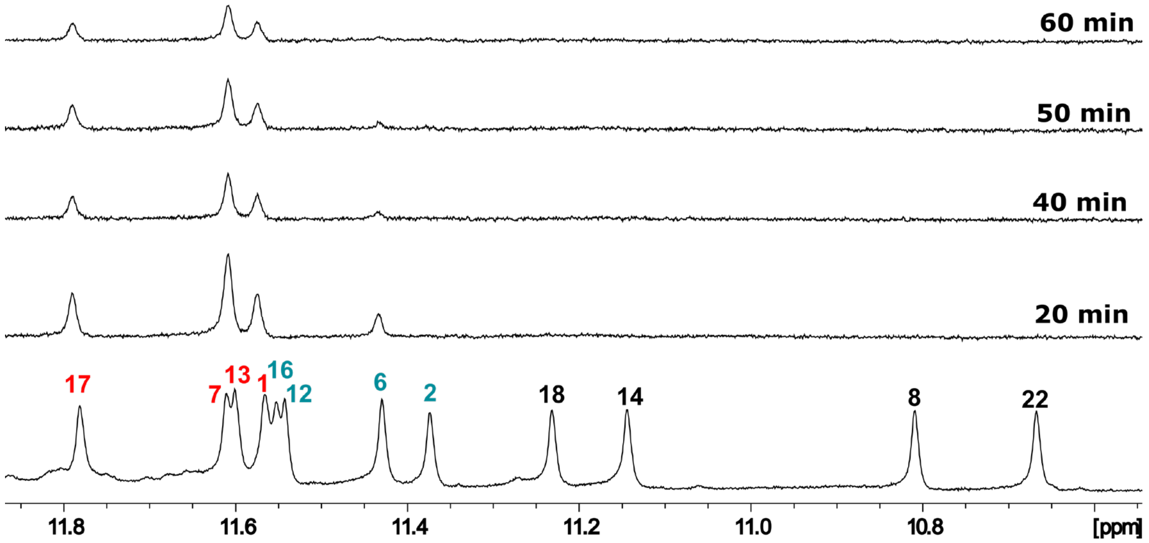

Figure S4. H_2O-D_2O exchange experiments. Imino proton spectral region of *^{6Br}Q* acquired in 10 mM K^+ buffer with 90% H_2O/10% D_2O at 30 °C (bottom) and at increasing time intervals after drying and redissolving the oligonucleotide in 100% D_2O (top). Imino protons of residues located in the central tetrad and, albeit to a smaller extent, of G6 are protected from fast solvent exchange. Numbers of residues in the lower, central, and upper tetrad are labeled in black, red, and blue, respectively.

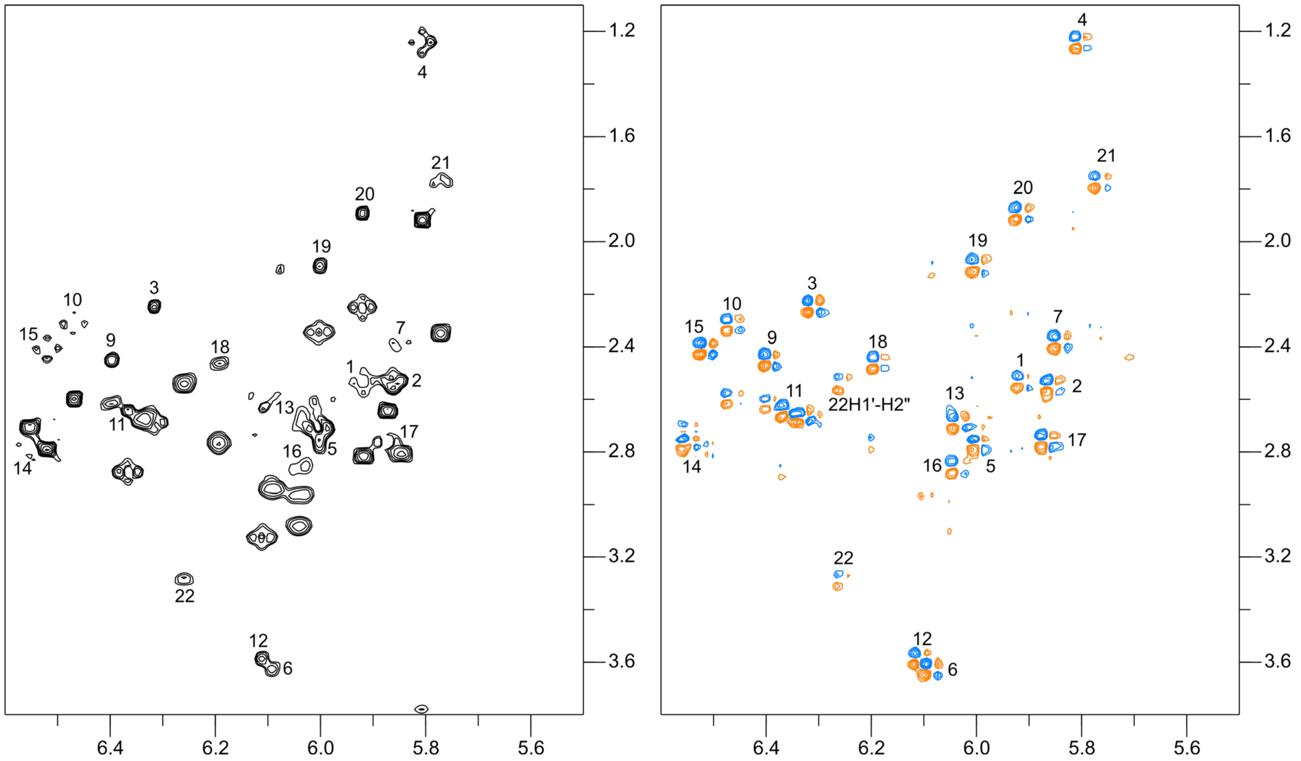

Figure S5. Sugar pucker analysis of *[6Br]Q*. (Left) Stereospecific assignments of H2'/H2" with H1'(ω_2)-H2'/H2"(ω_1) NOESY spectral region at short mixing time (80 ms); crosspeak intensities allow discrimination between H2' and H2". (Right) DQF-COSY spectral region showing H1'(ω_2)-H2'/H2"(ω_1) crosspeaks; *north*- and *south*-type sugar puckers are associated with different scalar couplings and thus different crosspeak patterns of in-phase and anti-phase components.

Table S1. ^{1}H and ^{13}C chemical shifts δ of *^{6Br}Q*.[a]

δ (ppm)	H8/H6	H1/H3	H1’	H2’/H2”	H3’	H5/H2/Me	C8/C6	C2
G1	7.32	11.57	5.92	2.54/2.82	4.98	-	140.59	-
G2	7.62	11.37	5.86	2.54/2.55	5.13	-	137.81	-
C3	8.11	-	6.31	2.25/2.69	4.90	6.18	144.28	-
T4	7.29	n.d.	5.80	1.24/1.92	4.70	1.71	138.62	-
A5	7.97	-	6.00	2.77/2.72	5.00	8.23	141.15	156.16
^{Br}G6	-	11.43	6.09	3.63/2.95	4.95	-	n.d.	-
G7	7.80	11.61	5.85	2.38/2.81	4.92	-	138.23	-
G8	7.74	10.82	6.34	2.66/2.67	5.03	-	138.23	-
T9	7.78	n.d.	6.40	2.45/2.62	4.83	1.96	140.16	-
C10	7.97	-	6.47	2.31/2.60	4.97	6.16	144.52	-
A11	8.34	-	6.36	2.65/2.88	5.01	7.89	141.61	155.01
G12	7.50	11.54	6.11	3.59/3.13	5.03	-	142.71	-
G13	8.06	11.60	6.04	2.69/2.97	5.12	-	138.88	-
G14	7.81	11.15	6.55	2.77/2.70	5.17	-	137.99	-
T15	7.90	n.d.	6.52	2.41/2.79	5.13	2.00	139.97	-
G16	7.41	11.56	6.04	2.86/3.08	5.12	-	141.73	-
G17	8.33	11.78	5.87	2.76/2.64	5.12	-	139.55	-
G18	7.45	11.23	6.19	2.46/2.77	4.89	-	136.35	-
T19	7.33	n.d.	6.00	2.10/2.34	4.68	1.70	138.68	-
C20	7.57	-	5.92	1.89/2.25	4.56	5.88	143.89	-
A21	7.71	-	5.77	1.78/2.35	4.69	7.33	140.70	153.10
G22	7.45	10.67	6.26	3.29/2.54	4.87	-	140.22	-

[a]At 30 ºC in 10 mM potassium phosphate buffer, pH 7.0.

NMR spectral analysis of *Qref*

Similar NOE crosspeak patterns of *Qref* and ^{6Br}Q in the H6/H8-H1' NOESY spectral region and in the H8/H6–C8/C6 region of ^{1}H-^{13}C HSQC spectra clearly suggest the formation of a corresponding +(lpp) hybrid-type G-quadruplex with snapback loop as major species formed by unmodified *Qref* (Figures S6 and S7).

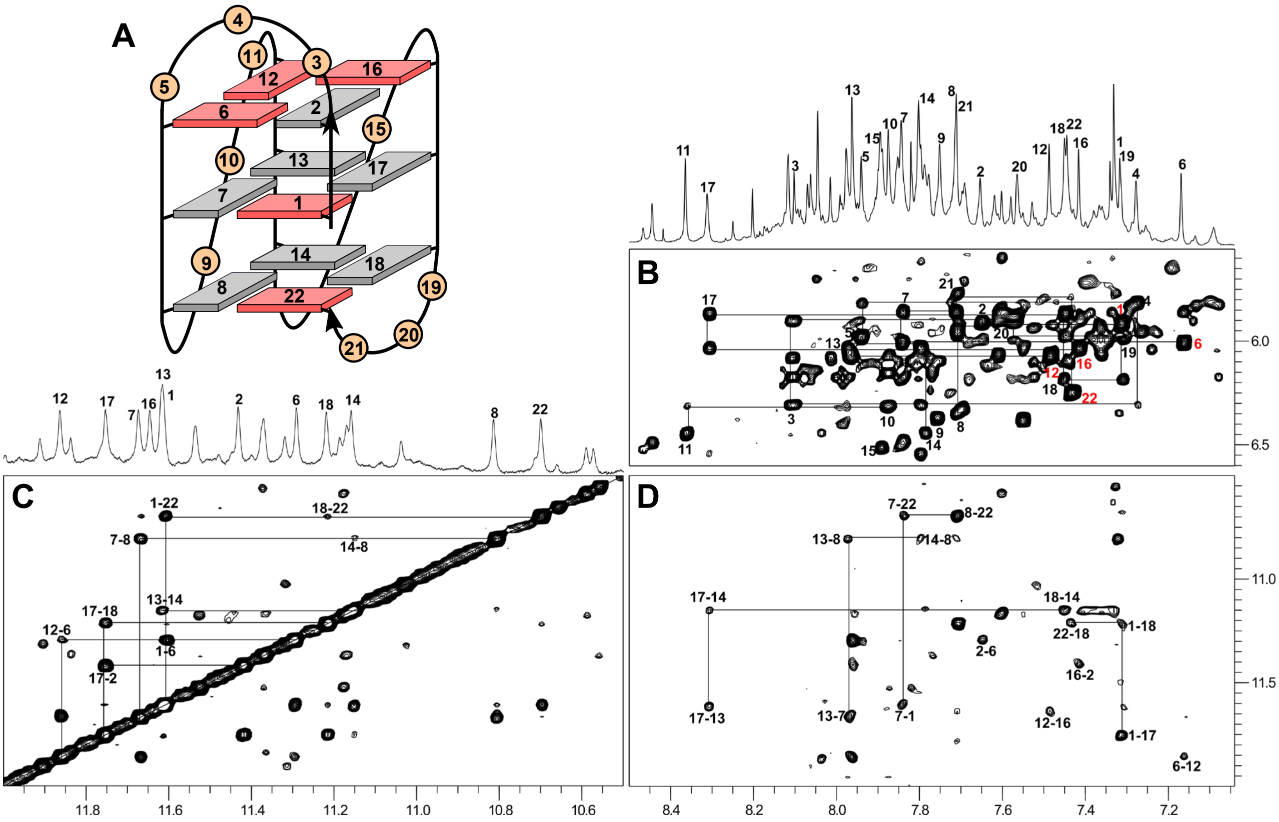

Figure S6. Topology and 2D NOESY spectral regions of *Qref* in 10 mM K^{+} buffer, pH 7.0 (30 °C, mixing time 300 ms). (A) Schematic representation with numbered residues of a (3+1) hybrid-type G-quadruplex with a (+lpp) topology and snapback loop adopted by *Qref*; *anti*- and *syn*-guanosines of the G-core are colored grey and red, respectively. (B) H6/H8(ω_2)-H1'(ω_1) 2D NOE spectral region tracing continuous intra-nucleotide and sequential connectivities; intra-nucleotide crosspeaks of *syn*-guanosines are labelled in red. (C) H1(ω_2)-H1(ω_1) crosspeaks with sequential contacts traced along the G tracts. (D) H8(ω_2)-H1(ω_1) NOE contacts with typical intra-tetrad GH8-GH1 connectivities.

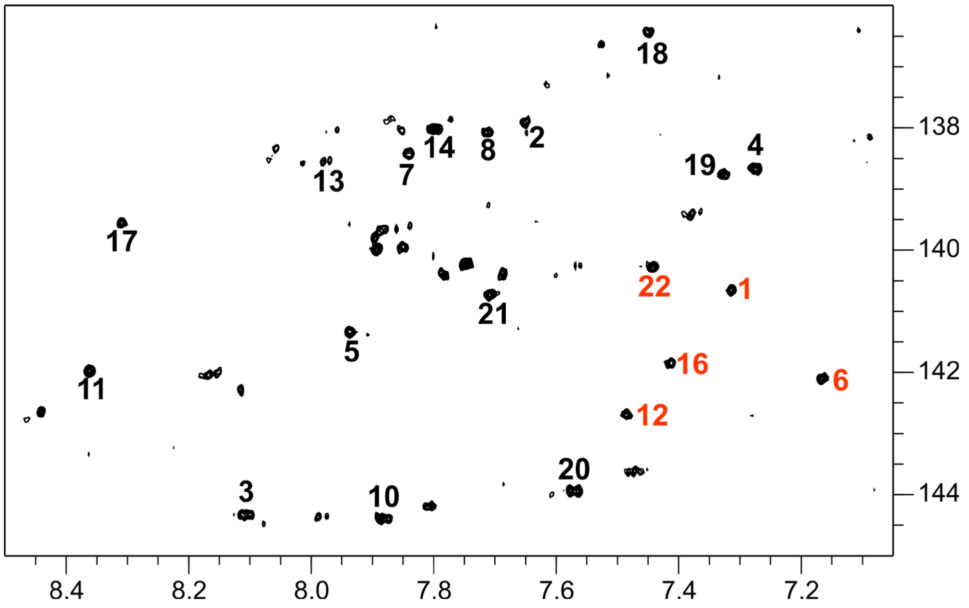

Figure S7. ^{1}H-^{13}C HSQC spectrum of *Qref* acquired at 30 °C in 10 mM K^{+} buffer, pH 7.0, showing H8/H6–C8/C6 correlations. Crosspeaks of *syn*-guanosines are labelled in red.

NMR spectral analysis and resonance assignments for *$^{5'T}Q$*

Upon the addition of a 5'-thymidine to *Qref*, a single three-layered quadruplex with a set of 12 G imino proton signals can be observed (Figure S8). The G-core is comprised of a single *syn*- and eleven *anti*-guanosines as suggested by H6/H8-sugar NOE contacts, NOESY crosspeak intensities, and C8 chemical shifts as observed in a ^{1}H-^{13}C HSQC spectrum (Figures S8B and S9). The G-quadruplex has a parallel topology with a vacant site in the first G-tract that is filled by G22 through a lateral snapback loop composed of residues 19-21. An uninterrupted NOE walk can be followed from residue G16 to the 3'-terminal G22, identifying the fourth G-column. The first G-column can be identified through a contact to the T(-1) 5'-overhang and is formed by *anti*-G1 followed by *anti*-G2 and *syn*-G22. The second G-column starting at G6 is discriminated from the third G-tract by sequential contacts between preceding A5 to G6. G-columns G6-G7-G8, G12-G13-G14, and G16-G17-G18 exclusively comprise *anti*-*anti*-*anti* steps. Imino protons for all guanines were determined through ^{1}H-^{13}C HMBC spectra, correlating H8 and H1 via $^{13}C5$ (Figure S10). Finally, homopolarity of all G-tetrads is confirmed by looking at characteristic patterns of GH8-imino and imino-imino NOE contacts (Figure S8C and D). Thus, the direction of Hoogsteen hydrogen bonds within tetrads points along G1-G6-G12-G16, G2-G7-G13-G17, and G22-G8-G14-G18.

The four intervening sequences of the quadruplex form two 3-nt propeller loops, one 1-nt propeller loop and one 3-nt lateral snapback loop. A21 of the lateral snapback loop is expected to stack onto the outer tetrad as indicated by various NOE contacts between adenine aromatic protons to guanine imino protons. The third base A5 of the first 3-nt propeller loop seems to be located below the 5'-outer tetrad with several NOE contacts between adenosine A5 protons and 5'-tetrad guanines including A5H1'-G6H8, A5H8-G1H1, A5H2-G1H1, and A5H2-G6H1. In contrast, 3'-residue A11 of the second 3-nt propeller loop seems to only make contacts with residues of the propeller loop itself (Figure S8) except for a single weak crosspeak observed between A11 H2 and G13 H8 located in the central tetrad (not shown). It should be noted, however, that the first propeller loop only bridges two tetrad layers.

Sugar conformations were determined through an analysis of H1'-H2' and H1'-H2" DQF-COSY crosspeaks following a stereospecific assignment of H2'/H2" sugar protons through NOESY experiments at short mixing times (Figure S11). All residues except for G8, G9, A21, and G22 were assigned a *south* conformation. A21 was assigned a *north* conformation due to its strong H1'-H2" DQF-COSY crosspeak. Whereas G8 and T9 sugar puckers could not be evaluated due to their isochronous H2'/H2" resonances, puckering of the G22 sugar remained ambiguous.

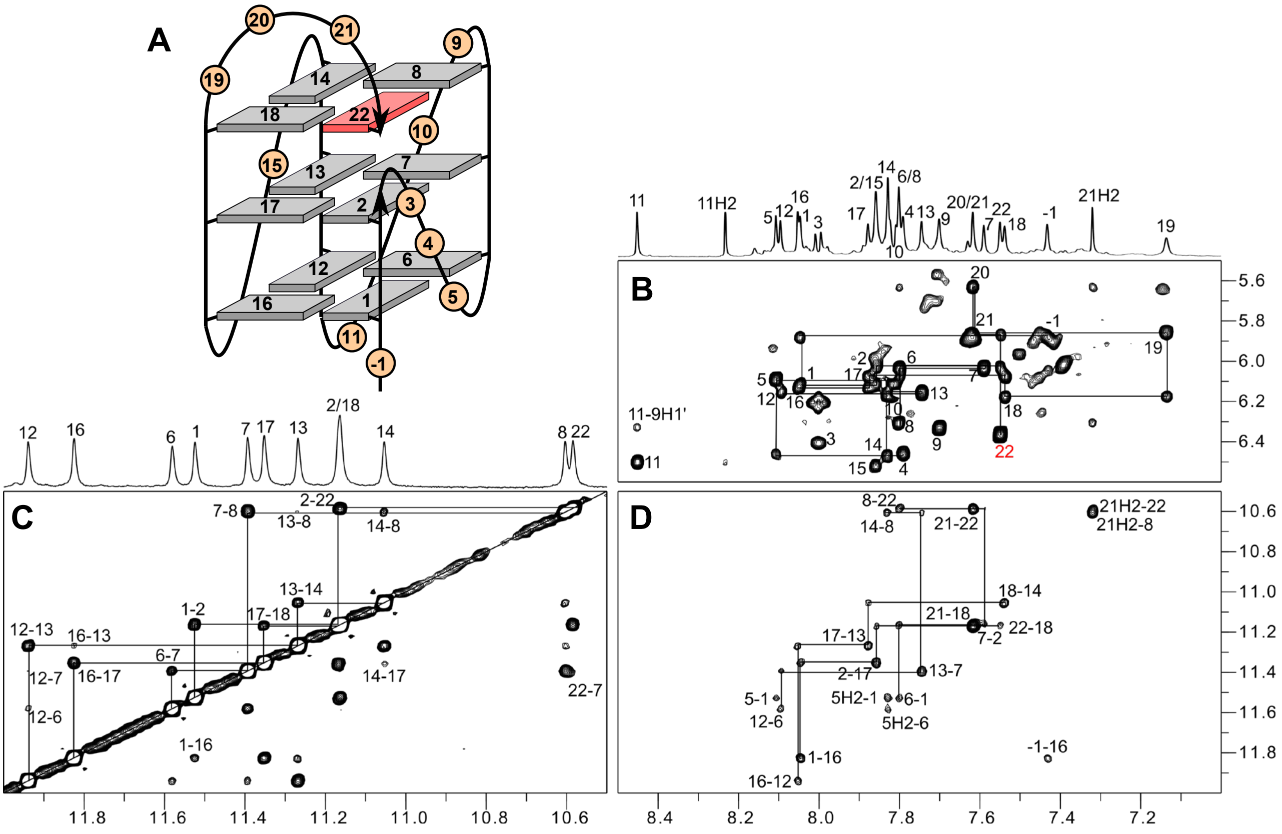

Figure S8. Topology and 2D NOESY spectral regions of $^{5'T}Q$ in 10 mM K^+ buffer, pH 7.0 (30 °C, mixing time 300 ms). (A) Schematic representation with numbered residues of a parallel G-quadruplex with snapback loop adopted by $^{5'T}Q$; *anti*- and *syn*-guanosines of the G-core are colored grey and red, respectively. (B) H6/H8(ω_2)-H1'(ω_1) 2D NOE spectral region tracing continuous intra-nucleotide and sequential connectivities; intra-nucleotide crosspeak of *syn*-G22 is labelled in red. (C) H1(ω_2)-H1(ω_1) crosspeaks with sequential contacts traced along the G tracts. (D) H8/H2(ω_2)-H1(ω_1) NOE contacts with typical intra-tetrad GH8-GH1 connectivities.

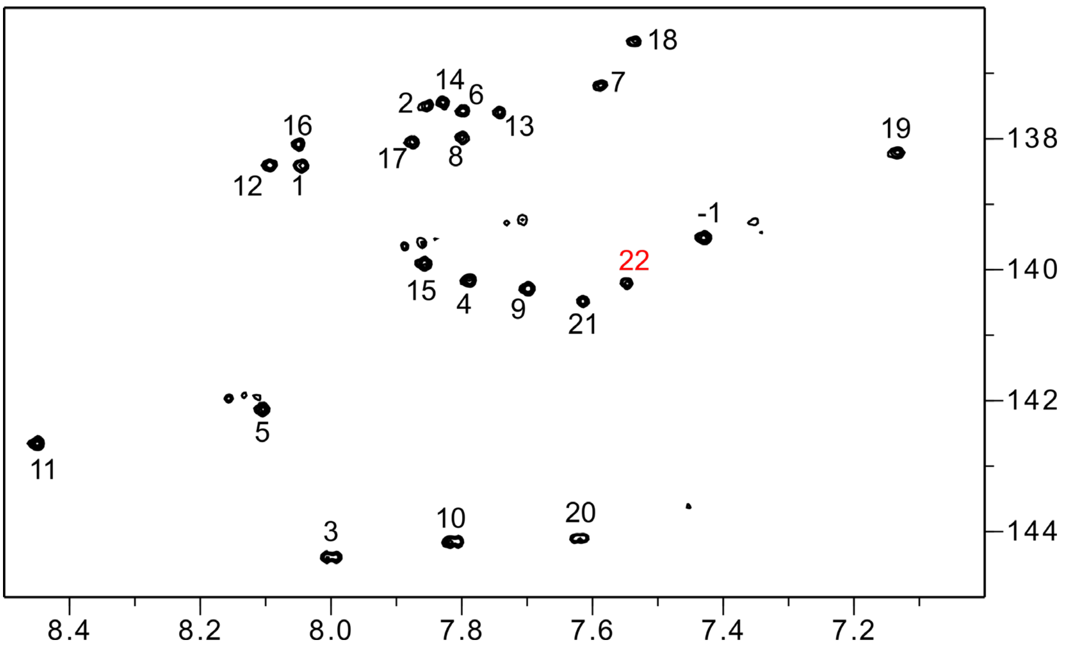

Figure S9. ^{1}H-^{13}C HSQC spectrum of *$^{5'T}Q$* acquired at 30 °C in 10 mM K^{+} buffer, pH 7.0, showing H8/H6–C8/C6 correlations. Crosspeak of *syn*-G22 is labelled in red.

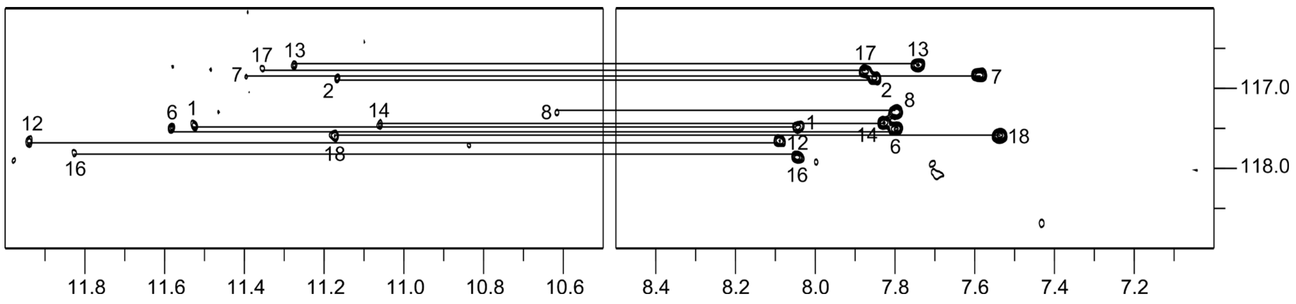

Figure S10. ^{1}H-^{13}C HMBC spectrum of *$^{5'T}Q$* at 30 °C in 10 mM K^{+} buffer, pH 7.0, showing through-bond correlations of guanine H1(ω_2) and H8(ω_2) protons via long-range couplings to $^{13}C5$ (ω_1) at natural abundance.

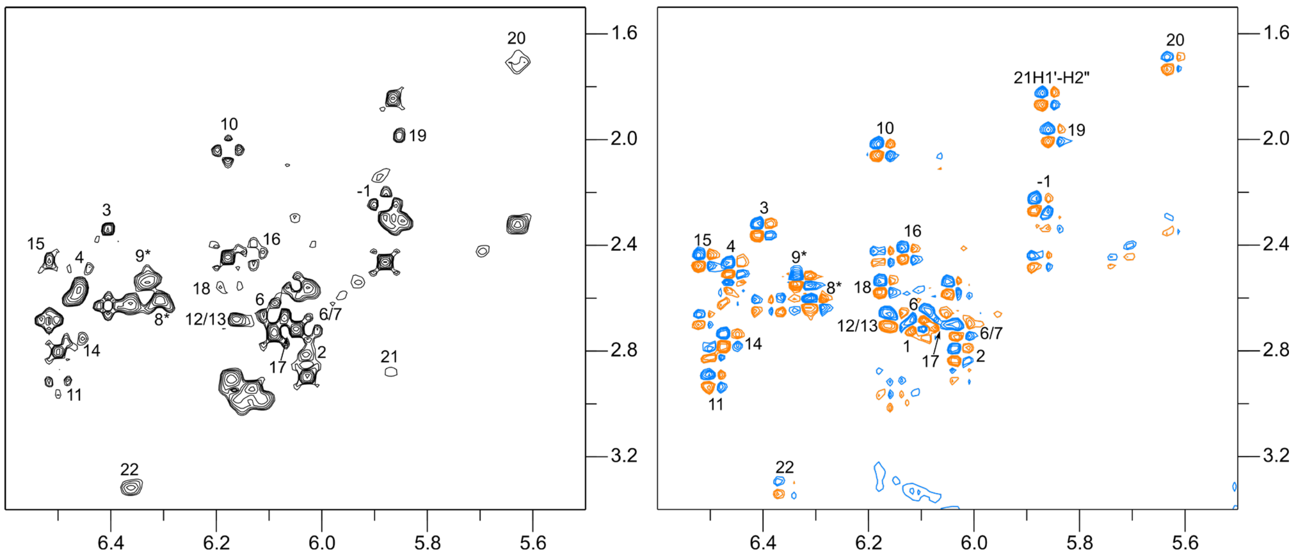

Figure S11. Sugar pucker analysis of *$^{5'T}$Q*. (Left) Stereospecific assignments of H2'/H2" with H1'(ω_2)-H2'/H2"(ω_1) NOESY spectral region at short mixing time (80 ms); crosspeak intensities allow discrimination between H2' and H2". (Right) DQF-COSY spectral region showing H1'(ω_2)-H2'/H2"(ω_1) crosspeaks; *north*- and *south*-type sugar puckers are associated with different scalar couplings and thus different crosspeak patterns of in-phase and anti-phase components.

Table S2. ^{1}H and ^{13}C chemical shifts δ of $^{5'T}$*Q*.[a]

δ (ppm)	H8/H6	H1/H3	H1'	H2'/H2"	H3'	H5/H2/Me	C8/C6	C2
T-1	7.43	n.d.	5.88	2.25/2.47	4.74	1.43	139.52	-
G1	8.05	11.53	6.12	2.70/2.98	5.01	-	138.41	-
G2	7.86	11.16	6.03	2.82/2.57	5.09	-	137.50	-
C3	8.00	-	6.40	2.34/2.63	4.78	6.20	144.40	-
T4	7.79	n.d.	6.46	2.49/2.57	4.95	2.03	140.16	-
A5	8.11	-	6.09	2.66/2.74	4.96	7.83	142.15	154.76
G6	7.80	11.58	6.03	2.72/2.90	5.00	-	137.57	-
G7	7.59	11.40	6.03	2.72/2.56	4.88	-	137.17	-
G8	7.80	10.61	6.31	2.62/2.62	4.99	-	137.98	-
T9	7.70	n.d.	6.33	2.54/2.54	4.91	2.00	140.29	-
C10	7.82	-	6.18	2.05/2.45	4.81	6.11	144.15	-
A11	8.45	-	6.50	2.91/2.81	5.10	8.23	142.66	155.41
G12	8.09	11.94	6.16	2.68/2.99	5.00	-	138.40	-
G13	7.74	11.27	6.17	2.68/2.95	5.02	-	137.60	-
G14	7.83	11.06	6.47	2.76/2.60	5.14	-	137.44	-
T15	7.86	n.d.	6.52	2.46/2.68	5.12	1.98	139.92	-
G16	8.05	11.83	6.13	2.43/2.94	5.15	-	138.08	-
G17	7.88	11.36	6.07	2.71/2.68	5.08	-	138.05	-
G18	7.54	11.17	6.17	2.56/2.91	4.95	-	136.50	-
T19	7.13	n.d.	5.85	1.99/2.32	4.69	1.61	138.21	-
C20	7.62	-	5.63	1.71/2.32	4.68	5.88	144.11	-
A21	7.62	-	5.87	2.89/1.85	4.51	7.32	140.48	152.81
G22	7.55	10.59	6.36	3.32/2.63	4.84	-	140.21	-

[a]At 30 ºC in 10 mM potassium phosphate buffer, pH 7.0.

Table S3. NMR restraints and structural statistics of calculated structures.

sequence	*^{6Br}Q*	*$^{5'T}Q$*
NOE distance restraints		
intra-residual	86	92
inter-residual	122	141
exchangeable	41	53
repulsion	6	0
other restraints:		
hydrogen bonds	48	48
dihedral angles	41	43
planarity	3	3
structural statistics:		
pairwise heavy atom RMSD value (Å)		
all residues	2.0 ± 0.6	2.5 ± 0.6
G-tetrad core	0.7 ± 0.1	0.9 ± 0.2
NOE violations:		
maximum violation (Å)	0.16	0.17
mean NOE violation (Å)	0.002 ± 0.001	0.002 ± 0.001
deviations from idealized geometry:		
bond lengths (Å)	0.01 ± 0.0001	0.01 ± 0.0001
bond angles (degree)	2.3 ± 0.03	2.2 ± 0.04

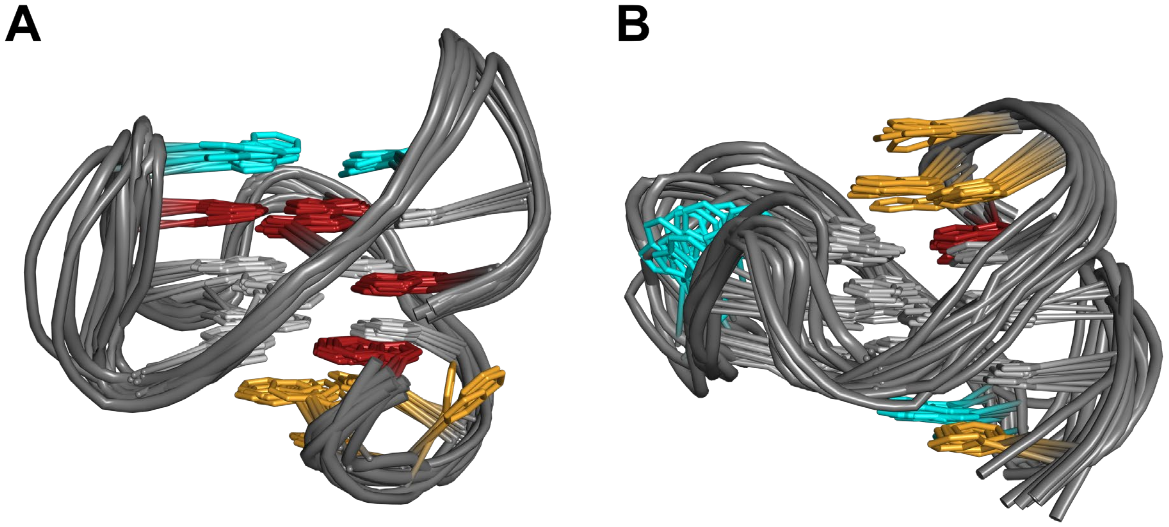

Figure S12. Superposition of ten lowest-energy structures for (A) *^{6Br}Q* and (B) *$^{5'T}$Q*; loop residues C3, T4, T9, C10, and T15 are omitted for clarity; *anti*- and *syn*-guanosines are colored in grey and red, respectively; A5 and A11 are colored in cyan and T(-1) in *$^{5'T}$Q* as well as snapback lateral loop residues at the bottom (A) and top (B) are colored in orange.

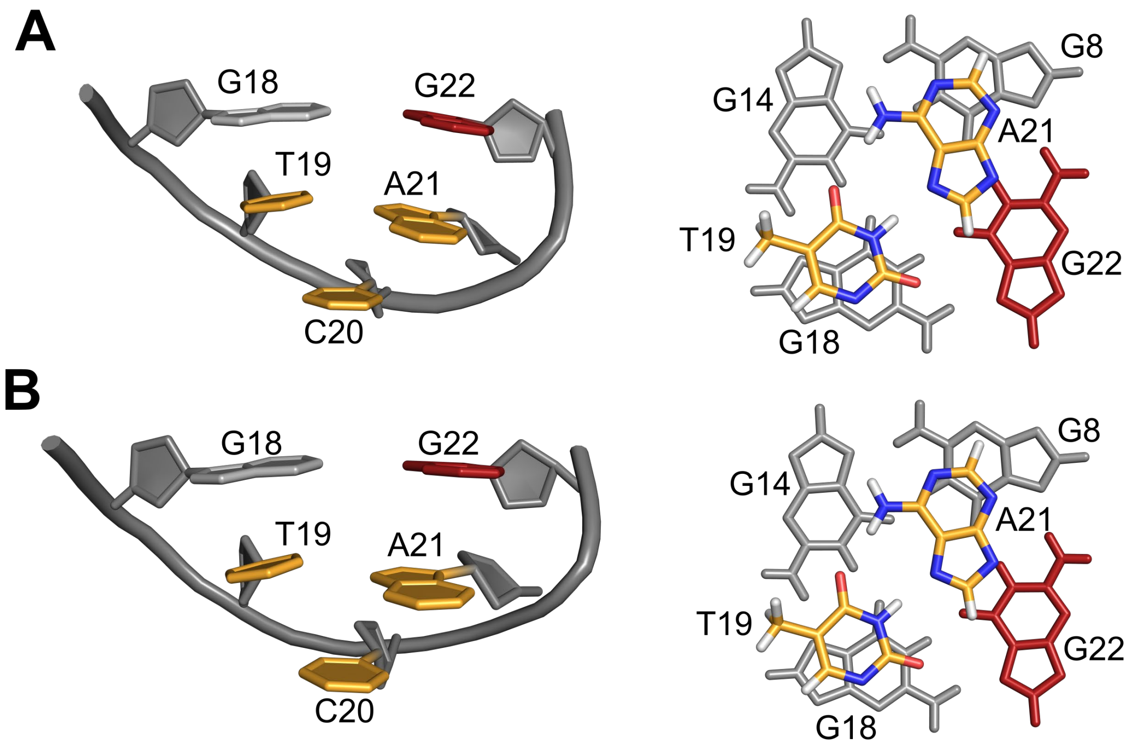

Figure S13. TCA lateral snapback loop of (A) *^{6Br}Q* and (B) *$^{5'T}$Q* in side view (left) and top view (right) showing stacking of the Hoogsteen T19·A21 base pair onto the outer G-tetrad. C20 is additionally stacked onto the base pair (not shown in top view).

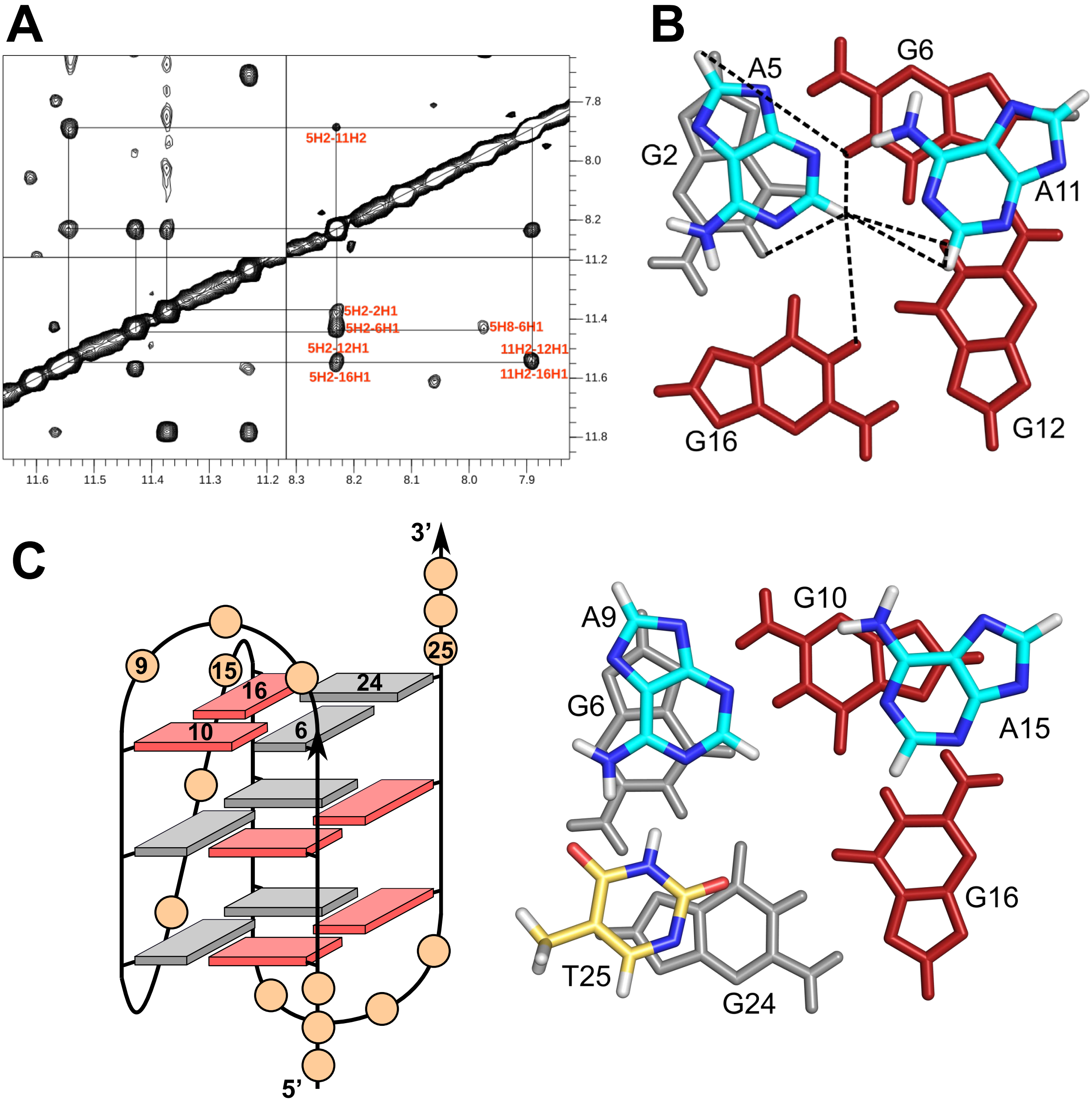

Figure S14. (A) 2D NOESY spectral regions of *6BrQ*, highlighting some key NOE crosspeaks that define the position of A5 in the first lateral loop and of A11 in the second propeller loop above the outer tetrad. (B) Top view of the *6BrQ* outer tetrad with capping loop residues; A5 stacks over G2 and forms a putative hydrogen bond with the A11 amino proton; experimentally observed NOE contacts are indicated by dotted lines. (C) Schematic representation of a +(lpl) G-quadruplex formed by a derivative of a 27-nt human telomeric sequence in Na^+ solution (right); its solution structure shows a corresponding positioning of A9 in the first lateral loop and A15 in the second propeller loop, both capping the upper tetrad (left); also shown is T25 of the 3'-flanking sequence forming an additional Watson-Crick base pair with A9 (PDB 2MBJ).

NMR spectral analysis for *Q-5T* and *Q-11T*

Assignments for *Q-5T* revealed its folding into a major parallel species with a snapback loop as evident by eleven *anti*-Gs and one 3'-terminal *syn*-G filling the vacant position of the G-tetrad (Figures S15 and S16). Interestingly, assignments for *Q-11T* clearly demonstrated its alternate folding into a major +(lpp) hybrid fold with crosspeak patterns very similar to ^{6Br}Q and *Qref* (Figures S17 and S18). These results indicate the critical role of A5 but not of A11 as contributor for a major +(lpp) fold.

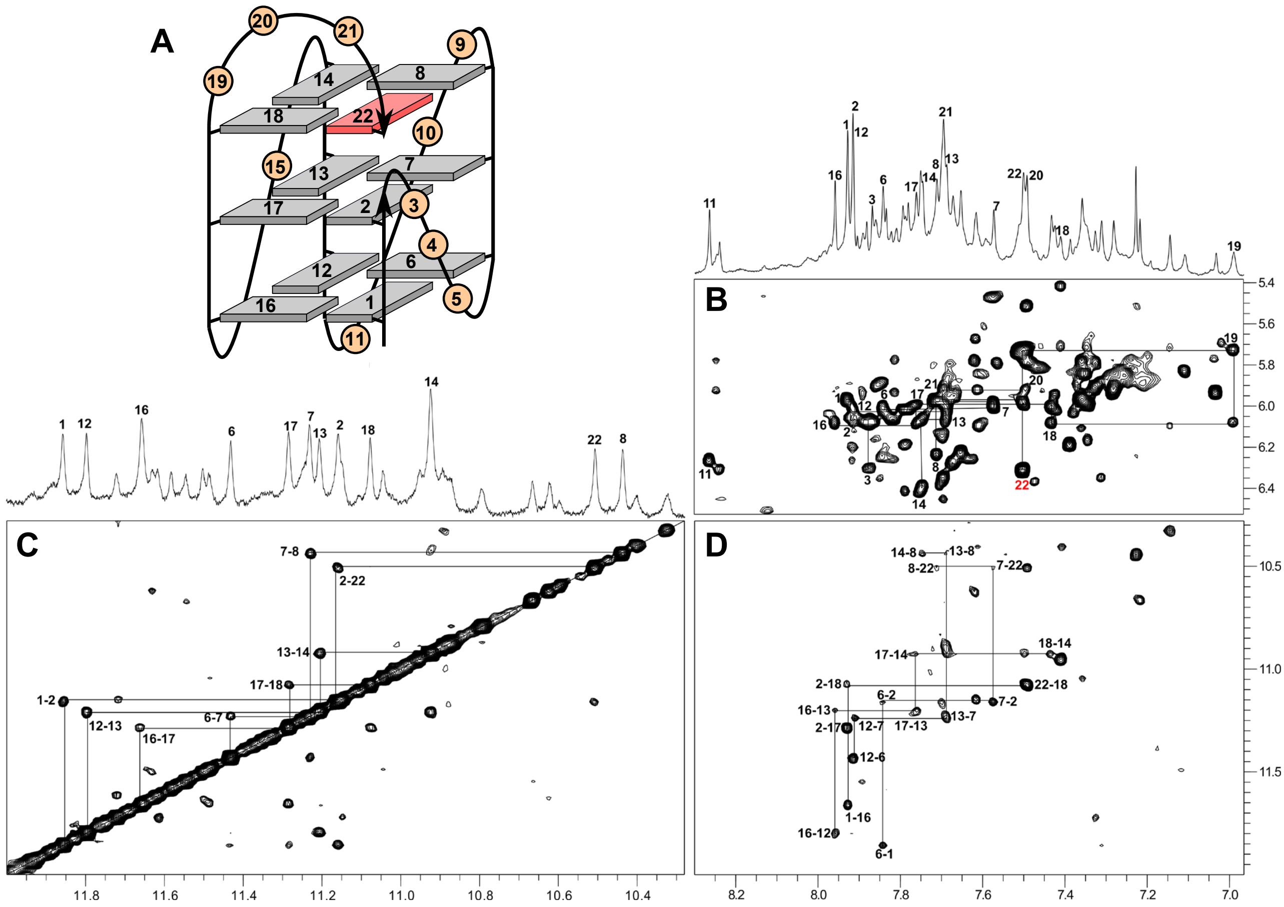

Figure S15. Topology and 2D NOESY spectral regions of *Q-5T* in 10 mM K$^+$ buffer, pH 7.0 (20 °C, mixing time 300 ms). (A) Schematic representation with numbered residues of a parallel G-quadruplex with snapback loop adopted by *Q-5T*; *anti*- and *syn*-guanosines of the G-core are colored grey and red, respectively. (B) H6/H8(ω_2)-H1'(ω_1) 2D NOE spectral region tracing continuous intra-nucleotide and sequential connectivities; intra-nucleotide crosspeak of *syn*-G22 is labelled in red. (C) H1(ω_2)-H1(ω_1) crosspeaks with sequential contacts traced along the G tracts. (D) H8(ω_2)-H1(ω_1) NOE contacts with typical intra-tetrad GH8-GH1 connectivities.

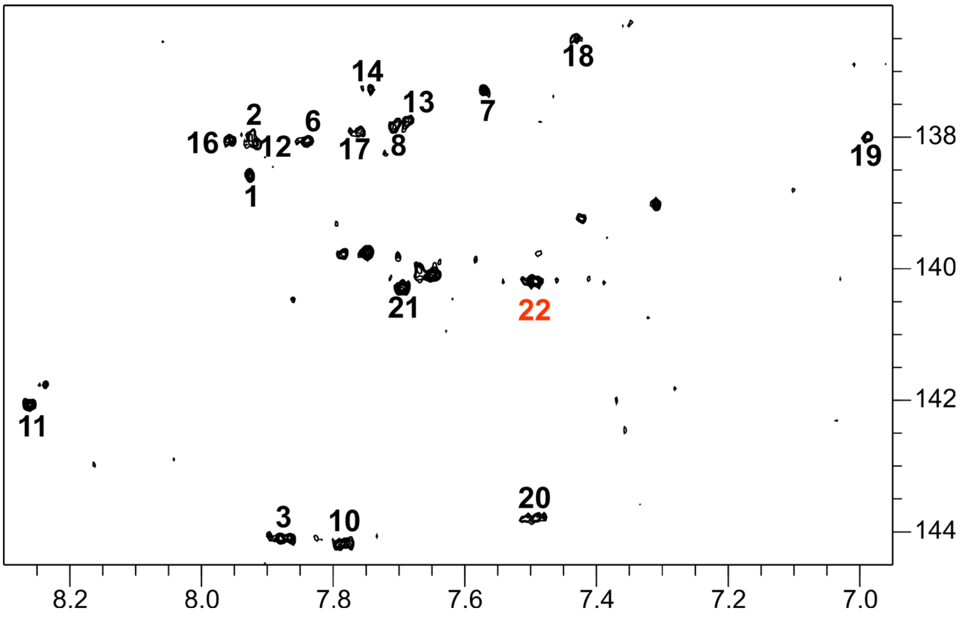

Figure S16. ^{1}H-^{13}C HSQC spectrum of *Q-5T* acquired at 20 °C in 10 mM K^{+} buffer, pH 7.0, showing H8/H6–C8/C6 correlations. Crosspeak of *syn*-G22 is labelled in red.

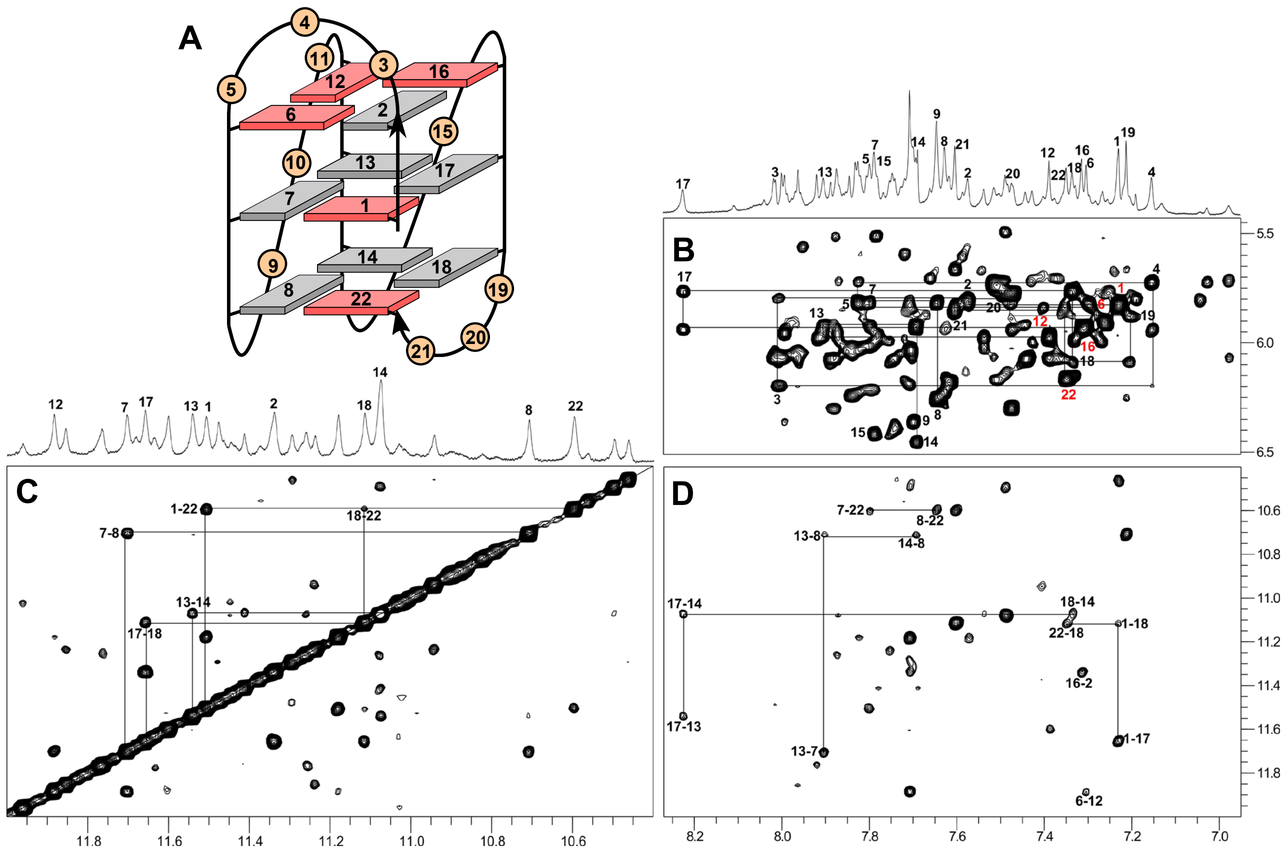

Figure S17. Topology and 2D NOESY spectral regions of *Q-11T* in 10 mM K^+ buffer, pH 7.0 (20 °C, mixing time 300 ms). (A) Schematic representation with numbered residues of a (3+1) hybrid-type G-quadruplex with a (+lpp) topology and snapback loop adopted by *Q-11T*; *anti*- and *syn*-guanosines of the G-core are colored grey and red, respectively. (B) H6/H8(ω_2)-H1'(ω_1) 2D NOE spectral region tracing continuous intra-nucleotide and sequential connectivities; intra-nucleotide crosspeaks of *syn*-guanosines are labelled in red. (C) H1(ω_2)-H1(ω_1) crosspeaks with sequential contacts traced along the G tracts. (D) H8(ω_2)-H1(ω_1) NOE contacts with typical intra-tetrad GH8-GH1 connectivities.

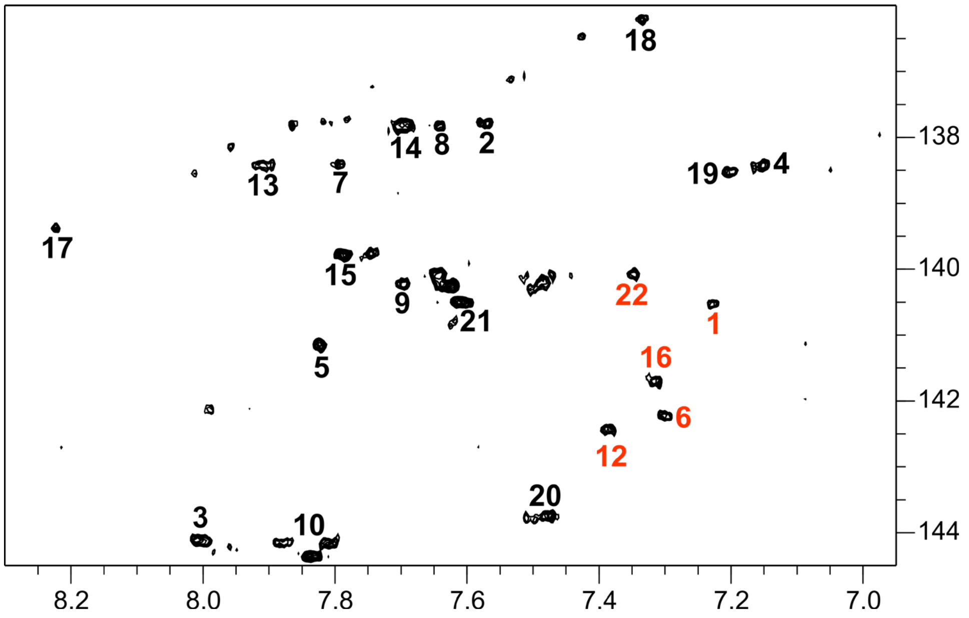

Figure S18. ^{1}H-^{13}C HSQC spectrum of *Q-11T* acquired at 20 °C in 10 mM K^{+} buffer, pH 7.0, showing H8/H6–C8/C6 correlations. Crosspeaks of *syn*-guanosines are labelled in red.

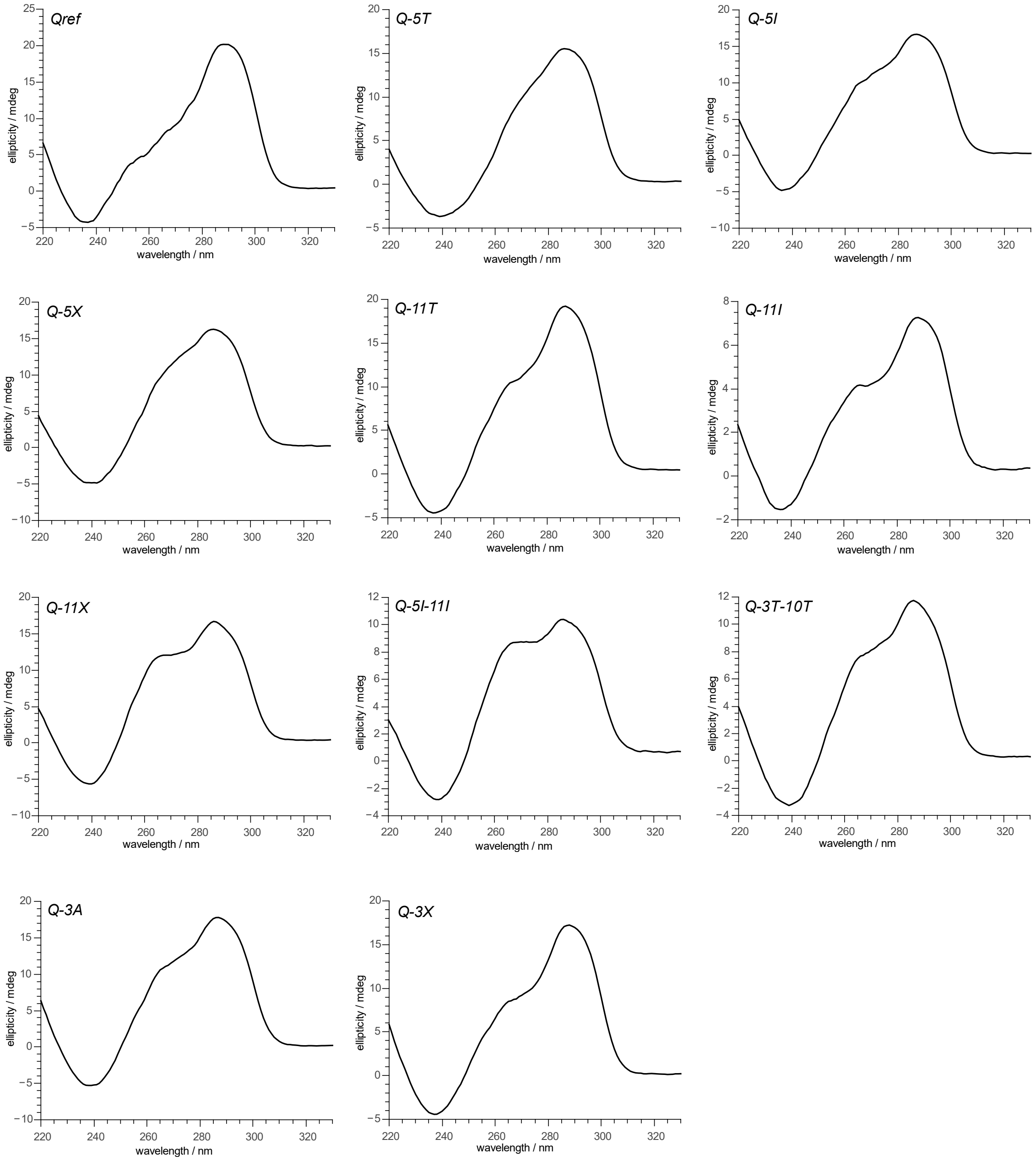

Figure S19. CD spectra of *Qref*-derived sequences with single and double mutations in 10 mM potassium phosphate buffer, pH 7, at 20 °C.

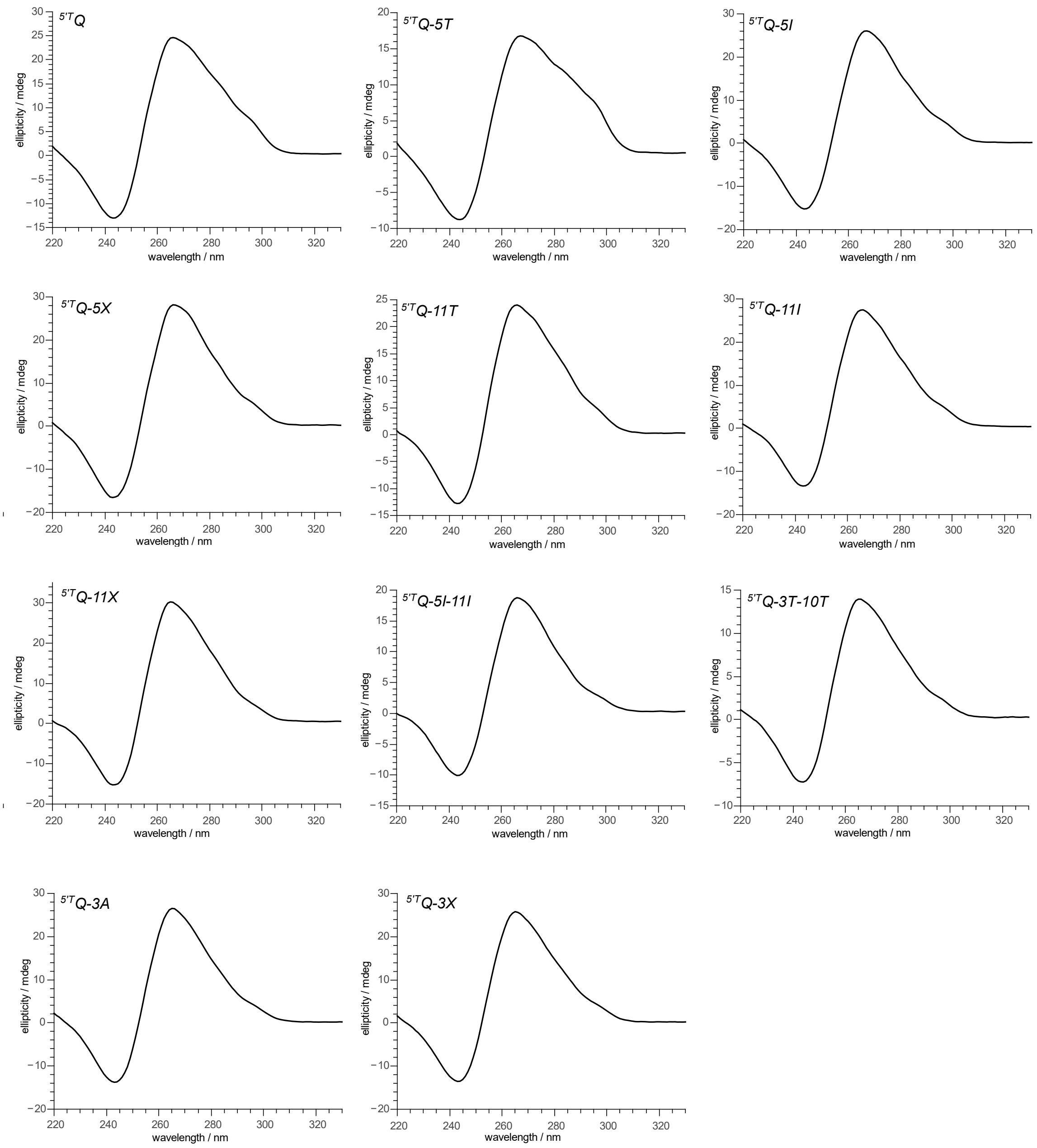

Figure S20. CD spectra of *$^{5'T}Q$*-modified *Qref*-derived sequences in 10 mM potassium phosphate buffer, pH 7, at 20 °C.

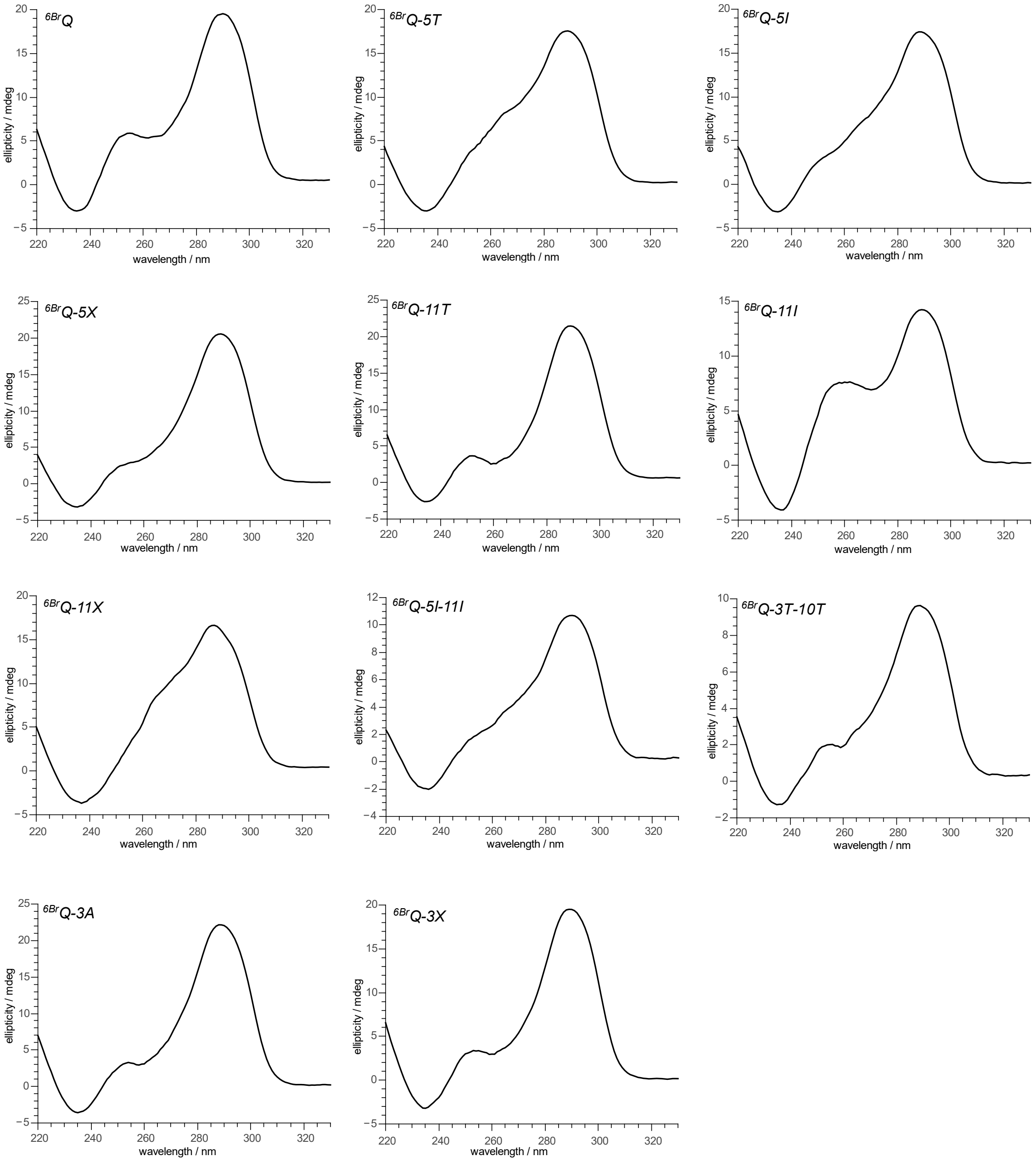

Figure S21. CD spectra of ^{6Br}Q-modified *Qref*-derived sequences in 10 mM potassium phosphate buffer, pH 7, at 20 °C.

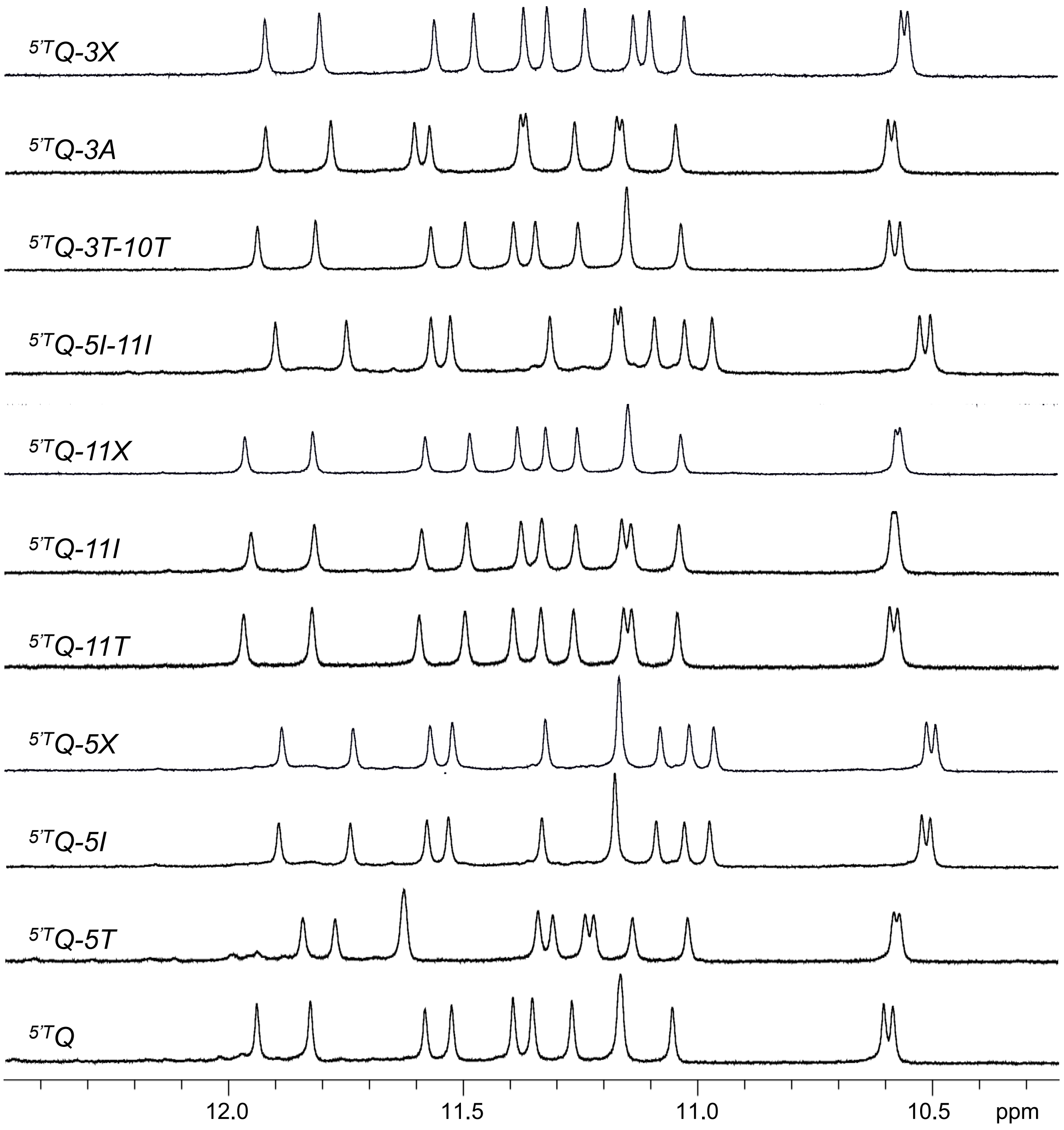

Figure S22. Imino proton spectral region of $^{5'T}$*Q*-modified mutants. NMR spectra were acquired in 10 mM potassium phosphate buffer, pH 7.0, at 30 °C.

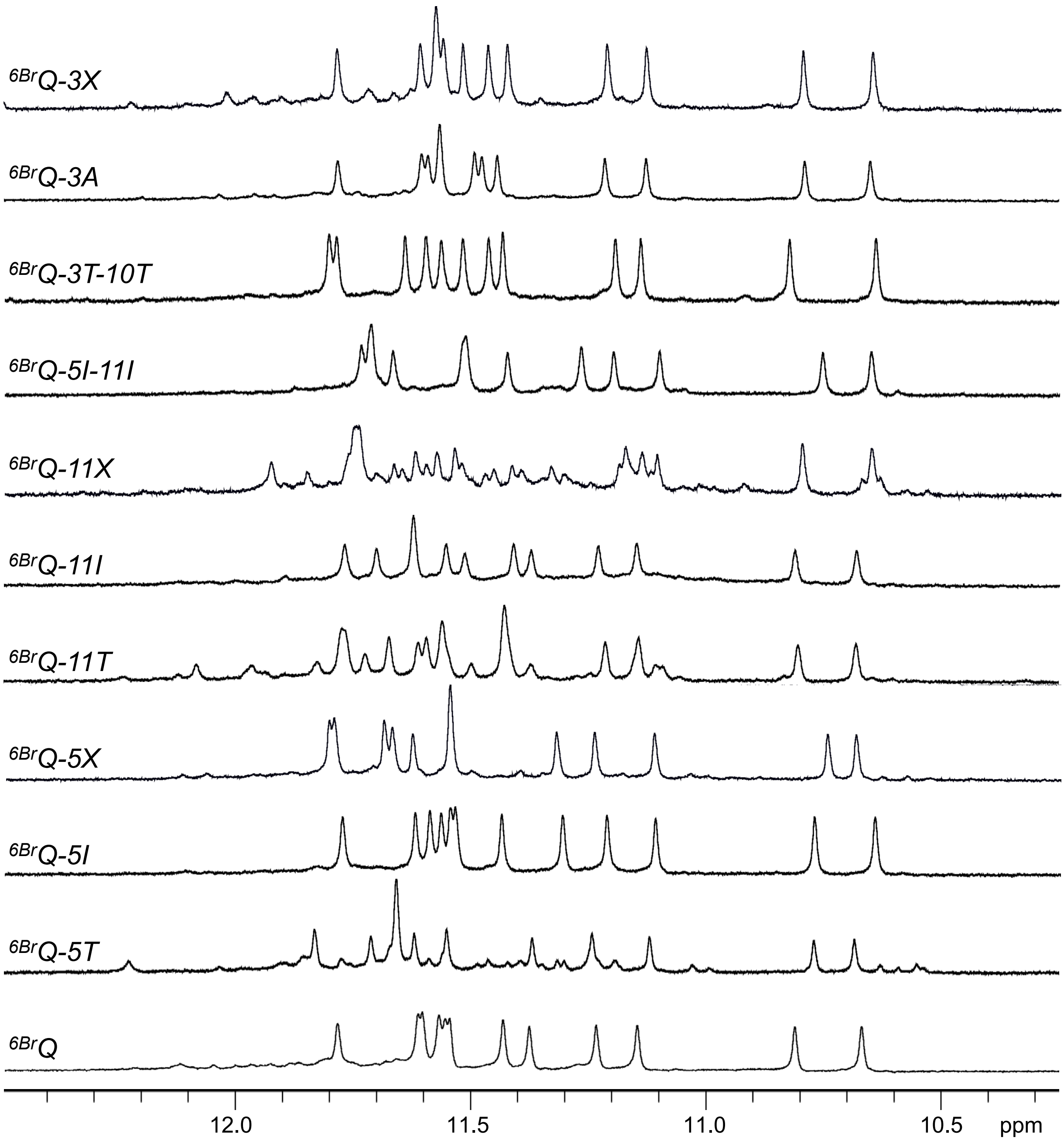

Figure S23. Imino proton spectral region of ^{6Br}Q-modified mutants. NMR spectra were acquired in 10 mM potassium phosphate buffer, pH 7.0, at 30 °C.

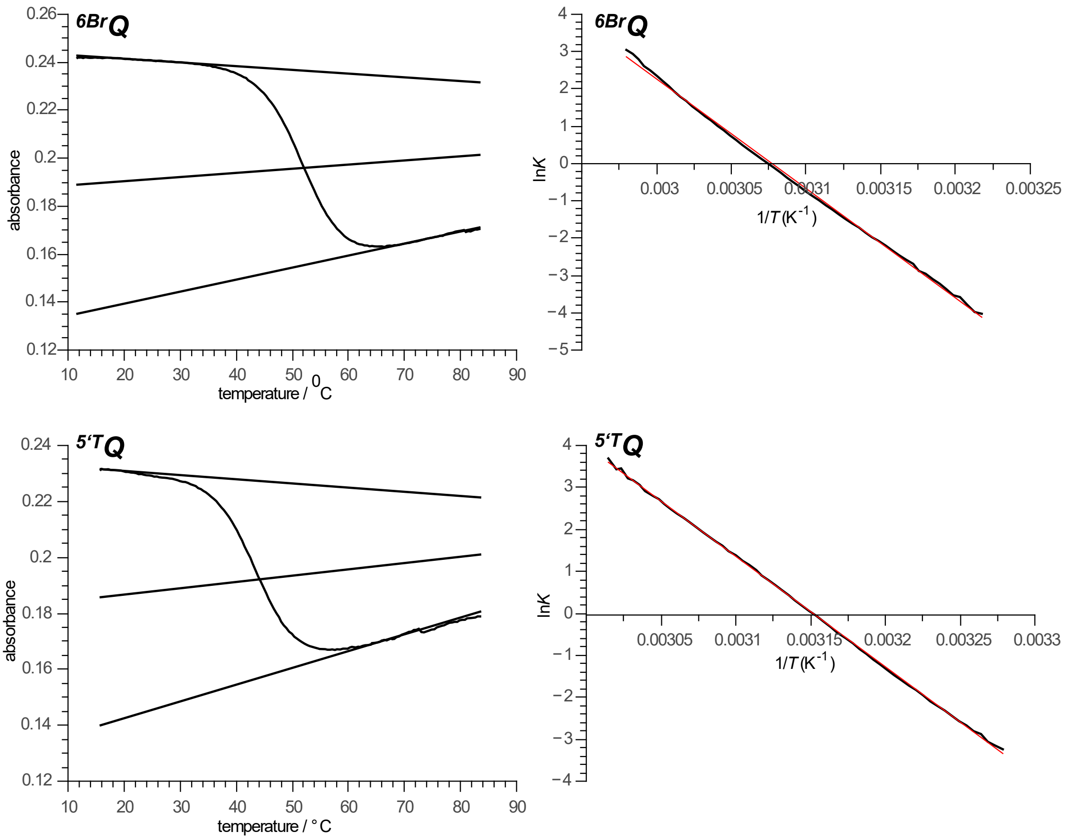

Figure S24. Exemplary van't Hoff analysis of quadruplex melting for the ^{6Br}Q (top) and $^{5'T}Q$ sequence (bottom). (Left) Melting curves with fits of upper and lower baseline for the determination of temperature dependent populations. (Right) Corresponding van't Hoff plot with a superimposed linear fit curve (red).

Table S4. Thermodynamic parameters for quadruplex formation with +(lpp) topology at 30 °C.[a]

oligonucleotide	T_m (°C)	$\Delta H°$ (kcal/mol)[b]	$-T\Delta S°$ (kcal/mol)[c]	$\Delta G°_{30}$ (kcal/mol)[d]
^{6Br}Q	51.5 ± 1.2	-56.1 ± 0.9	52.4 ± 0.9	-3.7 ± 0.2
$^{6Br}Q\text{-}3A$	46.0 ± 0.9	-52.8 ± 0.9	50.1 ± 1.0	-2.7 ± 0.1
$^{6Br}Q\text{-}3X$[e]	54.2 ± 0.6	-64.7 ± 0.6	59.9 ± 0.7	-4.8 ± 0.1
$^{6Br}Q\text{-}5T$	49.5 ± 0.5	-56.0 ± 2.4	52.6 ± 2.3	-3.4 ± 0.2
$^{6Br}Q\text{-}5I$	51.5 ± 0.3	-58.9 ± 1.2	55.0 ± 1.1	-3.9 ± 0.1
$^{6Br}Q\text{-}5X$[e]	45.2 ± 0.2	-55.2 ± 1.0	52.6 ± 1.0	-2.7 ± 0.1
$^{6Br}Q\text{-}11T$	51.7 ± 0.7	-58.1 ± 1.9	54.2 ± 1.8	-3.9 ± 0.1
$^{6Br}Q\text{-}11I$	51.9 ± 0.6	-58.8 ± 1.7	54.8 ± 1.5	-4.0 ± 0.2
$^{6Br}Q\text{-}5I\text{-}11I$	50.3 ± 0.2	-59.6 ± 2.0	55.9 ± 1.8	-3.8 ± 0.2
$^{6Br}Q\text{-}3T\text{-}10T$[e]	50.8 ± 0.3	-58.1 ± 0.3	54.3 ± 0.2	-3.8 ± 0.1

[a]Average values with standard deviations derived from the analysis of three independent UV melting experiments. [b]Determined from a van't Hoff plot. [c]$\Delta S° = \Delta H°/T_m$. [d]$\Delta G° = \Delta H° - T\Delta S°$. [e]X = abasic 1',2'-dideoxyribose residue.

Table S5. Thermodynamic parameters for quadruplex formation with -(ppp) topology at 30 °C.[a]

oligonucleotide	T_m (°C)	$\Delta H°$ (kcal/mol)[b]	$-T\Delta S°$ (kcal/mol)[c]	$\Delta G°_{30}$ (kcal/mol)[d]
^{5T}Q	44.1 ± 0.3	-53.7 ± 1.9	51.3 ± 1.7	-2.4 ± 0.1
$^{5T}Q\text{-}3A$	39.4 ± 0.7	-52.8 ± 1.0	51.2 ± 0.8	-1.6 ± 0.1
$^{5T}Q\text{-}3X$[e]	46.6 ± 0.5	-54.7 ± 0.7	51.8 ± 0.6	-2.9 ± 0.1
$^{5T}Q\text{-}5T$	44.0 ± 0.4	-51.1 ± 1.7	48.8 ± 1.7	-2.3 ± 0.1
$^{5T}Q\text{-}5I$	46.4 ± 0.8	-61.7 ± 2.2	58.5 ± 2.1	-3.2 ± 0.2
$^{5T}Q\text{-}5X$[e]	47.4 ± 0.5	-59.9 ± 1.9	56.7 ± 1.8	-3.3 ± 0.1
$^{5T}Q\text{-}11T$	46.0 ± 0.3	-57.0 ± 1.1	54.1 ± 1.0	-2.9 ± 0.1
$^{5T}Q\text{-}11I$	44.3 ± 0.5	-55.8 ± 1.7	53.3 ± 1.7	-2.5 ± 0.1
$^{5T}Q\text{-}5I\text{-}11I$	47.9 ± 0.5	-61.5 ± 0.2	58.0 ± 0.3	-3.5 ± 0.1
$^{5T}Q\text{-}3T\text{-}10T$	42.9 ± 0.3	-56.9 ± 0.8	54.5 ± 0.8	-2.4 ± 0.1

[a]Average values with standard deviations derived from the analysis of three independent UV melting experiments. [b]Determined from a van't Hoff plot. [c]$\Delta S° = \Delta H°/T_m$. [d]$\Delta G° = \Delta H° - T\Delta S°$. [e]X = abasic 1',2'-dideoxyribose residue.

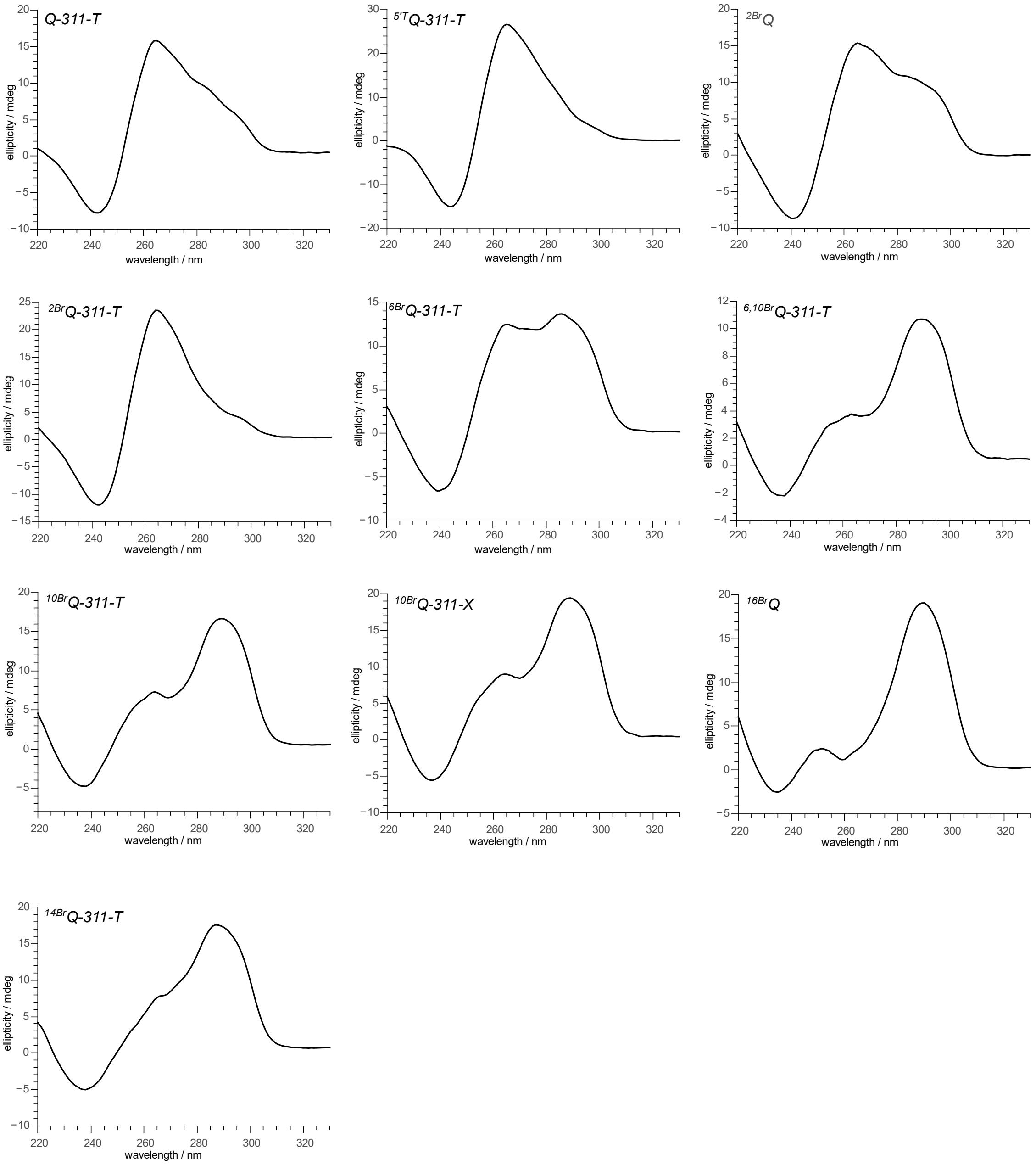

Figure S25. CD spectra of ^{2Br}Q, ^{16Br}Q, and sequences with a 311 loop length arrangement in 10 mM potassium phosphate buffer, pH 7, at 20 °C.

Gel electrophoresis. Gel electrophoresis was performed on a 15% polyacrylamide gel (acrylamide:bisacrylamide 19:1). DNA (25 µM dissolved in 10 mM potassium phosphate buffer, pH 7.0) was loaded and separation was performed at 4 °C in 1x TBE buffer supplemented with 10 mM KCl and a voltage of 200 V. Gels were visualized by staining with a 5 µM thiazole orange solution.

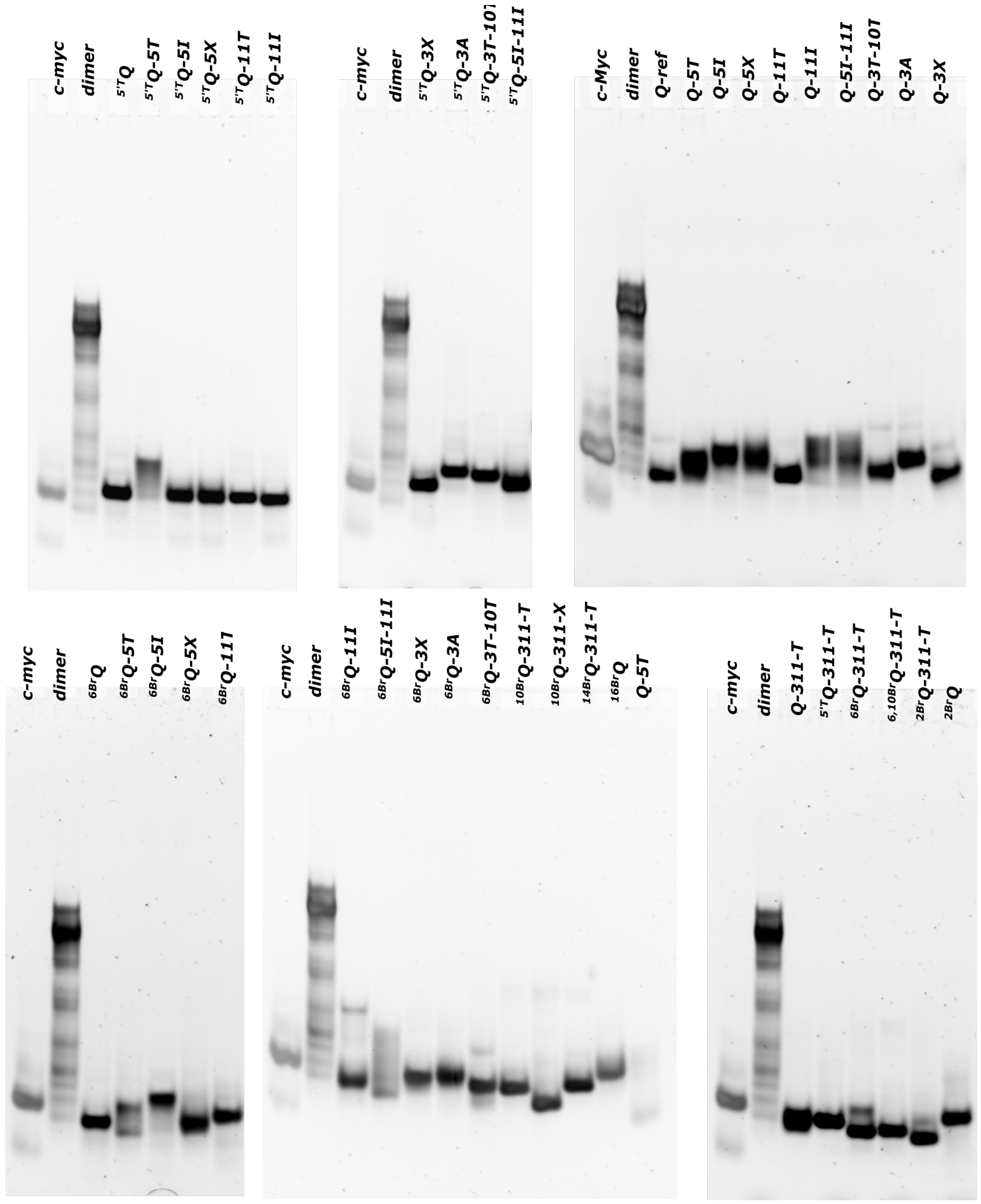

Figure S26. Non-denaturing polyacrylamide gel electrophoresis of quadruplex-forming sequences; a parallel *c-myc* quadruplex and a telomeric dimer were used as a reference.

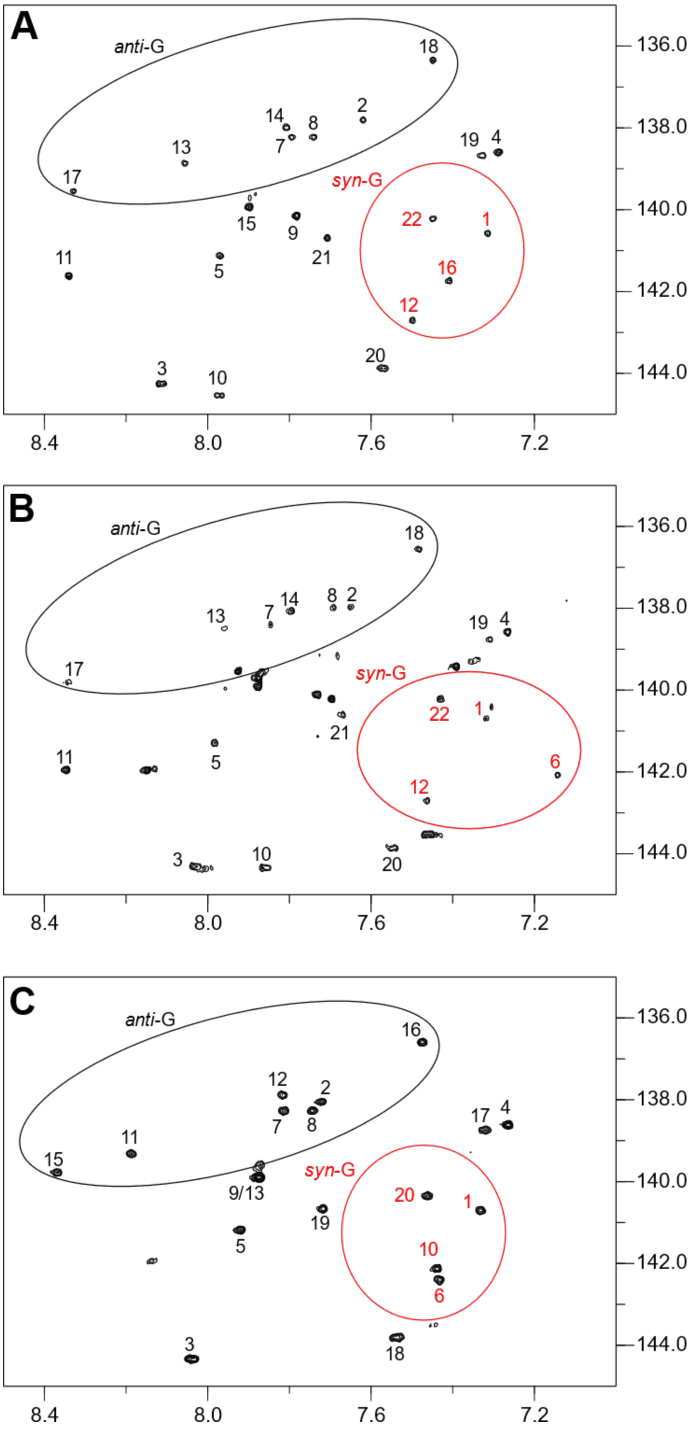

Figure S27. Comparison of ^{1}H-^{13}C HSQC spectra of (A) *^{6Br}Q*, (B) *^{16Br}Q*, and (C) *^{14Br}Q-311-T* acquired at 30 °C in 10 mM K^{+} buffer, pH 7.0, showing H8/H6–C8/C6 correlations. *Anti*-G and *syn*-G residues of the G-core are circled and labelled in black and red color, respectively. The highly similar pattern of C-H correlations with *^{6Br}Q* comprising four *syn*-G and 7 *anti*-G residues for the G-core demonstrate a hybrid-type fold with one broken *syn-syn-anti* and three *syn-anti-anti* G-columns for both *^{16Br}Q* and *^{14Br}Q-311-T*. Note the absence of an observable fifth *syn*-G residue at the ^{Br}G incorporation site.

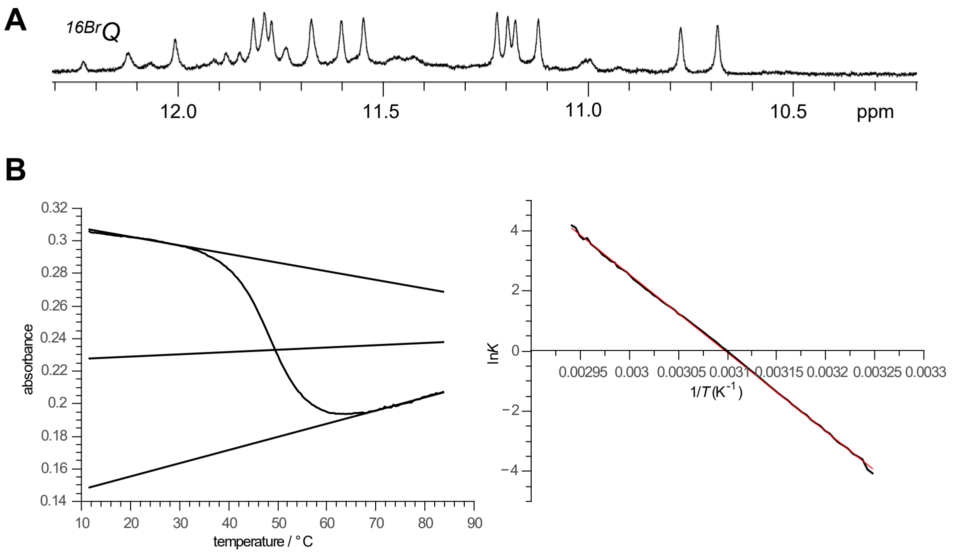

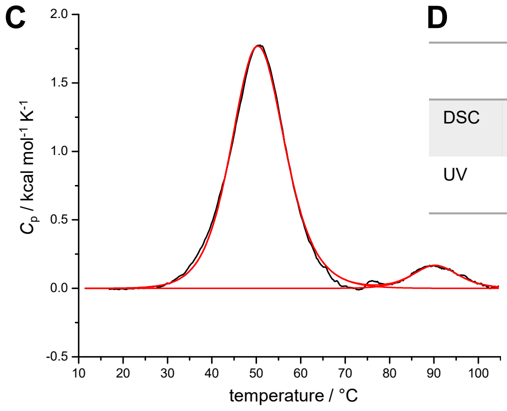

D

	T_m (°C)	$\Delta H°$ (kcal/mol)	$-T\Delta S°$ kcal/mol)	$\Delta G°$ (kcal/mol)
DSC	50.5 ± 0.5	-52.8 ± 1.2	49.4 ± 1.2	-3.4 ± 0.1
UV	49.3 ± 0.2	-53,2 ± 1.3	50.0 ± 1.2	-3.2 ± 0.1

Figure S28. (A) Imino proton NMR spectral region of the *16BrQ* quadruplex acquired in 10 mM potassium phosphate buffer, pH 7.0, at 30 °C. (B) UV melting curve with fits of upper and lower baseline (left) and van't Hoff plot with a superimposed linear fit curve in red (right). (C) DSC thermogram with red fit curve comprising a low-and high-melting transition. (D) Comparison of van't Hoff thermodynamic parameters obtained from UV melting and from the low-temperature DSC transition. Being within experimental uncertainties, the same van't Hoff enthalpies extracted from the UV melting and the low-temperature DSC transition suggests that additional resonances in the NMR spectrum result from some high-melting multimeric associates that do not influence the UV melting transition centered at ~49 °C.

Differential Scanning Calorimetry (DSC) measurements. DSC experiments were carried out with a VP-DSC instrument (Malvern Instruments, United Kingdom). The oligonucleotide solution (50 μM) was heated up to 105 °C with a heating rate of 0.5 °C·min^{-1}. Data from a buffer vs. buffer scan were subtracted from the sample data. After cubic baseline correction, data were fitted assuming ΔC_p = 0 kcal·mol^{-1}·K^{-1} to obtain T_m and $\Delta H°_{vH}$. Parameters are averages over three independent experiments.

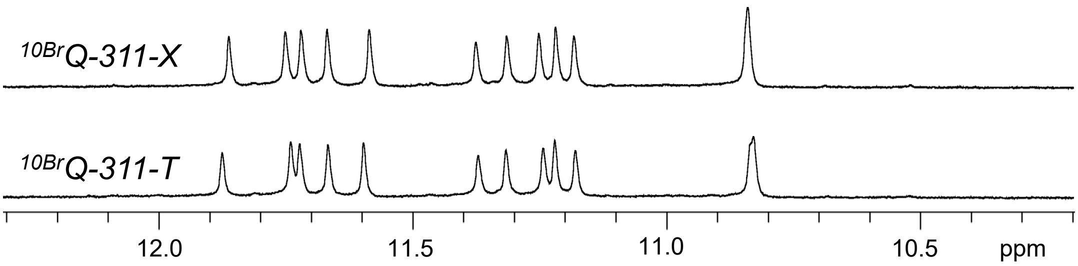

Figure S29. Imino proton NMR spectral region of *10BrQ-311-T* and *10BrQ-311-X* quadruplexes acquired in 10 mM potassium phosphate buffer, pH 7.0, at 30 °C.

Table S6. Thermodynamic parameters for the formation of *10BrQ-311-T* and *10BrQ-311-X* quadruplexes at 30 °C.[a]

oligonucleotide	T_m (°C)	$\Delta H°$ (kcal/mol)[b]	$-T\Delta S°$ (kcal/mol)[c]	$\Delta G°_{30}$ (kcal/mol)[d]
10BrQ-311-T	58.5 ± 0.6	-70.1 ± 1.2	64.0 ± 1.2	-6.1 ± 0.1
10BrQ-311-X	59.0 ± 0.4	-72.8 ± 1.7	66.4 ± 1.6	-6.4 ± 0.1

[a]Average values with standard deviations derived from the analysis of three independent UV melting experiments. [b]Determined from a van't Hoff plot. [c]$\Delta S° = \Delta H°/T_m$. [d]$\Delta G° = \Delta H° - T\Delta S°$.

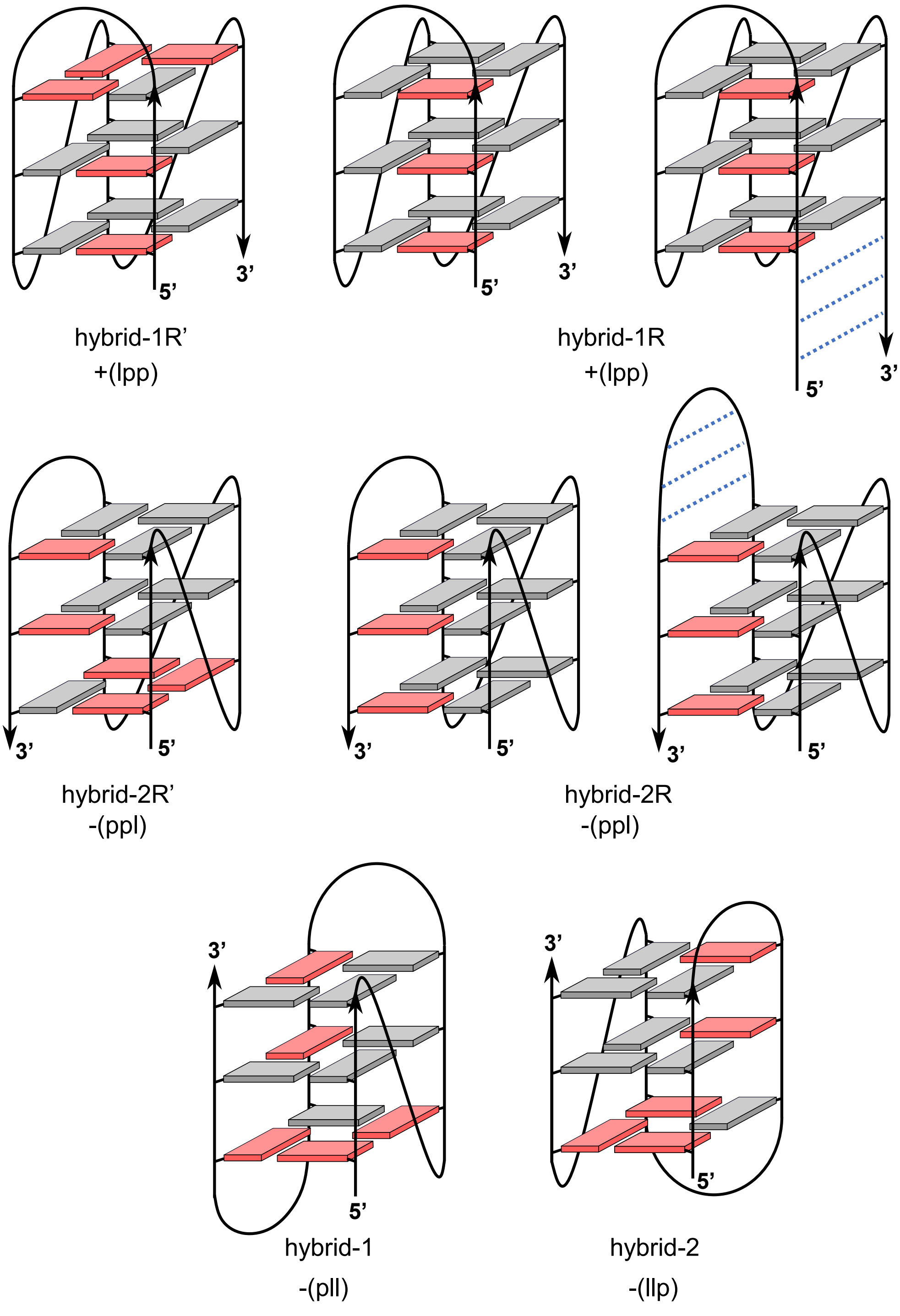

Figure S30. Topologies of (3+1) hybrid quadruplexes with different arrangements of lateral and propeller loops. Due to their all-*syn* antiparallel column, hybrid-1R and hybrid-2R quadruplexes feature only homopolar tetrad stacking as observed in parallel structures; *syn*- and *anti*-G residues are colored red and grey, respectively. For hybrid-1R and hybrid-2R conformations, additional quadruplex-duplex hybrids with Watson-Crick base pairs between flanking sequences or within the lateral loop (dotted blue lines) form a coaxially stacked duplex extension along the quadruplex wide groove, promoting folding into one of the two +(lpp) or –(ppl) topologies (top and middle right).

NMR spectral analysis and resonance assignments for *^{2Br}Q-311-T*

Upon shortening the second intervening sequence to a 1-nt propeller loop, three formed species of *Q-311-T* coexist with the major species adopting a parallel topology like *$^{5'T}$Q* (see Figure 8 of the main manuscript). Minor species can be identified through shifting equilibria by deliberate substitutions with ^{Br}G residues at specific positions. The *^{2Br}Q-311-T* quadruplex with a corresponding modification at the 2-position yields a set of 12 imino proton resonances which can be attributed to the formation of a three-layered G-quadruplex (Figure S30). Two *syn*- and nine *anti*-guanosines can be identified following NOE connectivities and this is confirmed by ^{1}H-^{13}C HSQC spectra (Figure S31). NOE sequential walks along the G-quadruplex allowed to distinguish three columns comprising all-*anti* steps, namely G7-G8-G9, G10-G11-G12, and G14-G15-G16. One column was found to exclusively include *syn-syn* steps, i.e., G20-G1-G2, providing for the missing ^{Br}G2 in a *syn* conformation. Sequential contacts between aromatic and anomeric protons could be traced between G14 to A19 (Figure S30C). Also, uninterrupted sequential contacts link residues from C3 to G9. Additionally, G2 H1' and G2 H3' show weak and strong sequential contacts to C3 H6, respectively. G20 and G1 feature a reverse sequential NOE crosspeak pattern typical for a *syn-syn* step in the H8-H3' spectral region (Figure S30B). G20 can be distinguished from G1 due to a long-range contact to C18. Following assignments in the aromatic-imino and imino-imino spectral region, the corresponding crosspeak pattern confirms a quadruplex topology with exclusive homopolar tetrad stacking (Figure S30D and E). Hoogsteen hydrogen bonds within individual tetrads run along G2-G6-G10-G14, G1-G7-G11-G15, and G20-G8-G12-G16. Taken together, *^{2Br}Q-311-T* folds into a (3+1) hybrid quadruplex with a homopolar tetrad stacking and a single all-*syn* G-tract.

The quadruplex is composed of four intervening sequences, namely a 3-nt lateral loop bridging a minor groove followed by two 1-nt propeller loops and a 3-nt lateral snapback loop bridging a wide groove. The thymidine resonances of the two 1-nt propeller loops cannot be distinguished unambiguously. However, various sequential and long-range contacts define the alignment of both lateral loops. In particular, long-range contacts from A5 to C3 and especially to G2 were found in the aromatic-imino but also aromatic-H1' spectral region (not shown). Long-range contacts between G20 H8 and C18 H1' indicate C18 to be located in the groove formed by the all-*syn* and an all-*anti* column. Various NOE contacts between A19 base and sugar protons as well as T17 methyl protons with G imino protons of the lower tetrad hint to T17 and A19 being stacked onto the plane of the outer tetrad (Figure S30E).

Stereospecific assignments of H2'/H2" was achieved through NOESY experiments at short mixing times for the discrimination of H1'-H2' and H1'-H2" crosspeak intensities. Sugar puckers were evaluated by a close inspection of H1'-H2' and H1'-H2" DQF-COSY crosspeaks. Due to anti-phase signals for the active coupling, small coupling constants are expected to result in partial cancellation and weak signal intensities. Thus, all residues except for A5 and G20 were found to adopt a *south*-type sugar pucker. Isochronous H2' and H2" resonances hampered the assignment of the A5 sugar pucker. On the other hand, the H1'-H2" DQF-COSY crosspeak for G20 overlapped with the corresponding crosspeak of C3, yet the H1'-H2' crosspeak intensity suggests at least partially populated *north*-type sugars for G20 (Figure S32).

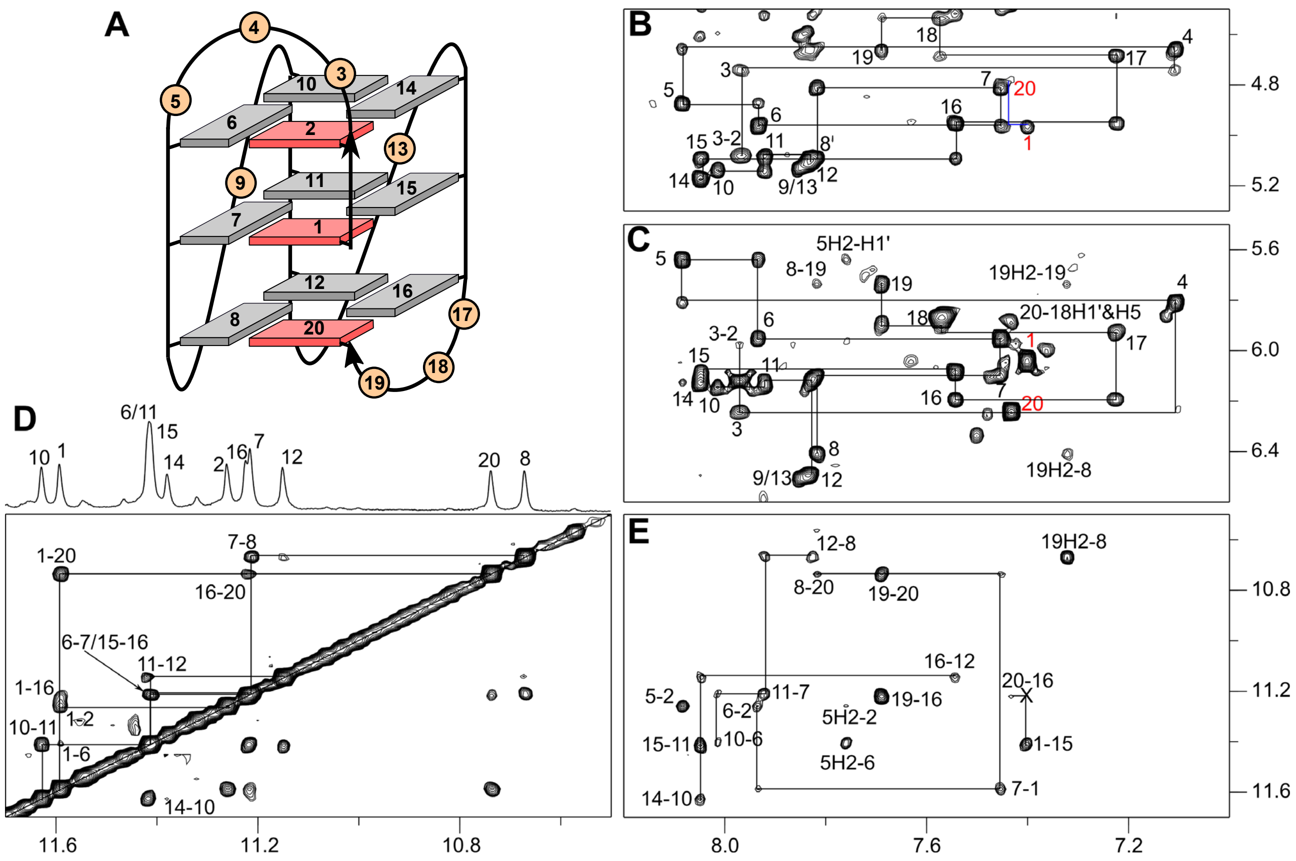

Figure S31. Topology and 2D NOESY spectral regions of *^{2Br}Q-311-T* in 10 mM K^+ buffer, pH 7.0 (30 °C, mixing time 300 ms). (A) Schematic representation with numbered residues of a (3+1) hybrid-type G-quadruplex with a (+lpp) topology and an all-*syn* G-column adopted by *^{2Br}Q-311-T*; *anti*- and *syn*-guanosines of the G-core are colored grey and red, respectively. (B) H6/H8(ω_2)-H3'(ω_1) and (C) H6/H8(ω_2)-H1'(ω_1) 2D NOE spectral regions tracing continuous intra-nucleotide and sequential connectivities; intra-nucleotide crosspeaks of *syn*-guanosines are labelled in red; blue lines in (B) indicate a reversed sequential contact typical for *syn*-*syn* steps. (D) H1(ω_2)-H1(ω_1) crosspeaks with sequential contacts traced along the G tracts. (E) H8/H2(ω_2)-H1(ω_1) NOE contacts with typical intra-tetrad GH8-GH1 connectivities.

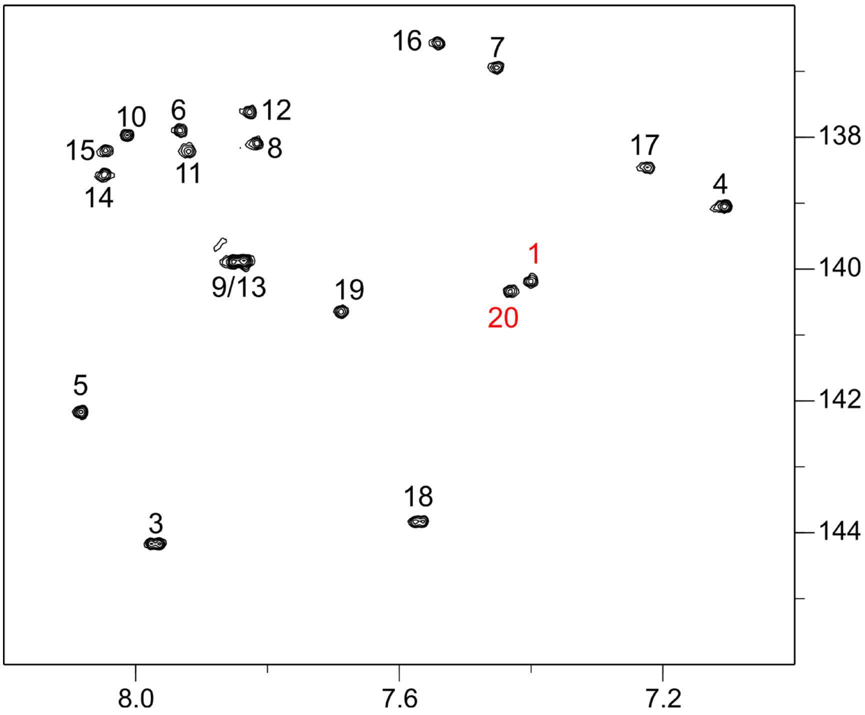

Figure S32. ^1H-^{13}C HSQC spectrum of *^{2Br}Q-311-T* acquired at 30 °C in 10 mM K^+ buffer, pH 7.0, showing H8/H6–C8/C6 correlations. Crosspeaks of *syn*-G1 and *syn*-G20 are labelled in red.

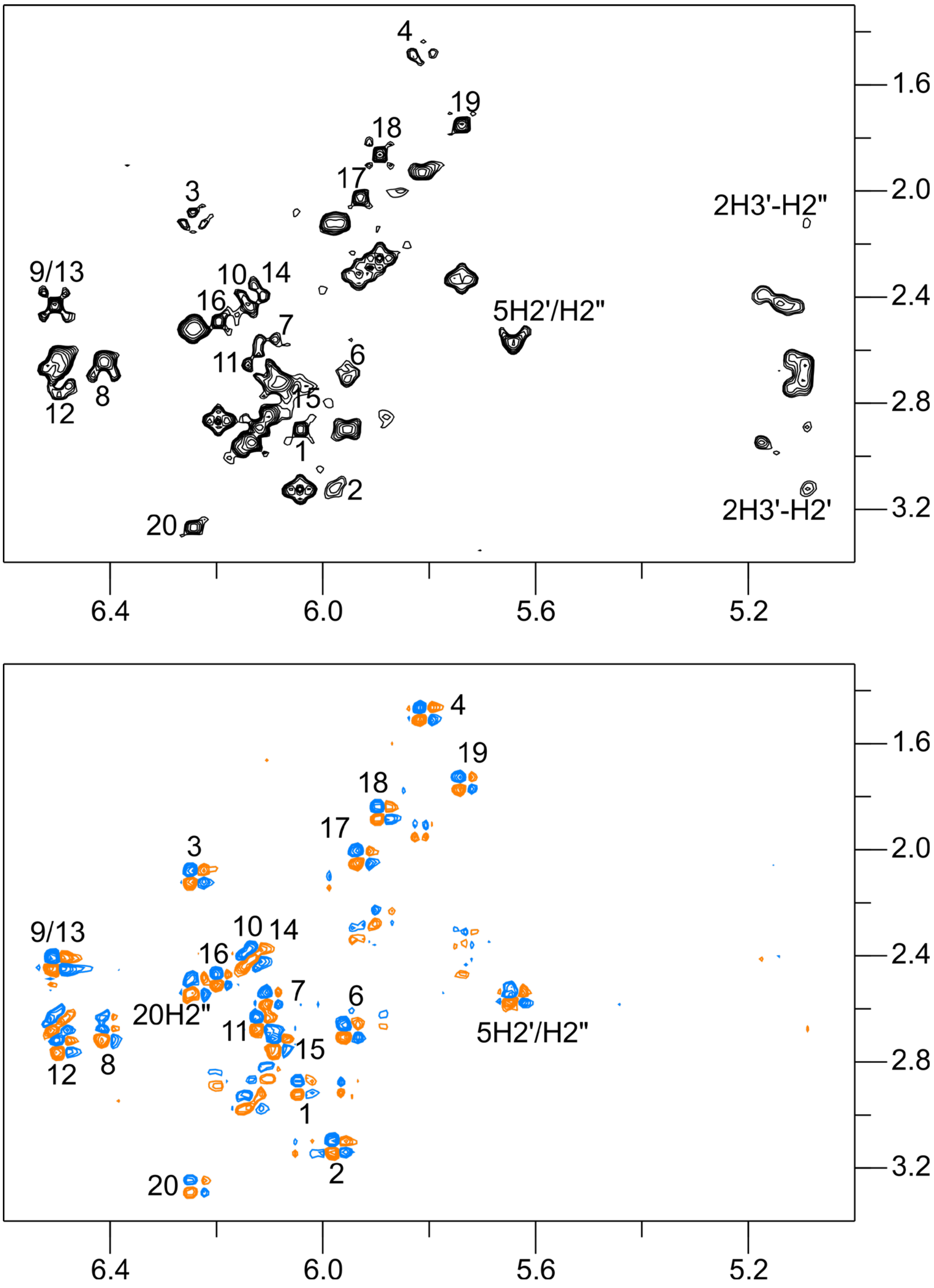

Figure S33. Sugar pucker analysis of *[2Br]Q-311-T*. (Top) Stereospecific assignments of H2'/H2" with H1'(ω_2)-H2'/H2"(ω_1) NOESY spectral region at short mixing time (80 ms); crosspeak intensities allow discrimination between H2' and H2". (Bottom) DQF-COSY spectral region showing H1'(ω_2)-H2'/H2"(ω_1) crosspeaks; *north*- and *south*-type sugar puckers are associated with different scalar couplings and thus different crosspeak patterns of in-phase and anti-phase components.

Table S7. ^{1}H and ^{13}C chemical shifts δ of *^{2Br}Q-311-T*.[a]

δ (ppm)	H8/H6	H1/H3	H1'	H2'/H2"	H3'	H5/H2/Me	C8/C6	C2
G1	7.40	11.59	6.04	2.90/3.13	4.97	-	140.19	-
^{Br}G2	-	11.26	5.98	3.12/2.12	5.08	-	n.d.	-
C3	7.97	-	6.24	2.11/2.53	4.74	6.12	144.17	-
T4	7.11	n.d.	5.81	1.49/1.93	4.66	1.52	139.06	-
A5	8.08	-	5.64	2.56/2.56	4.87	7.76	142.19	154.81
G6	7.93	11.41	5.95	2.68/2.90	4.96	-	137.89	-
G7	7.45	11.21	6.10	2.56/2.85	4.81	-	136.95	-
G8	7.82	10.67	6.41	2.69/2.65	5.09	-	138.10	-
T9	7.85	n.d.	6.50	2.43/2.67	5.13	1.96	139.90	-
G10	8.01	11.63	6.15	2.42/2.95	5.14	-	137.98	-
G11	7.92	11.42	6.12	2.65/2.89	5.08	-	138.22	-
G12	7.83	11.15	6.49	2.74/2.61	5.10	-	137.62	-
T13	7.84	n.d.	6.50	2.43/2.67	5.12	1.96	139.88	-
G14	8.05	11.38	6.13	2.40/2.95	5.17	-	138.58	-
G15	8.05	11.41	6.09	2.74/2.72	5.09	-	138.20	-
G16	7.54	11.22	6.19	2.49/2.87	4.95	-	136.58	-
T17	7.22	n.d.	5.93	2.03/2.32	4.69	1.65	138.48	-
C18	7.57	-	5.93	1.86/2.26	4.53	5.87	143.83	-
A19	7.69	-	5.89	1.75/2.33	4.66	7.32	140.65	152.94
G20	7.43	10.74	6.24	3.27/2.51	4.78	-	140.35	-

[a]At 30 ºC in 10 mM potassium phosphate buffer, pH 7.0.

Table S8. NMR restraints and structural statistics of calculated structures for *[2Br]Q-311-T*

NOE distance restraints	
intra-residual	77
inter-residual	123
exchangeable	42
other restraints:	
hydrogen bonds	48
dihedral angles	38
planarity	3
structural statistics:	
pairwise heavy atom RMSD value (Å)	
all residues	1.5 ± 0.5
G-tetrad core	0.7 ± 0.2
NOE violations:	
maximum violation (Å)	0.14
mean NOE violation (Å)	0.002 ± 0.001
deviations from idealized geometry:	
bond lengths (Å)	0.01 ± 0.0001
bond angles (degree)	2.2 ± 0.02

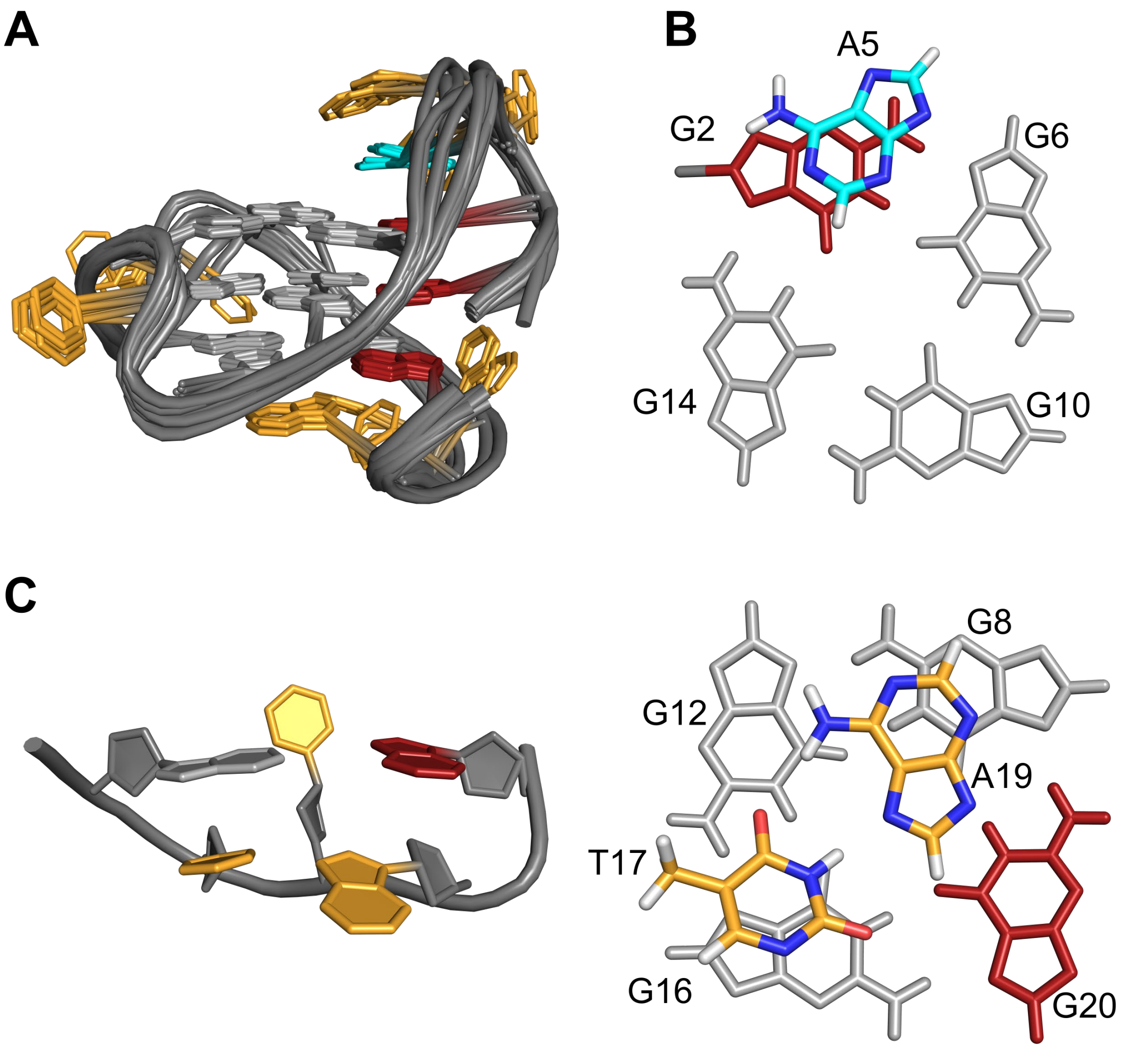

Figure S34. (A) Superposition of ten lowest-energy structures for ^{2Br}*Q-311-T*; *anti*- and *syn*-guanosines are colored in grey and red, respectively, A5 is colored in cyan, and other residues are colored in orange. (B) Stacking of A5 onto the upper G-tetrad. (C) TCA lateral snapback loop of ^{2Br}*Q-311-T* in a side view (left) and top view (right) showing stacking of the T17·A19 Hoogsteen base pair onto the outer G-tetrad. C18 is located in the groove (not shown in top view). Such a capping structure of the T17·A19 base pair can be found in 8 out of 10 structures. In two structures, the snapback loop rearranges to have T17 and C18 stacked on each other while being tilted at about 45° whereas A19 remains efficiently stacked onto the tetrad.

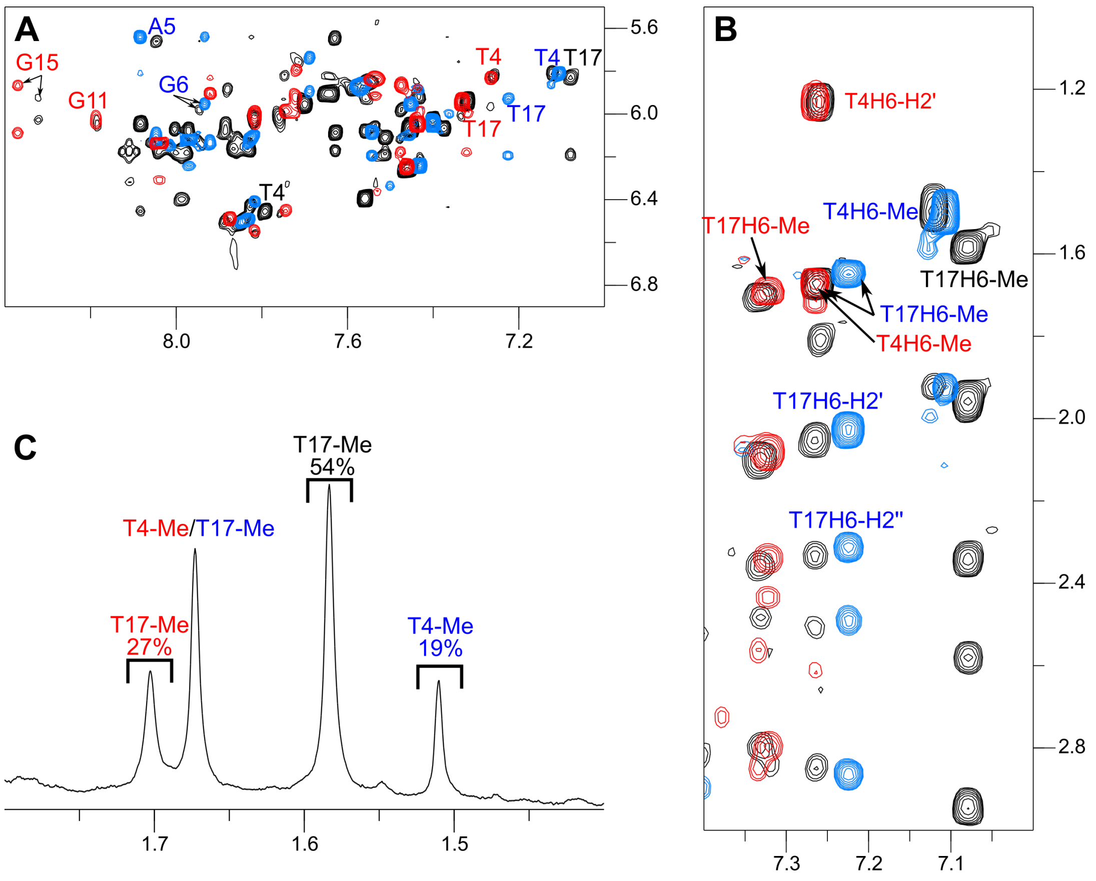

Figure S35. (A) Superimposed H6/8-H1' and (B) H6/8-H2'/Me NOESY spectral regions of *Q-311-T* (black), ^{14Br}*Q311-T* (red) and ^{2Br}*Q-311-T* (blue). (C) Thymine methyl 1D NMR spectral region of *Q-311-T* with integrals given for the specified peaks. Assigned proton resonances in the parallel, hybrid-1R', and hybrid-1R topologies are colored in black, red, and blue, respectively. The T4 methyl of the major parallel species resonates outside the plotted spectral region.

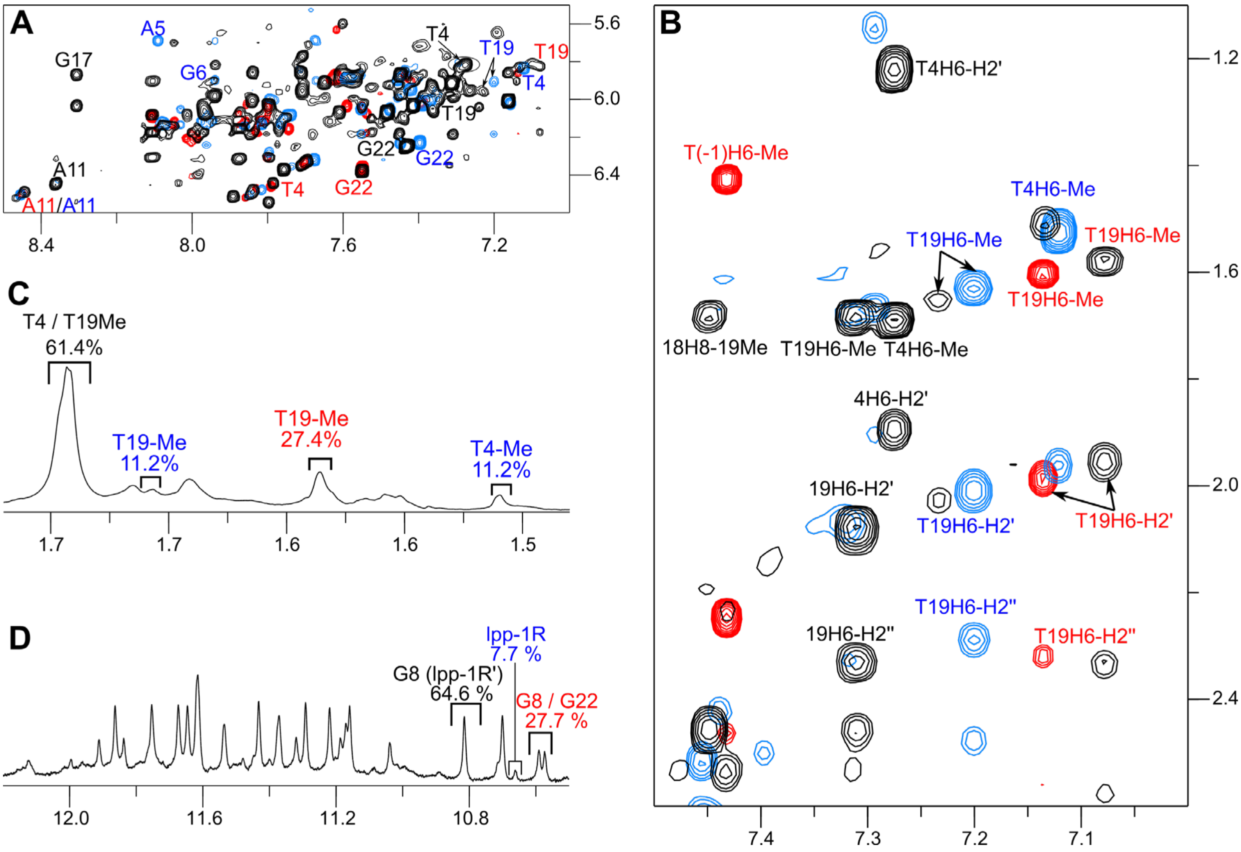

Figure S36. (A) Superimposed H6/8-H1' and (B) H6/8-H2'/Me NOESY spectral regions of *Qref* (black), *5'TQ* (red) and *2BrQ* (blue). (C) Thymine methyl 1D NMR spectral region of *Qref* with integrals given for the specified peaks. Assigned proton resonances in the parallel, hybrid-1R', and hybrid-1R topologies are colored in red, black, and blue, respectively. (D) Imino proton spectral region of *Qref* with topology-specific assignments and integrals of imino resonances in line with the T methyl-based assignments.

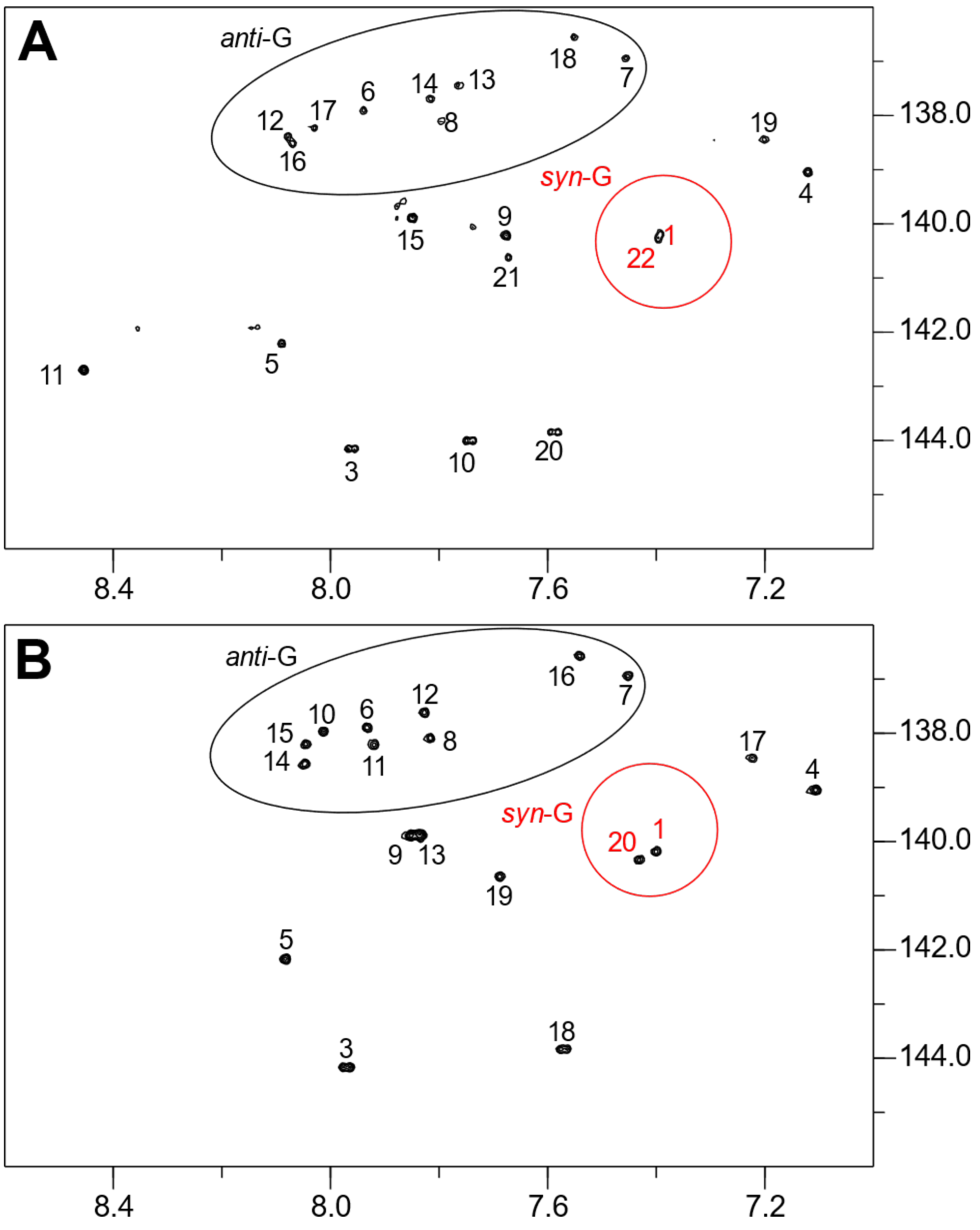

Figure S37. Comparison of ^{1}H-^{13}C HSQC spectra of (A) *^{2Br}Q* and (B) *^{2Br}Q-311-T* acquired at 30 °C in 10 mM K^{+} buffer, pH 7.0, showing H8/H6–C8/C6 correlations. *Anti*-G and *syn*-G residues of the G-core are circled and labelled in black and red color, respectively. The highly similar pattern of C-H correlations comprising two *syn*-G and 9 *anti*-G residues for the G-core demonstrate the same fold for both sequences. Note the absence of a third *syn*-G residue at position 2 due to ^{Br}G incorporation.
